# Epicatechin oligomers longer than trimers have anti-cancer activities, but not the catechin counterparts

**DOI:** 10.1038/s41598-017-08059-x

**Published:** 2017-08-10

**Authors:** Kohki Takanashi, Manato Suda, Kiriko Matsumoto, Chisato Ishihara, Kazuya Toda, Koichiro Kawaguchi, Shogo Senga, Narumi Kobayashi, Mikihiro Ichikawa, Miyuki Katoh, Yasunao Hattori, Sei-ichi Kawahara, Koji Umezawa, Hiroshi Fujii, Hidefumi Makabe

**Affiliations:** 1 Graduate School of Agriculture, Sciences of Functional Foods, Shinshu University, 8304 Minami-minowa Kami-ina, Nagano, 399-4598 Japan; 2 Department of Bioscience and Biotechnology, Faculty of Agriculture, Shinshu University, 8304 Minami-minowa Kami-ina, Nagano, 399-4598 Japan; 3 Department of Biomedical Engineering, Graduate School of Science and Technology, Shinshu University, 8304 Minami-minowa Kami-ina, Nagano, 399-4598 Japan; 4 Interdisciplinary Graduate School of Science and Technology, Shinshu University, 8304 Minami-minowa Kami-ina, Nagano, 399-4598 Japan; 5 Center for Instrumental Analysis, Kyoto Pharmaceutical University, Yamashina-ku, Kyoto, 607-8412 Japan; 6 Department of Interdisciplinary Genome Sciences and Cell Metabolism, Institute for Biomedical Sciences, Interdisciplinary Cluster for Cutting Edge Research, Shinshu University, Minami-minowa, Kami-ina, Nagano, 399-4598 Japan

## Abstract

Since procyanidins (oligomeric catechin or epicatechin) were reported to exhibit health benefits, much attention has been paid to the synthesis of these compounds, especially those that are longer than trimers. In the present study, syntheses of cinnamtannin A3 (epicatechin pentamer), A4 (epicatechin hexamer), catechin tetramer, pentamer, arecatannin A2 (epicatechin-epicatechin-epicatechin-catechin) and A3 (epicatechin-epicatechin-epicatechin-epicatechin-catechin) were achieved. The key reaction was a Lewis acid mediated equimolar condensation. The antitumor effects of these synthesized compounds against a human prostate cancer cell line (PC-3) were investigated. Among the tested compounds, cinnamtannin A3, A4 and arecatannin A3, which possess epicatechin oligomers longer than tetramers as the basic scaffold, showed significant activities for suppression of cell growth, invasion and FABP5 (fatty acid-binding protein 5) gene expression. Effects on cell cycle distribution showed that cell cycle arrest in the G2 phase was induced. Furthermore, these epicatechin oligomers suppressed significantly the expression of the cancer-promoting gene, *FABP5*, which is related to cell proliferation and metastasis in various cancer cells. Interestingly, the suppressive activities were associated with the degree of oligomerization of epicatechin. Thus, synthetic studies clearly demonstrate that epicatechin oligomers longer than trimers have significant anti-tumorigenic activities, but not the catechin counterparts.

## Introduction

**Figure 1:**
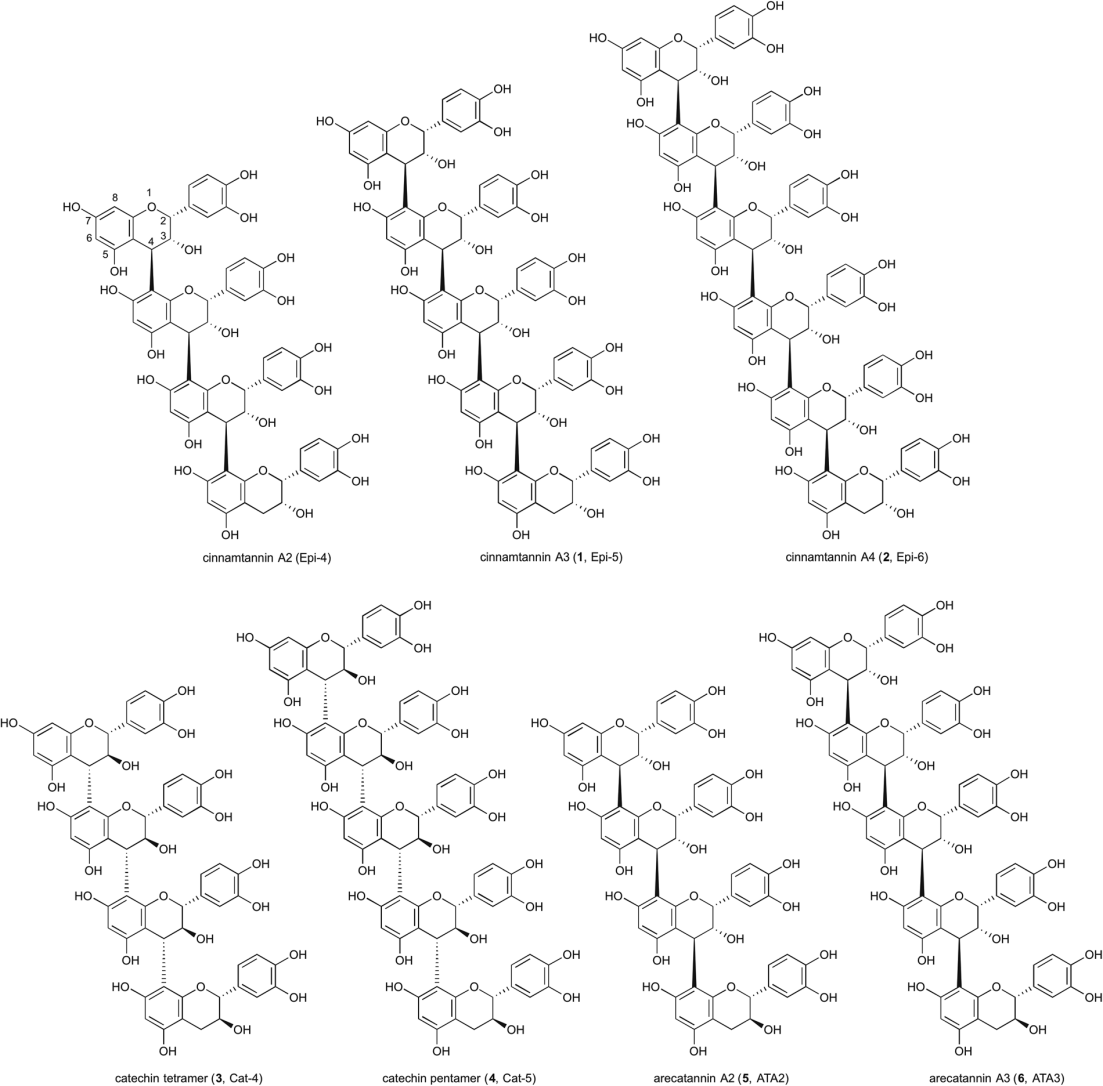
The structures of synthesized catechin and epicatechin oligomers. Syntheses of cinnamtannin A3 (epicatechin pentamer, Epi-5), A4 (epicatechin hexamer, Epi-6), catechin tetramer (Cat-4), pentamer (Cat-5), arecatannin A2 (epicatechin-epicatechin-epicatechin-catechin, ATA2) and A3 (epicatechin-epicatechin-epicatechin-epicatechin-catechin, ATA3) were achieved.

Among the large family of tannins, procyanidins are the most abundant species found in plants such as vegetables, fruit and the bark of trees^1, 2^. These compounds contain flavan-3-ol as a basic structural unit and are attracting attention for their health benefits, such as the suppression of endotherin-1 synthesis^3^. Procyanidins have been reported to exhibit a variety of biological activities such as antitumor^4, 5^, antiviral^6^ and anti-inflammatory^7^ activities. These compounds have wide structural diversity because they have different monomeric constituents such as (+)-catechin and (−)-epicatechin, and variable degrees of oligomerization. In addition, the connectivity between the flavan units differs such as C-4 to C-8 and C-4 to C-6 internal flavan bonds (Fig. 1). To obtain oligomeric procyanidins in a pure state for biological studies, a variety of pure and structurally determined synthetic standard compounds are required. Currently, synthetic efforts have been carried out to obtain pure procyanidin oligomers^8–11^. However, reports of the synthesis of catechin and/or epicatechin oligomers, especially those longer than tetramers are rather limited. Synthetic methods of procyanidin oligomers were reported by Saito, Nakajima and co-workers^12^, and Ohmori, Suzuki and co-workers^13^. The method by the former group used excess amounts of a nucleophilic partner (ca. 4.0 eq.) for condensation to prevent further oligomerization. The disadvantage of this procedure is that the excess nucleophilic partner must be removed after condensation. The other method reported used a C-8 bromide derivative to prevent the formation of further oligomerization.

Various types of proanthocyanidins have been synthesized to evaluate their biological activities. The synthesis of procyanidin dimers such as procyanidin B1, B2, B3 and B4 using Yb(OTf)_3_ mediated equimolar condensation^14^ and their content in apple juice^15^ has been reported by us. The anti-inflammatory activity of these compounds has also been reported^16, 17^. Recently, the synthesis of procyanidin trimers such as procyanidin C1 and C2 using Yb(OTf)_3_ or silver Lewis acid mediated equimolar condensation was presented^18, 19^. Currently, minimal effort has been made to the screening of Lewis acids for *equimolar* condensation to construct the skeleton of procyanidin oligomers. Synthesis of catechin and epicatechin oligomers using an equimolar condensation approach has not been reported. In this article, the total syntheses of an epicatechin pentamer, named cinnamtannin A3 (**1**, Epi-5), a catechin tetramer (**3**, Cat-4), pentamer (**4**, Cat-5), epicatechin-epicatechin-epicatechin-catechin, named arecatannin A2 (**5**, ATA2), epicatechin-epicatechin-epicatechin-epicatechin-catechin named arecatannin A3 (**6**, ATA3) via *equimolar* condensation between a catechin or epicatechin nucleophile and a catechin or epicatechin electrophile are reported. As to the cinnamtannin A4 (**2**, Epi-6), 1.7 eq. of nucleophile was required to obtain satisfied yield. The present study also reveals that epicatechin oligomers longer than trimers, but not the catechin counterparts, have significant anti-tumorigenic activities against human prostate cancer cells.

## Results

### Synthesis of cinnamtannin A3 (1, epicatechin pentamer, Epi-5) and A4 (2, epicatechin hexamer, Epi-6)

**Figure 2:**
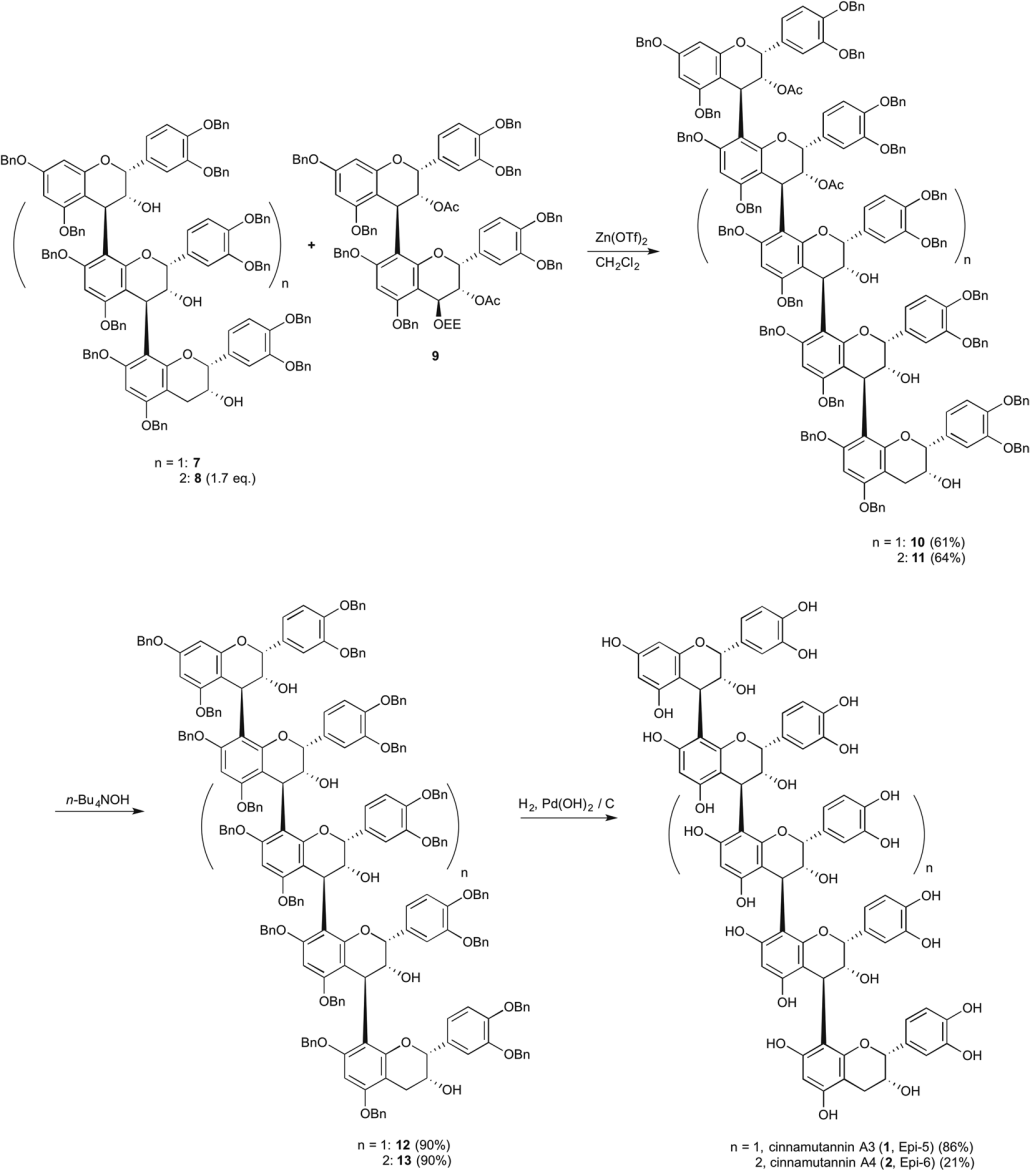
Synthesis of cinnamtannin A3 (**1**, Epi-5) and A4 (**2**, Epi-6). Cinnamtannin A3 (**1**, Epi-5) and A4 (**2**. Epi-6) were synthesized using Zn(OTf)_2_ mediated condensation. Ac, acetyl, Bn, benzyl, Bu, butyl, EE, ethoxyethyl, Zn(OTf)_2_, HPLC, high performance liquid chromatography, zinc(II) trifluoromethenesulfonate, Bn, benzyl.

For the synthesis of cinnamtannin A3 (**1**. Epi-5), equimolar condensation of trimeric nucleophile **7** with dimeric electrophile **9**, which was prepared previously^20^, was examined using Zn(OTf)_2_ in CH_2_Cl_2_. We found that 3.0 eq. of Zn(OTf)_2_ for 21 h gave the condensed product in 61% yield (see, Supplementary Table 1). Hydrolysis of the diacetate of **10** using *n*-Bu_4_NOH afforded **12** in 90% yield^5^. The benzyl groups of **12** were deprotected by hydrogenolysis over Pearlman’s catalyst followed by lyophilization to afford cinnamtannin A3 (**1**, Epi-5) in good yield. The optical rotation value, ^1^H and ^13^C NMR spectral data of synthetic **1** were in good agreement with reported values^5, 12^. For the synthesis of cinnamtannin A4 (**2**), equimolar condensation of tetrameric nucleophile **8** with dimeric electrophile **9** using a Lewis acid in CH_2_Cl_2_ was performed. It was found that 5.0 eq. of Zn(OTf)_2_ afforded condensed product **12** in 27% yield along with 70% recovery of the stating material **8**. Thus, 1.7 eq. of nucleophile **8** was used to afford condensed product **11** in 64% yield. Other Lewis acids such as Yb(OTf)_3_ and AgOTf gave **11** in very low yield. Deprotection of the acetyl groups of **11** with *n*-Bu_4_NOH afforded **13**. The ^1^H and ^13^C NMR spectral data of **13** were in good agreement with reported values^5^. Subsequent hydrogenolysis of the benzyl groups afforded cinnamtannin A4 (**2**, Epi-6) in 21% yield. The reason for the low yield at the deprotection steps is the cleavage of inter-flavan bonds during Pd(OH)_2_ catalyzed hydrogenolysis of the benzyl groups. We found that the reactivity of the hydrogenolysis of the benzyl group of **13** was quite low probably because of steric hindrance caused by highly oligomerization. As a result, the cleavage of inter-flavan bonds took place at the same time. The specific rotation value of synthetic **2** was in good accordance with that of the reported value^5^. Although the ^1^H NMR data of **2** showed broad peaks, the ESI-TOFMS spectra of **2** supports the structure (Fig. 2)^5^.

### Synthesis of the catechin tetramer (3, Cat-4) and pentamer (4, Cat-5)

**Figure 3:**
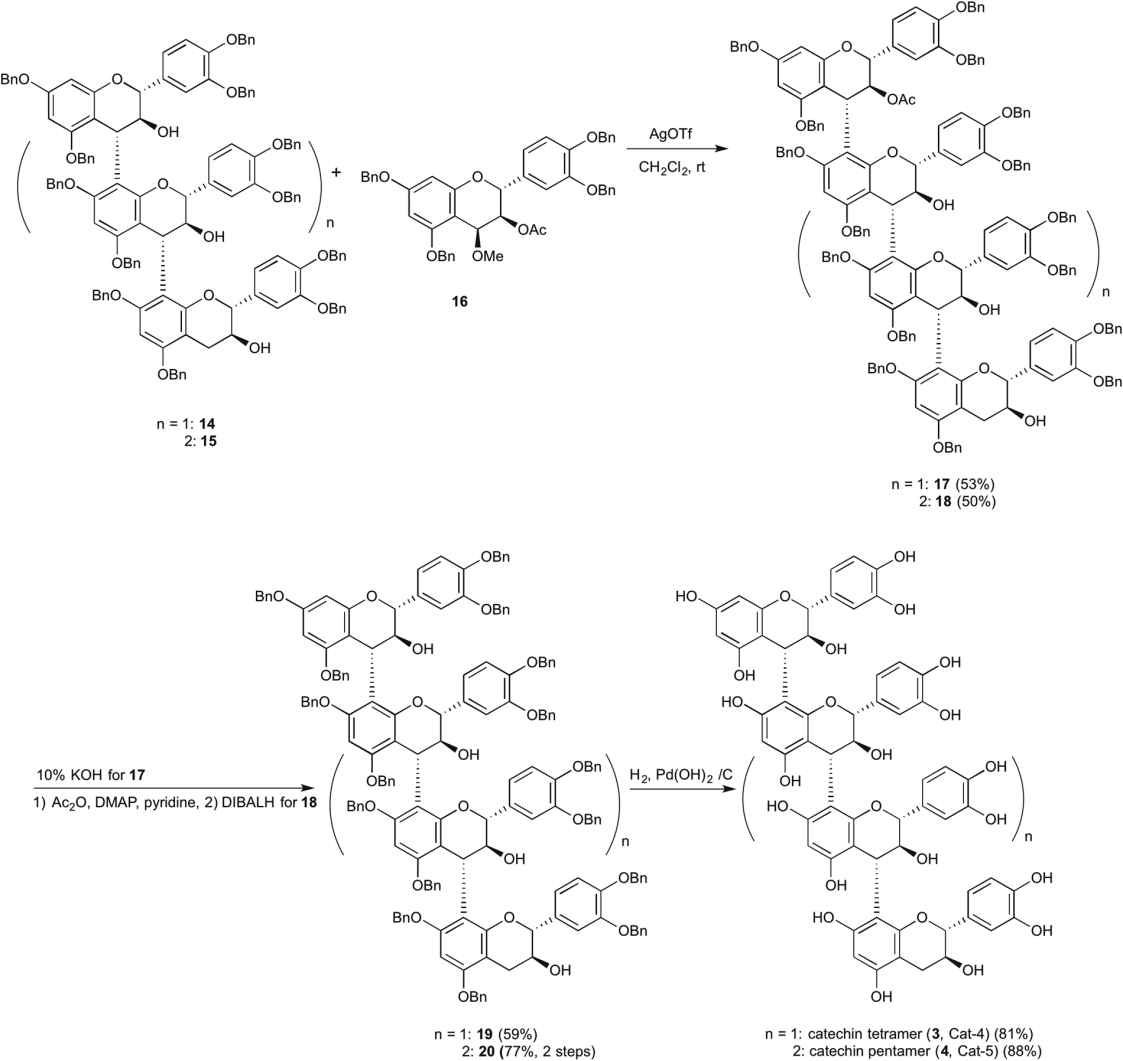
Synthesis of catechin tetramer (**3**, Cat-4) and pentamer (**4**, Cat-5). Catechin tetramer (**3**, Cat-4) and pentamer (**4**, Cat-5) were synthesized using AgOTf mediated equimolar condensation. AgOTf, silver trifuluoromethanesulfonate, DIBALH, diisobutylaluminium hydride, DMAP, N, N, dimethyl-4-aminopyridine.

Equimolar condensation of trimeric nucleophile **14**, which was prepared previously^19^, with **16** was examined using silver Lewis acids and Yb(OTf)_3_ in CH_2_Cl_2_. AgBF_4_ gave the condensed product **17** in low yield. Next, 1.5 eq. of AgOTf was used and a longer reaction time to give the condensed product in a moderate yield. Using 3.0 eq. of Lewis acid gave sluggish results (see, Supplementary Table 2). The condensed product **17** was transformed into tetraol **19** using 10% KOH. The ^1^H and ^13^C NMR spectral data of **19** were in good agreement with reported values^13^. Finally, deprotection of the benzyl group of **19** and subsequent lyophilization afforded the catechin tetramer (**3**, Cat-4) in good yield. As for catechin pentamer (**4**), equimolar condensation of tetrameric nucleophile **15** with monomeric electrophile **16** was examined using Lewis acids in CH_2_Cl_2_. Zn(OTf)_2_ gave the condensed product **18** in low yield. Thus, 3.0 eq. of AgOTf was used and a longer reaction time to give the condensed product in a moderate yield (see, Supplementary Table 3). We found that Zn(OTf)_2_ or Yb(OTf)_3_ were suitable Lewis acid to construct epicatechn oligomer. On the other hand, silver Lewis acids were suitable for catechin oligomer probably because of three dimensional structural difference to activate elimination groups. Acetylation of **18** using Ac_2_O in the presence of catalytic amounts of DMAP in pyridine to afford pentaacetate was followed by reduction of all acetyl groups to give **20** in 77% yield for 2 steps. The benzyl groups of **20** were deprotected by hydrogenolysis over Pearlman’s catalyst followed by lyophilization to afford catechin pentamer (**4**, Cat-5) in good yield (Fig. 3).

### Synthesis of arecatannin A2 (5, ATA2) and A3 (6, ATA3)

**Figure 4:**
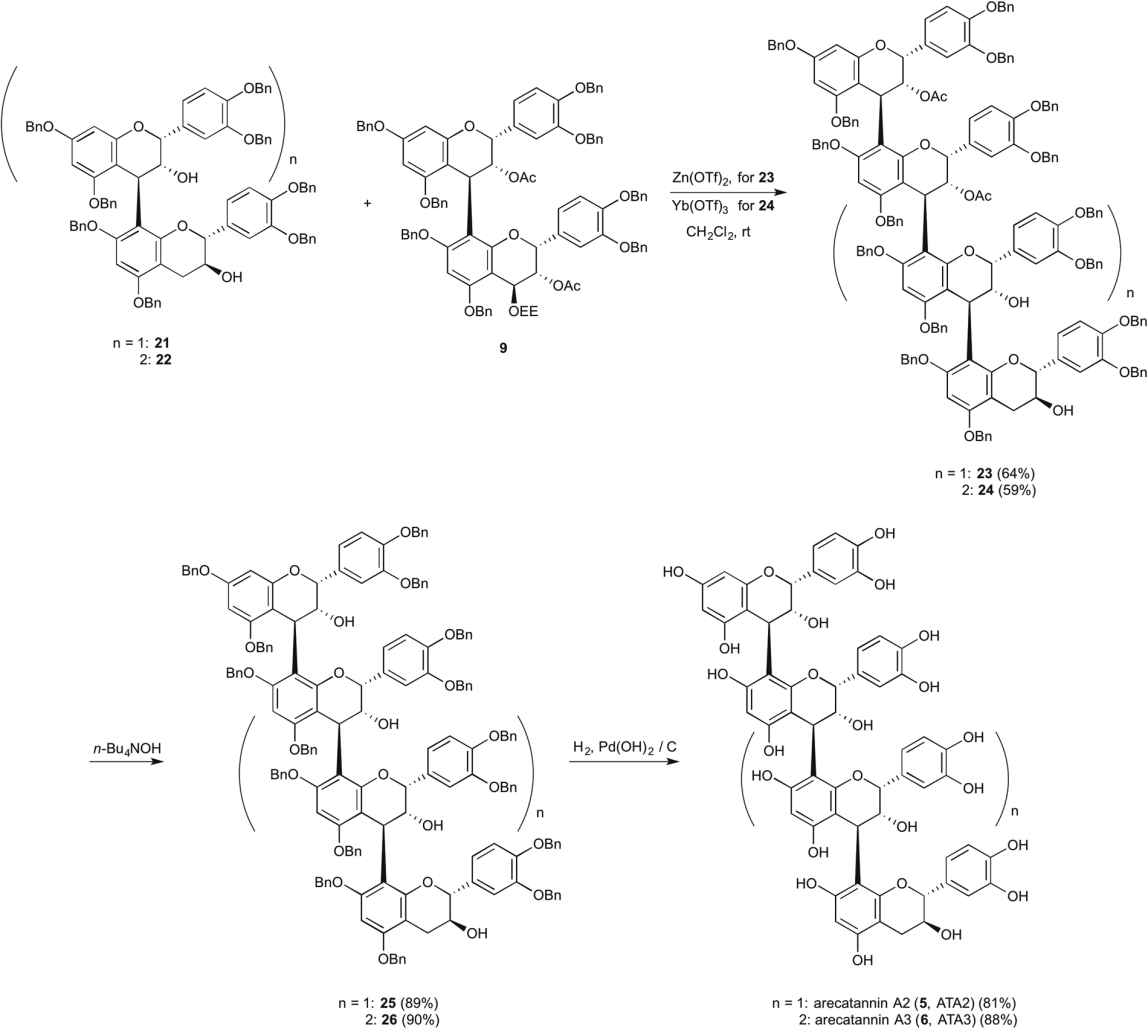
Synthesis of arecatannin A2 (**5**, ATA2) and A3 (**6**, ATA3). Arecatannin A2 (**5**, ATA2) and A3 (**6**, ATA3) were synthesized using Zn(OTf)_2_ and Yb(OTf)_3_ mediated equimolar condensation. Yb(OTf)_3_, ytterbium trifuluoromethanesulfonate.

As for arecatannin A2 (**5**, ATA2), we have investigated Zn and Yb Lewis acids for equimolar condensation between an epicatechin-catechin nucleophile **21**, which was prepared previously^14^, and a dimeric epicatechin electrophile **9** ^20^. Among the Lewis acids tested, it was found that 5.0 eq. of Zn(OTf)_2_ gave condensed product **23** in 64% yield (see, Supplementary Table 4). Hydrolysis of the diacetate of **23** using *n*-Bu_4_NOH afforded **25** in 89% yield^5^. The benzyl groups of **25** were deprotected by hydrogenolysis over Pearlman’s catalyst followed by lyophilization to afford arecatannin A2 (**5**, ATA2) in good yield. The optical rotation value, ^1^H and ^13^C NMR spectral data and mass spectrum data of synthetic **5** were in good accordance with the reported values^12^. As for arecatannin A3 (**6**, ATA3), we have investigated Zn and Yb Lewis acids for equimolar condensation between an epicatechin-epicatechin-catechin nucleophile **22**, which was synthesized previously^21^, with the dimeric epicatechin electrophile **9**. Among the Lewis acids tested, it was found that 5.0 eq. of Yb(OTf)_3_ gave condensed product **24** in 59% yield (see, Supplementary Table 5). Hydrolysis of the diacetate of **24** using *n*-Bu_4_NOH afforded **26** in 90% yield^5^. The benzyl ether of **26** was deprotected by hydrogenolysis over Pearlman’s catalyst followed by lyophilization to afford arecatannin A3 (**6**, ATA3) in good yield. The optical rotation value, ^1^H and ^13^C NMR spectral data of synthetic **6** were in good agreement with previously reported values (Fig. 4)^12^.

### Epicatechin oligomers longer than trimers suppress cell proliferation of PC-3 prostate cancer cells

**Figure 5:**
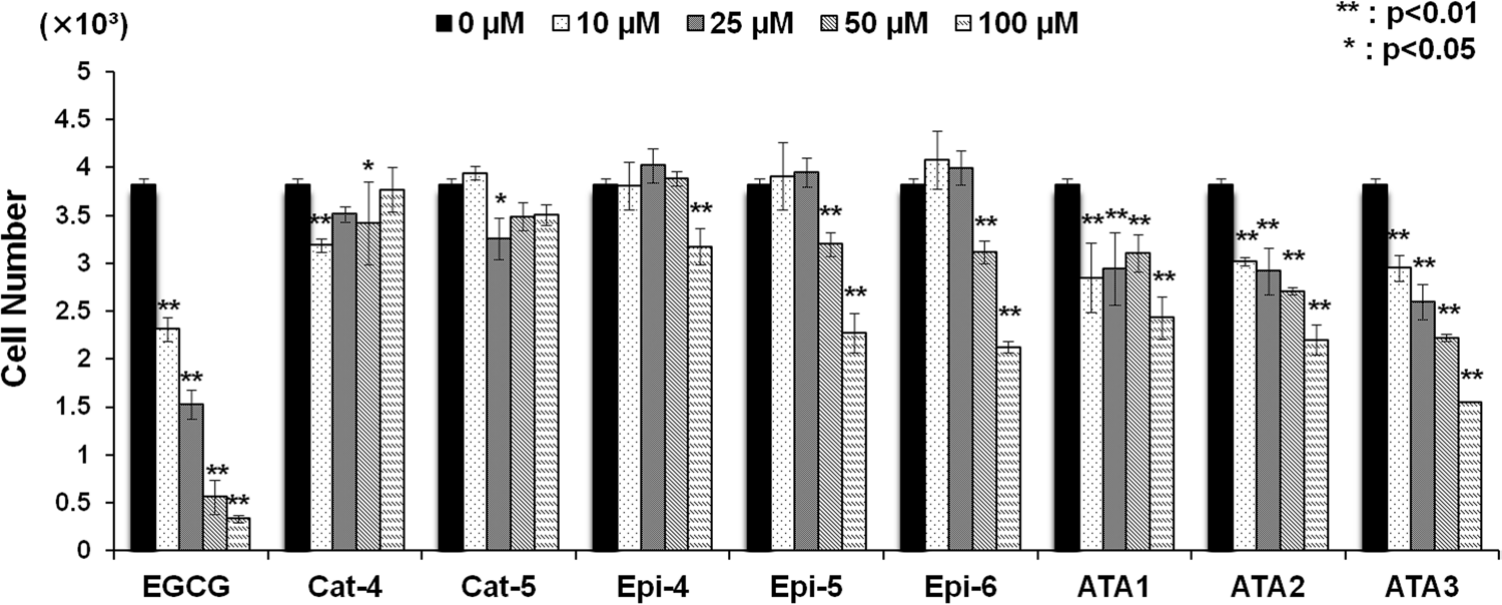
Effects of various concentrations of test compounds on PC-3 prostate cancer cell proliferation. Effects of various concentrations of test compounds on PC-3 cancer cell proliferation using a cell count method. The experimental procedure for preparation of test compounds was described in Biochemical methods of Supplementary Information. After treatment of cells with either the catechin tetramer (Cat-4), catechin pentamer (Cat-5), cinnamtannin A2 (Epi-4), cinnamtannin A3 (Epi-5), cinnamtannin A4 (Epi-6), arecatannin A1 (ATA1), arecatannin A2 (ATA2) or arecatannin A3 (ATA3) for 48 h, cell proliferation was determined by cell counting as shown in the experimental section. The values are presented as the rate of inhibition of cell proliferation by the treated sample when compared with that of the untreated control (vehicle). Values are means ± S.Ds. for three independent experiments. Two-way ANOVA followed by Dunnett’s multiple comparison test was used to compare means. *P < 0.05 or **P < 0.01.

**Figure 6:**
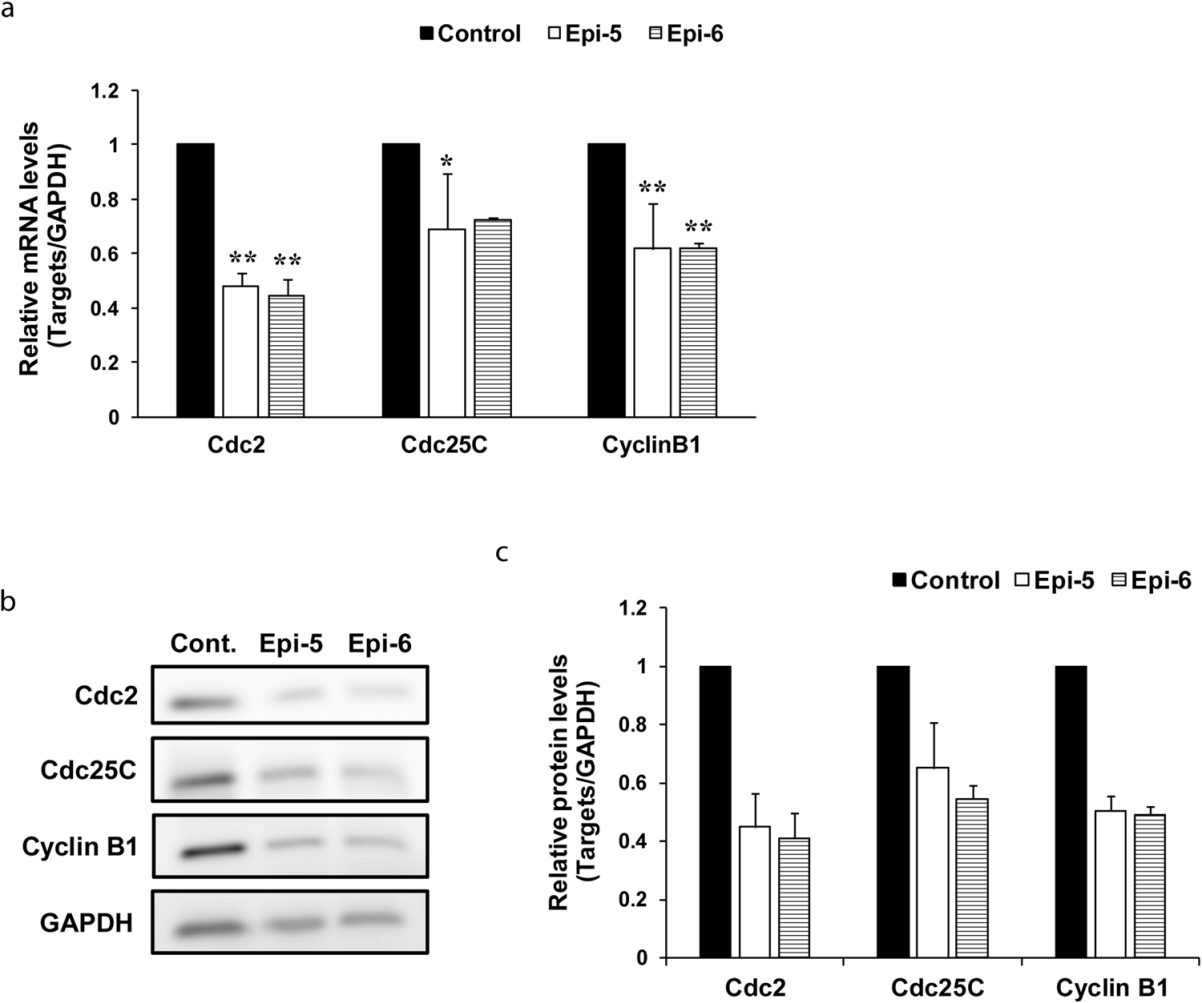
Effects of test compounds on the expression of the cell cycle-related genes. Cells were cultured as described in Biochemical methods and treated with ethanol (6% v/v) alone (control) and 50 μmol/L test compounds. The experimental procedure for preparation of test compounds was described in Biochemical methods of Supplementary Information. (**a**, **b**, **c**). Test oligomeric compounds, epicatechin pentamer (Epi-5) and epicatechin hexamer (Epi-6) significantly suppressed the expression of Cdc2, Cdc25C and Cyclin B1 at mRNA and protein levels in PC-3 cells. The cells treated with test compounds for 48 h were collected and the mRNA level was determined by qPCR (**a**). Whole cell lysates were prepared and subjected to western blotting. Western blot analysis of protein levels (Cdc2, Cdc25C and Cyclin B1) was determined after treatment of PC-3 cells with epicatechin pentamer (Epi-5) and hexamer (Epi-6) (**b**, **c**). The data of Western blot analysis are representative of three independent experiments. The quantification of these protein levels by densitometric measurement using Image J software (NIH) (**c**). The data of qPCR and densitometric data from western blot analysis are the means ± S.D. of three independent experiments, and one-way ANOVA followed by Tukey’s multiple comparison test was used to compare means. *P < 0.05 or **P < 0.01.

Our interest was focused on examining the antitumor activities of the newly synthesized procyanidins. Procyanidins with basic scaffolds less than trimers have not been shown to have any activities for suppression of cell growth and apoptosis induction^22^. Thus, tetramer and pentamer species were tested for anti-cancer activity. The synthesis of compounds **1**–**6** allowed us to obtain sufficient quantities of purified compounds to screen against PC-3 prostate cancer cell lines together with arecatannin A1 (ATA1)^21^ and cinnamtannin A2 (Epi-4)^23^, which were prepared previously (Fig. 5). Results were obtained by cell count measurement. (−)-Epigallocatechin-3-gallate (EGCG) was used as a positive control. As shown in Fig. 5, epigallocatechin gallate (EGCG), cinnamtannin A3 (Epi-5, **1**), cinnamtannin A4 (Epi-6, **2**) and arecatannin A3 (ATA3, **6**) exhibited significant cell growth inhibitory activity. Inhibitory effects on cell growth were clearly associated with the degree of oligomerization of epicatechin (tetramer, pentamer and hexamer) (Fig. 5). Although cell growth inhibitory activity of the epicatechin oligomers longer than trimers might be partially attributed to cell cycle arrest (Fig. 6), further mechanistic study should be needed. Interestingly, no activity was observed for the catechin tetramer (Cat-4) and pentamer (Cat-5), probably because of differences in the three-dimensional structures (Fig. 9). Dimeric and trimeric procyanidins, which others have reported to show activity on small cell lung, colorectal cancer cell lines and skin tumor promotion in mouse epidermis *in vivo* ^24, 25^, showed no activity in prostate cancer cell lines.

### Pentameric procyanidins induce cell cycle arrest in PC-3 prostate cancer cells

Cell growth and proliferation are related to the cell cycle progression. In this study with FACS analysis, treatment of PC-3 prostate cancer cells with cinnamtannin A3 (Epi-5, **1**) for 48 h induced an increase in the G2 phase population from 24.59% to 41.30% and an S phase fraction decrease from 16.67% to 10.69%. Epi-5 blocked the PC-3 prostate cancer cell cycle at the G2 phase within 48 h (Supplementary Figure 1). A similar tendency was observed in ATA3 (data not shown). A lower S phase population is indicative of a slower cell division rate and slower tumor growth. We also investigated whether the epicatechin oligomers (Epi-5 and Epi-6) induce G2/M phase arrest by changing the mRNA and protein levels of G2/M phase cell cycle regulators (Cdc2, Cdc25C and Cyclin B1). The results showed that treatment of PC-3 cells with Epi-5 or Epi-6 for 48 h at a dose of 50 μmol/L significantly decreased these mRNA and protein levels in comparison with the control (Fig. 6), suggesting that the G2/M cell cycle arrest might be induced in PC-3 cells. Interestingly, these results are different from those of Kozikowski and co-workers who reported that treatment of human breast cancer cells (MDA MB 231) with Epi-5 induced G1/G0 arrest^5^. However, additional efforts are required to elucidate the mechanisms of action (Fig. 6).

### Epicatechin oligomers longer than trimers suppress expression of the cancer-promoting gene, *FABP5*

Previous work by Fujii and co-workers reported that the *FABP5* gene was highly expressed and involved in metastasis in prostate cancer cells^26, 27^.

**Figure 7:**
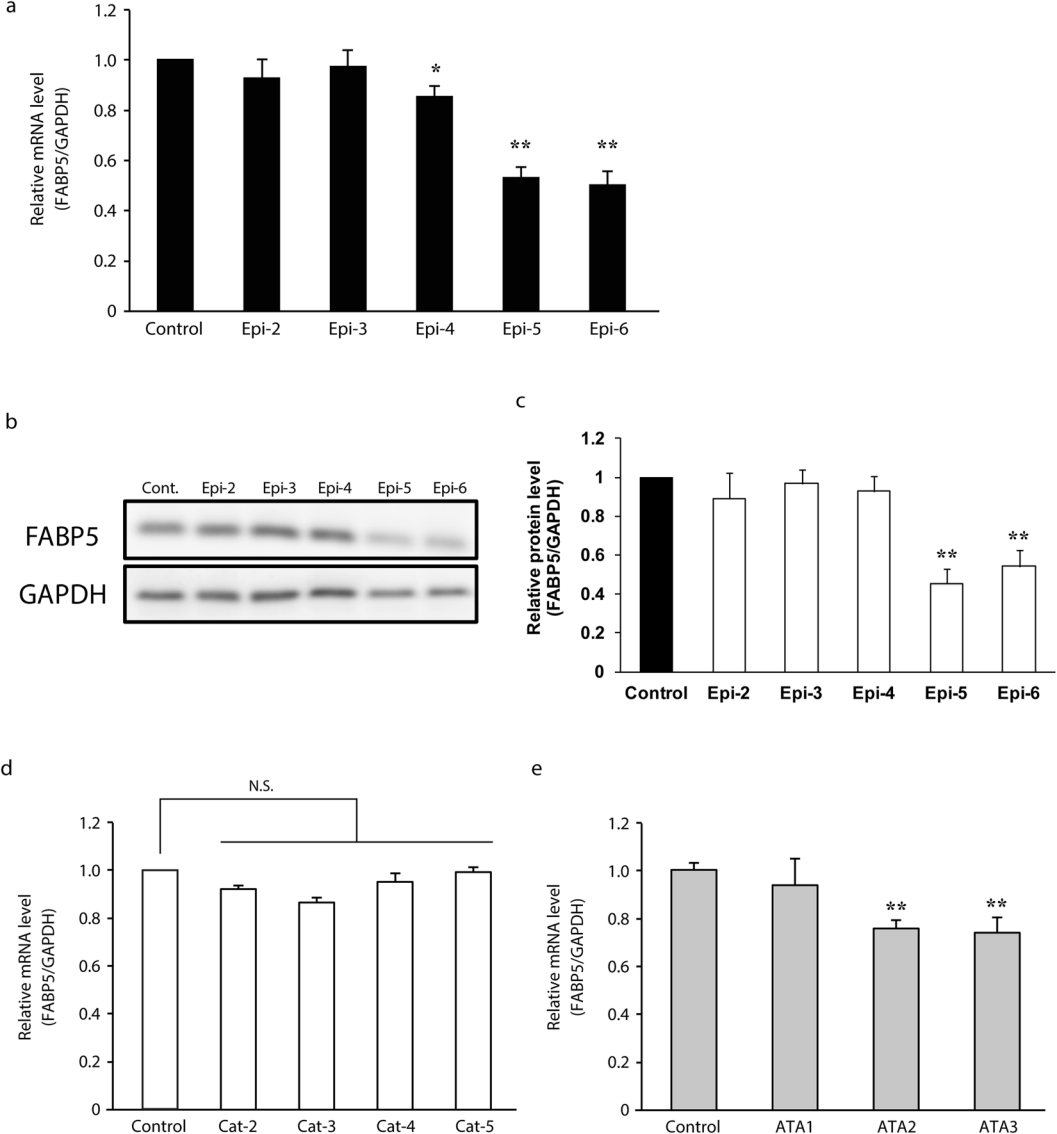
Effects of test compounds on the expression of the cancer-promoting gene *FABP5*. Of test oligomeric compounds (50 μmol/L), epicatechin pentamer (Epi-5) strongly suppressed the FABP5 gene expression in PC-3 cells. The experimental procedure for preparation of test compounds was described in Biochemical methods of Supplementary Information. The cells treated with test compounds, epicatechin [dimer (Epi-2), trimer (Epi-3), tetramer (Epi-4), pentamer (Epi-5) and hexamer (Epi-6)] (**a**, **b** and **c**), catechin [dimer (Cat-2), trimer (Cat-3), tetramer (Cat-4) and pentamer (Cat-5)] (**d**), arecatannin A1 (ATA1), arecatannin A2 (ATA2), or arecatannin A3 (ATA3) (**e**) for 48 h were collected and the FABP5 mRNA level was determined by qPCR (**a**, **d** and **e**). Whole cell lysates were prepared and subjected to western blotting. Western blot analysis of FABP5 protein levels was determined after treatment of PC-3 cells with epicatechin [dimer (Epi-2), trimer (Epi-3), tetramer (Epi-4), pentamer (Epi-5) and hexamer (Epi-6)] (**b** and **c**). The quantification of FABP5 protein levels by densitometric measurement using Image J software (NIH) (**c**). The data of qPCR and densitometric data from western blot analysis are the means ± S.D. of three independent experiments, and one-way ANOVA followed by Tukey’s multiple comparison test was used to compare means. *P < 0.05 or **P < 0.01. The data of Western blot analysis are representative of three independent experiments.

More recently, the *FABP5* gene has been shown to be epigenetically regulated during human prostate carcinogenesis^28^ and that high-expression of *FABP5* is responsible for the promotion of cell growth and invasion in various cancer cells^26, 29^, suggesting that it plays a critical role in tumorigenesis of various cancer cells. Altered fatty acid metabolism is thought to be a hallmark of cancer^30^. Especially, prostate cancer represents lipogenic phenotype and utilizes fatty acid oxidation as a dominant bioenergetics pathway to support proliferation^31–33^. *FABP5* might be responsible for fatty acid metabolism as a lipid transporter and/or an important regulatory factor, suggesting that its critical role in metabolic alterations of fatty acid metabolism in prostate cancer. Indeed, we have found that siRNA-mediated knockdown of *FABP5* expression significantly decreased fatty acid-metabolizing enzymes^29^ and metastasis^26^. Therefore, we have been screening potential anti-cancer agents by assessing inhibitory activity for the gene expression of *FABP5*. Thus, the compounds which suppress the expression of *FABP5* might be promising chemopreventive agents against prostate cancer metastasis. As shown in Fig. 7, Epi-5 significantly suppressed the expression of *FABP5* at mRNA and protein levels. ATA2 and ATA3, which possess the catechin unit at the end of oligomers, showed weaker activities than Epi-5. Interestingly, no suppressive activity was observed for the Cat-4 and Cat-5 probably because of differences in the three-dimensional structures, as mentioned in Fig. 9. It would be interesting to examine whether a putative target molecule interacting with the epicatechin oligomer (e.g. Epi-5), but not with the catechin counterparts, showed negligible suppressive activities of *FABP5* gene expression. Further studies are required to investigate how to suppress expression of this gene by these compounds. In addition, as the epicatechin oligomer (e.g. Epi-5) has been shown to strongly suppress the expression oncogenic genes other than *FABP5*, it would be interesting to investigate the regulatory mechanisms underlying suppression of these gene expressions by Epi-5.

### Epicatechin oligomers longer than trimers suppress invasive activity of PC-3 prostate cancer cells

**Figure 8:**
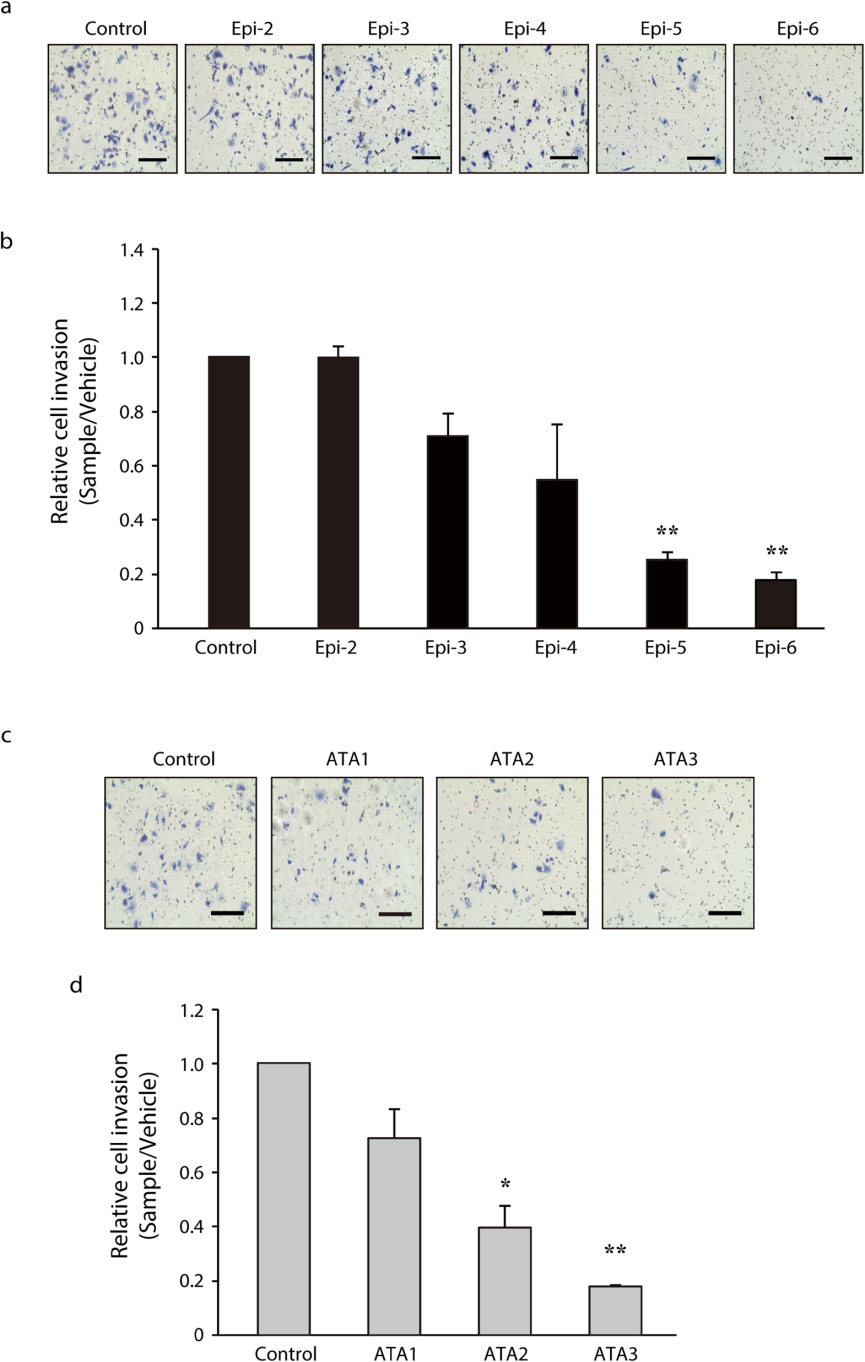
Effects of test compounds on the invasive activity of PC-3 prostate cancer cells. Test compounds (30 μmol/L) with the degree of oligomerization of epicatechin [pentamer (Epi-5), hexamer (Epi-6), ATA2 and ATA3] decreased invasive activities of PC-3 prostate cancer cells. The experimental procedure for preparation of test compounds was described in Biochemical methods of Supplementary Information. The invasive cells were fixed, stained and counted. Representative images of three independent experiments are shown in (**a**) and (**c**). Scale bar, 400 μm. The relative activities of cell invasion are shown in (**b**) and (**d**). The data are the means ± S.D. of three independent experiments. One-way ANOVA followed by Tukey’s multiple comparison test was used to compare means. *P < 0.05 or **P < 0.01.

As shown in Fig. 8, cinnamtannin A3 (Epi-5, **1**) and A4 (Epi-6, **2**) significantly decreased the number of cells invading through the Matrigel-coated membrane. This finding strongly suggests that epicatechin oligomers (Epi-5 or Epi-6) might suppress the invasiveness of PC-3 prostate cancer cells during metastasis. Suppression of invasion was clearly associated with the degree of oligomerization of epicatechin (pentamer and hexamer) at the 30 μmol/L dose level (Fig. 8). As *FABP5* is responsible for invasiveness of cancer cells during metastasis^26, 29^, suppression of the invasive activity by these epicatechin oligomers is, in part, attributable to down-regulation of *FABP5* gene expression by them (Fig. 7). We have tried to examine whether the epicatechin oligomer (Epi-5) suppresses key proteins for invasion such as MMPs (matrix metalloproteases). No significant suppression of these proteins was observed.

### Structural analyses of epicatechin or catechin oligomers

**Figure 9:**
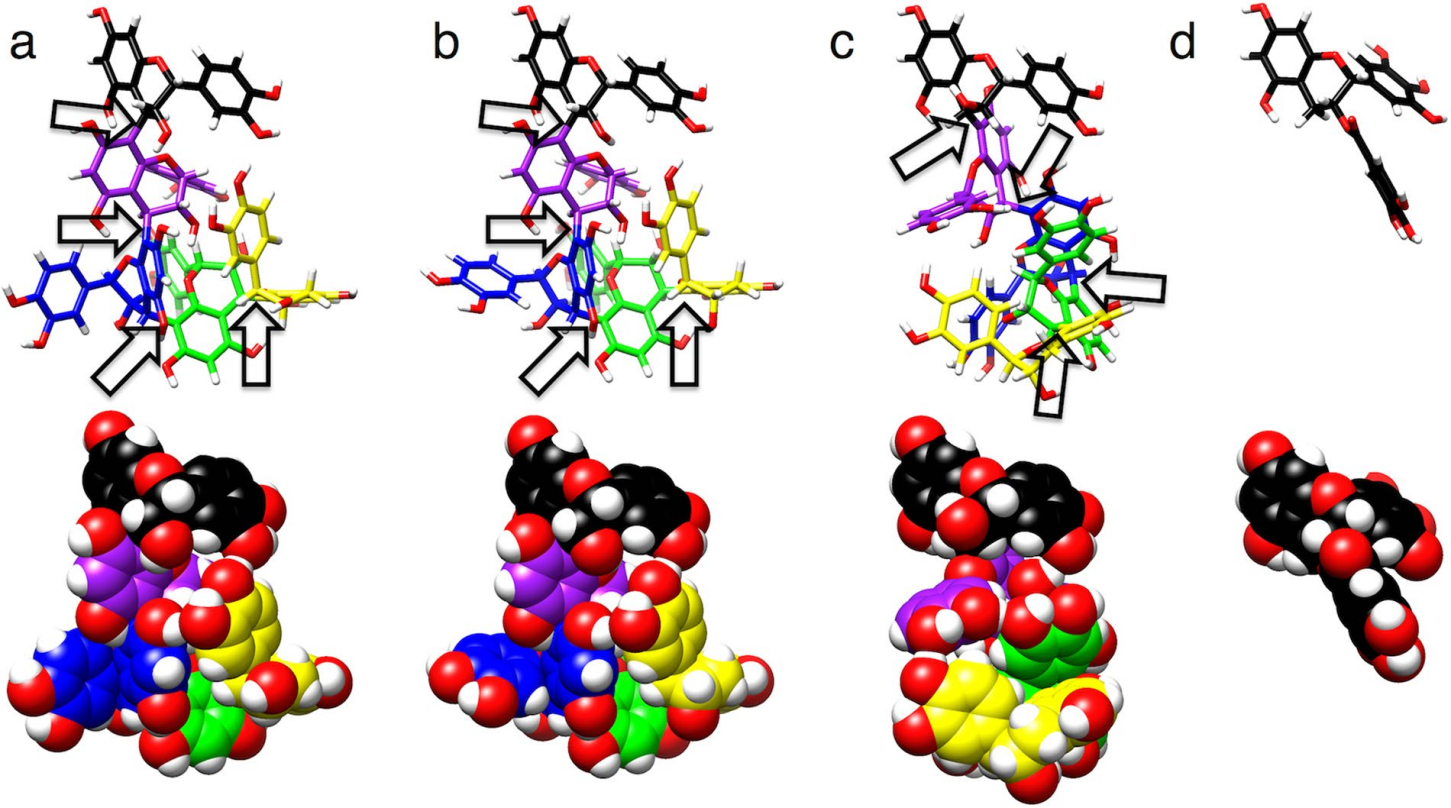
The lowest-energy structures of the pentamer and EGCG. The energy-minimized structures of Epi-5 (**a**), ATA3 (**b**) and Cat-5 (**c**) were computed with *ab-initio* quantum chemical simulation (the computational procedure is described in the main text). In the Epi-5 structure, the carbon atoms are colored in black, purple, blue, green and yellow from the first to fifth flavan unit. The oxygen and hydrogen atoms are red and white, respectively. The ATA3 and Cat-5 structures are presented using the same coloring scheme. The arrows indicate each of the internal flavan (C-4:C-8) bonds. The structure of EGCG after optimization with B3LYP/6-31G(d) is shown in (**d**). The space filing models are displayed below the stick models for viewing the exposed hydroxyl groups.

To investigate the anti-cancer activities of procyanidins based on the degree of oligomerization of epicatechin or catechin, three dimensional structures of the pentamers: epicatechin pentamer (Epi-5), arecatannin A3 (ATA3) and catechin pentamer (Cat-5) were calculated. The internal coordinates of the pentamers have rotational freedom. The conformation of the procyanidin dimer can rotate around the internal flavan bond between C-4 and adjacent C-8 atoms (C-4:C-8), which prescribes the relative direction of the first and second flavan units. It was reported that the catechin dimer may form two stable rotamers around the C-4:C-8 bond named as the compact (Co) and extended (Ex) rotamers^34^. The Co and Ex rotamer exhibits negative (~ −120°) and positive (~ + 60°) tortion angle around the C-4:C-8 bond, respectively. Herein, each pentamer of Epi-5, ATA3 and Cat-5 contains the four C-4:C-8 bonds and can adopt a total of 16 possible conformations. The stable conformation among them was then determined computationally. Sixteen initial structures were prepared with different rotamer states for Epi-5, ATA3 and Cat-5. These initial structures were optimized without restraint by density functional theory at the B3LYP level with the 6–31(d) basis set in the gas phase, which was conducted with Gaussian09 package^35^. Energies of the optimized structures were compared and the lowest-energy structure (LES) determined. The LESs of the pentamers and the energy-minimized structure of EGCG are shown in Fig. 9. Interestingly, the rotamer patterns of Epi-5 and ATA3 LESs are the same as Co-Co-Ex-Ex from the top of the C-4:C-8 bonds, whereas that of Cat-5 LES is Ex-Ex-Co-Ex. Furthermore, the three-dimensional distribution of the hydroxy groups is similar between Epi-5 and ATA3 LESs but not EGCG. This result suggests that Epi-5 and ATA3 might interact with a putative target molecule (receptor) via position-specific hydrogen bonds for regulating anti-cancer activities, as different mechanism from EGCG such as suppressing cell proliferation and cell invasion of PC-3 prostate cancer cells, while Cat-5 is not capable of such activity.

## Discussion

Although reports of the synthesis of catechin and/or epicatechin oligomers, especially those longer than a trimer are rather limited, in the present study syntheses of cinnamtannin A3 (epicatechin pentamer, **1**), catechin tetramer (**3**), pentamer (**4**), arecatannin A2 (**5**) and A3 (**6**) were achieved via Lewis acid mediated equimolar condensation. As to the synthesis of **2**, 1.7 eq. of nucleophile was necessary to obtain condensed product in good yield. Due to the establishment of synthetic method of various degrees of procyanidin oligomers, studies on their structural activity relationship and chemical probe syntheses would be possible. This synthetic study enabled us to evaluate the biological activities of procyanidins based on the degree of oligomerization of epicatechin or catechin. We investigated the anti-cancer activity of these compounds against human PC-3 prostate cancer cell lines. Among these compounds, cinnamtannin A4 (**2**), the pentamer of epicatechin derivatives, cinnamtannin A3 (**1**) and arecatannin A3 (**3**) showed significant activities for suppression of cell growth, invasion and *FABP5* gene expression. On the other hand, compounds with oligomeric states less than tetramers and the catechin pentamer did not show any activities for suppression of cell growth, invasion and *FABP5* gene expression. We found that the cytotoxic effects were clearly associated with the degree of oligomerization of epicatechin. As shown in Fig. 6, the levels of Cdc2, Cdc25C and Cyclin B1, critical regulators in the transition from G2 to S phase^36^, were significantly reduced in PC-3 cells treated with epicatechin oligomers, suggesting that the G2/M cell cycle arrest might be induced in PC-3 cells. Thus, the cell growth inhibitory activities of cinnamtannin A3 (Epi-5, **1**) and A4 (Epi-6, **2**) might be partly ascribed to their tendency to block the cell cycle partly at the G2 phase, but further mechanistic study should be needed to clarify suppression of cell proliferation by them. These compounds also suppressed the expression of cancer-promoting genes such as *FABP5* and invasion of cancer cells, suggesting that they represent promising anti-metastatic agents. Analyses of three-dimensional structures of epicatechin or catechin oligomers by theoretical calculations strongly suggested structural characteristic required for their anti-cancer activities. It would be interesting to examine whether Epi-5 and ATA3 would interact with a putative target molecule for regulating anti-cancer activities, while Cat-5 is not capable of such activity. Further mechanistic studies of their anti-cancer activities are now in progress.

Among green tea catechin, EGCG is the most abundant and anti-cancer constituent^37^. EGCG has been demonstrated to have cancer preventive activities in various cancer cells mediated by its antioxidant activity and its specific receptors to regulate gene expression and cellular signaling^38, 39^. However, we have preliminarily found that EGCG has less inhibitory activity for the cancer-promoting gene (*FABP5*) expression and less invasive activity compared with these epicatechin oligomers (unpublished results). As shown in Fig. 9, the structural comparison between EGCG and Epi-5 or ATA3 suggests that the pentamers might interact with a potent receptor other than that EGCG binds to.

Recently, it has been reported that procyanidins regulate vascular endothelial functions, suggesting implication for cardiovascular health^3, 40–43^. Although the regulatory mechanisms of vascular endothelial functions by the oligomeric procyanidins in red wine remains unclear, they are hypothesized to bind to a potent specific cell surface protein involved in mechanosensing or to act as receptor agonists, initiating mechanotransduction signaling in the vascular endothelial cells. It would be interesting to examine functional and structural differences of potential target protein/receptor for epicatechin oligomers between cancer cells and endothelial cells.

In conclusion, we demonstrated that epicatechin oligomers longer than trimers have significant anti-cancer activities, but not the catechin counterparts. Furthermore, the present study suggests that cinnamtannin A3 and A4 might be potential chemo-preventive agents for various cancers including prostate cancer. Further *in vivo* and mechanistic studies are needed to confirm these anti-cancer activities.

## Methods

### General

All melting points reported were uncorrected. ^1^H and ^13^C NMR spectra were measured with a Bruker DRX 500 FT-NMR spectrometer in CDCl_3_ or CD_3_OD at 500 and 125 MHz, respectively. Chemical shifts were relative to tetramethylsilane as an internal standard. The coupling constants are given in Hz. Mass spectra were obtained on Waters Xevo QTOF (MS-A) and Shimadzu LCMS-IT-TOF (MS-B) mass spectrometers. IR spectra were recorded with a JASCO FT-IR 480 Plus infrared spectrometer. Optical rotations were determined with a JASCO DIP-1000 polarimeter. HPLC data were recorded with GL Science GL-7400 (HPLC-A), Shimadzu HPLC LC-VP series (HPLC-B) and Waters ACQUITY UPLC (HPLC-C).

### Statistical analysis

GraphPad Prism 7.03 was used for statistical analyses. Data were expressed as the mean ± S.D. For multiple comparisons were calculated using one-way analysis of variance (ANOVA) with Tukey’s post-hoc test or two-way ANOVA with Dunnett’s post-hoc test. Statistical significance were indicated by the following indications: *P < 0.05, **P < 0.01.

### Experimental data

For supplementary Tables 1–5, see Supplementary Information pages 3–7. For the synthetic procedures of compounds **1**–**25**, see Supplementary Information pages 8–18. For biochemical methods, see Supplementary Information pages 19–21. For supplementary Figures 1 and 2, see Supplementary Information pages 23–24. For ^1^H and ^13^C NMR of synthesized compounds, HPLC data and MS spectra of compounds **1**–**6**, see Supplementary Information pages 26–69.

## Electronic supplementary material

Supplementary Information
