# Supplementary material for: Epicatechin oligomers longer than trimers have anti-cancer activities, but not the catechin counterparts
Source: Sci Rep. 2017 Aug 10;7:7791. doi: 10.1038/s41598-017-08059-x (PMC5552761; doi:10.1038/s41598-017-08059-x)
Supplement: Supplementary file 1 — Supplementary Information [file 41598_2017_8059_MOESM1_ESM.doc]

**Supplementary Information**

**Epicatechin oligomers longer than trimers have anti-cancer activities, but not the catechin counterparts**

Kohki Takanashi,^1†^Manato, Suda1^†^Kiriko Matsumoto,^2†^Chisato Ishihara,^3^ Kazuya, Toda,^2^ Koichiro Kawaguchi,^4^ Shogo Senga ^4^, Narumi Kobayashi ^3^, Mikihiro Ichikawa,^1^ Miyuki Katoh,^1^ Yasunao Hattori,^5^ Sei-ichi, Kawahara,^4^ Koji Umezawa,^3,6^ Hiroshi Fujii,^3,4,6^* and Hidefumi Makabe^1,4,6^*

^1^*Graduate School of Agriculture, Sciences of Functional Foods, Shinshu University, 8304 Minami-minowa Kami-ina, Nagano, 399-4598, Japan*

^2^*Department of Bioscience and Biotechnology, Faculty of Agriculture, Shinshu University, 8304 Minami-minowa Kami-ina, Nagano, 399-4598, Japan*

^3^*Department of Biomedical Engineering, Graduate School of Science and Technology, Shinshu University, 8304 Minami-minowa Kami-ina, Nagano, 399-4598, Japan*

^4^*Interdisciplinary Graduate School of Science and Technology, Shinshu University, 8304 Minami-minowa Kami-ina, Nagano, 399-4598, Japan*

^5^*Center for Instrumental Analysis, Kyoto Pharmaceutical University, Yamashina-ku, Kyoto 607-8412, Japan*

^6^*Department of Interdisciplinary Genome Sciences and Cell Metabolism, Institute for Biomedical Sciences, Interdisciplinary Cluster for Cutting Edge Research*, *Shinshu University, Minami-minowa, Kami-ina, Nagano*,

*399-4598, Japan*

*Corresponding author. Tel. +81 265 77 1626; fax +81 265 77 1626; e-mail: hfujii@shinshu-u.ac.jp

*Corresponding author. Tel. +81 265 77 1630; fax +81 265 77 1700; e-mail: [makabeh@shinshu-u.ac.jp](mailto:makabeh@shinshu-u.ac.jp)

**Table of contents:**

Supplementary Table 1-5 (page 3-7)

Synthetic Procedures (page 8-18)

Condition of the HPLC analysis (page 18)

Biochemical methods (page 19-22)

Supplementary Figure 1-2 (page 23-24)

Supplementary references (page 25)

Spectral data of synthetic compounds (page 26-69)

**Supplementary Table 1 | Equimolar condensation of the trimeric epicatechin electrophile with the dimeric nucleophile.**

| entry | eq. of Zn(OTf)_2_ | Time (h) | Yield of **10** (%) |
| --- | --- | --- | --- |
| 1 | 3.0 | 46 | 50 |
| 2 | 3.0 | 21 | 61 |
| 3 | 4.0 | 48 | 31 |
| 4 | 5.0 | 22 | 42 |

**Supplementary Table 2 | Equimolar condensation of the trimeric catechin nucleophile with the monomeric electrophile.**

| entry | Lewis acid (eq.) | Time (h) | Yield of **17** (%) |
| --- | --- | --- | --- |
| 1 | AgBF_4_ (1.0) | 4 | 26 |
| 2 | AgOTf (1.0) | 24 | 41 |
| 3 | AgOTf (1.5) | 24 | 53 |
| 4 | AgOTf (3.0) | 24 | 27 |

**Supplementary Table 3 | Equimolar condensation of the tetrameric catechin nucleophile with the monomeric electrophile.**

| entry | Lewis acid (eq.) | Time (h) | Yield of **18** (%) |
| --- | --- | --- | --- |
| 1 | Zn(OTf)_2_ (1.0) | 24 | 36 |
| 2 | Zn(OTf )_2_ (1.0) | 24 | 44 |
| 3 | AgOTf (3.0) | 72 | 50 |

**Supplementary Table 4 | Equimolar condensation of the epicatechin-catechin nucleophile 20 with the dimeric electrophile 9.**

| entry | Lewis acid (eq.) | Time (h) | Yield of **23** (%) |
| --- | --- | --- | --- |
| 1 | Yb(OTf)_3_ (2.0) | 46 | 49 |
| 2 | Yb(OTf)_3_ (3.0) | 73 | 53 |
| 3 | Yb(OTf)_3_ (5.0) | 25 | 64 |
| 4 | Yb(OTf)_3_ (6.0) | 24 | 26 |

**Supplementary Table 5 | Equimolar condensation of the epicatechin-epicetechin-catechin nucleophile 21 with the dimeric electrophile 9.**

| entry | Lewis acid (eq.) | Time (h) | Yield of **24** (%) |
| --- | --- | --- | --- |
| 1 | Zn(OTf)_2_ (5.0) | 21 | 51 |
| 2 | Yb(OTf)_3_ (4.0) | 43 | 52 |
| 3 | Yb(OTf)_3_ (5.0) | 44 | 59 |

**Synthetic procedures**

**3,3”-*O*-Diacetyl pentakis(5,7,3’,4’-tetra-*O*-benzyl)epicatechin (4β/8)_4_-pentamer (10).** To a solution of nucleophile **7** (19 mg, 0.01 mmol) and electrophile **9** (14 mg, 0.01 mmol) in CH_2_Cl_2_ (2 mL) under an argon atmosphere was added Zn(OTf)_2_ (11 mg, 0.03 mmol). After the resulting mixture had been stirred for 21 h at room temperature, the reaction was quenched with water. The mixture was extracted with EtOAc (20 mL x 2), and the combined organic layer was washed with brine, dried over MgSO_4_, filtered, and concentrated. The crude product was purified with silica gel column chromatography (hexane: EtOAc:CH_2_Cl_2_ = 4:1:2) to give **10** (20 mg, 61%) as a colourless oil. [α]_D_^19^ +128 (*c* 0.182, CHCl_3_), IR(film)ν_max_ cm^-1^: 3567, 3061, 3031, 2928, 1741, 1599, 1510, 1454, 1425, 1373, 1328, 1265, 1218, 1124, 1027, 735, 697; ^1^H NMR (CDCl_3_, 3:1 mixture of the rotational isomers, major isomer) δ = 7.50-6.80 (106H, m), 6.75 (1H, d, *J* = 7.5 Hz), 6.58 (1H, d, *J* = 7.5 Hz), 6.47 (1H, d, *J* = 7.5 Hz), 6.37 (1H, s), 6.36 (1H, d, *J* = 8.5 Hz), 6.13 (1H, d, *J* = 8.5 Hz), 5.97 (1H, d, *J* = 2.0 Hz), 5.88 (2H, s), 5.86 (1H, s), 5.74 (1H, s), 5.59 (1H, d, *J* = 2.0 Hz), 5.53 (1H, s), 5.48 (1H, s), 5.37 (1H, s), 5.20-4.20 (52H, m), 4.00 (1H, d, *J* = 6.5 Hz), 3.85 (1H, d, *J* = 6.5 Hz), 2.84-2.95 (2H, m), 1.74 (3H, s), 1.35 (1H, d, *J* = 6.5 Hz), 1.25 (3H, s), 1.18 (1H, d, *J* = 6.5 Hz), 1.15 (1H, m); ^13^C NMR (CDCl_3_, major isomer) δ = 169.2, 168.7, 157.9, 156.6, 156.3, 156.2, 156.0, 155.9, 155.8, 155.6, 155.3, 154.5, 153.1, 153.0, 152.6, 149.2, 148.9, 148.8, 148.6, 148.3, 148.2, 148.0, 147.6, 138.6, 138.3, 137.5-136.9, 133.0, 132.6, 132.5, 131.2, 128.6-126.0, 119.9, 119.4, 119.2, 119.0, 118.5, 114.9, 114.7, 114.5, 113.5, 113.3, 113.1, 113.0, 111.4, 111.1, 110.1, 106.5, 104.8, 104.6, 104.1, 101.2, 93.7, 93.4, 92.8, 91.2, 91.1, 77.8, 76.0, 75.7, 75.5, 74.8, 73.5, 72.4, 72.0, 71.3-68.9, 64.8, 35.0, 34.7, 28.4, 20.9, 20.3; HRESIMS calcd for C_219_H_186_O_32_Na [M+Na]^+^ 3350.2820, found 3350.2794.

**Pentakis(5,7,3’,4’-tetra-*O*-benzyl)epicatechin (4β/8)_4_-pentamer (12).** To a solution of **10** (40 mg, 0.012 mmol) in THF (3 mL) was added *n*-Bu_4_NOH (40%, 1.0 mL, 1.57 mmol). After the resulting mixture had been stirred for 180 h at room temperature, the reaction was quenched with water. The mixture was extracted with EtOAc (20 mL x 2), and the combined organic layer was washed with brine, dried over MgSO_4_, filtered, and concentrated. The residue was purified with preparative thin layer chromatography (hexane: EtOAc:CH_2_Cl_2_ = 4:1:2) to afford **12** (35 mg, 90%) as a colorless oil. [α]_D_^25^ +93.1 (*c* 1.40, CHCl_3_), {lit. [α]_D_^23^ +107.2 (*c* 0.43, CHCl_3_)}.^1^ IR (film) ν_max_ cm^-1^: 3567, 3061, 3031, 2928, 1599, 1510, 1454, 1425, 1373, 1328, 1265, 1218, 1124, 1027, 735, 697;^1^H NMR (CDCl_3_, 1:1 mixture of the rotational isomers) δ = 7.50-6.39 (116.5H, m), 6.39 (0.5H, s), 6.37 (0.5H, d, *J* = 8.2 Hz), 6.33 (0.5H, d, *J* = 8.2 Hz), 6.27 (0.5H, d, *J* = 2.0 Hz), 6.23 (0.5H, s), 6.14 (0.5H, dd, *J* = 8.2, 1.5 Hz), 6.07 (0.5H, d, *J* = 2.0 Hz), 6.05 (0.5H, *J* = 8.2 Hz), 6.03 (0.5H, dd, *J* = 8.2, 1.5 Hz), 5.91 (0.5H, s), 5.90 (0.5H, s), 5.88 (1H, s), 5.86 (0.5H, d, *J* = 2.0 Hz), 5.74 (1H, s), 5.63 (0.5H, d, *J* = 2.0 Hz), 5.52 (0.5H, s), 5.43 (0.5H, dd, *J* = 8.2, 1.5 Hz), 5.39 (0.5H, s), 5.37 (0.5H, s), 5.20-3.92 (47H, m), 3.89 (0.5H, d, *J* = 6.0 Hz), 3.66 (0.5H, brs.), 2.93-2.84 (2H, m), 1.79 (1H, d, *J* = 6.0 Hz), 1.60 (0.5H, d, *J* = 6.0 Hz), 1.51 (0.5H, d, *J* = 5.0 Hz), 1.43 (0.5H, d, *J* = 7.0 Hz), 1.37 (0.5H, d, *J* = 7.5 Hz), 1.34 (0.5H, d, *J* = 7.0 Hz), 1.24 (0.5H, d, *J* = 7.0 Hz), 1.14 (0.5H, *J* = 8.0 Hz), 1.05 (0.5H, *J* = 8.0 Hz); ^13^C NMR (CDCl_3_) δ = 158.3, 158.0, 157.3, 156.7, 156.6, 156.4, 156.3, 156.0, 155.5, 155.4, 155.2, 154.8, 153.2, 153.1, 152.8, 149.0-147.8, 138.8-137.0, 133.1, 132.7, 132.5, 131.3, 131.0, 128.8-126.1, 119.9, 119.7, 119.3, 118.7, 118.6, 118.5, 115.1-113.0, 111.3, 110.6, 110.1, 106.4, 105.0, 104.6, 101.2, 101.1, 94.3, 93.9, 93.4, 92.3, 92.0, 91.5, 90.4, 77.9, 76.2, 75.9, 75.7, 72.7, 72.6, 71.8, 71.4-69.2, 64.9, 37.0, 36.9, 36.3, 35.7, 35.1, 34.9, 28.5; HRESIMS calcd for C_215_H_182_O_30_Na [M+Na]^+^ 3266.2614, found 3266.2638.

**Cinnamtannin A3 (epicatechin pentamer) (1).** A solution of **12** (27 mg, 8.3 μmol) in THF/MeOH/H_2_O (20:1:1, 4.4 mL) was hydrogenated over Perlman’s catalyst (24 mg) for 5 h. The mixture was filtered and filtration residue was washed with MeOH (5 mL). The combined filtrates were evaporated, and the residue was taken up in distilled water (10 mL). The solution was filtered and lyophilized to give amorphous powder. This material was purified by HPLC to give **1** (10 mg, 86%) as a fluffy amorphous solid. [α]_D_^19^ +128 (*c* 0.230, MeOH), {lit. [α]+116 (*c* 8.3, MeOH)}.^2^ ^1^H NMR (CD_3_OD) δ = 7.25-6.71 (18H, m), 6.04-5.91 (7H, m), 5.49-4.61 (5H, m), 4.33 (1H, brs), 4.20-3.95 (4H, m), 3.00-2.72 (2H, m); ^13^C NMR (CD_3_OD) δ = 158.3, 158.0, 157.9, 157.2, 156.7, 156.6, 156.4, 155.0, 154.9, 154.6, 146.0, 145.9, 145.8, 145.7, 145.5, 145.4, 145.2, 132.6, 132.5, 132.3, 132.1, 119.4, 119.3, 119.1, 119.0, 116.2, 115.9, 115.3, 115.2, 107.7, 107.6, 107.5, 107.4, 102.4, 102.3, 101.8, 100.5, 97.6, 97.5, 97.4, 96.2, 96.0, 79.9, 79.7, 77.0, 73.5, 72.9, 72.8, 67.5, 66.8, 37.6, 37.4, 30.8, 29.8, 29.3; HRESIMS calcd for C_75_H_63_O_30_ [M+H]^+^, 1443.3412, found 1443.3359.

**3,3”-*O*-Diacetyl hexakis(5,7,3’,4’-tetra-*O*-benzyl)epicatechin (4β/8)_5_-hexamer (11).** To a solution of nucleophile **8** (46 mg, 17 μmol) and electrophile **9** (15 mg, 10 μmol) in CH_2_Cl_2_ (4 mL) under an argon atmosphere was added Zn(OTf)_2_ (18 mg, 50 μmol). After the resulting mixture had been stirred for 72 h at room temperature, the reaction was quenched with water. The mixture was extracted with EtOAc (20 mL x 2), and the combined organic layer was washed with brine, dried over MgSO_4_, filtered, and concentrated. The crude product was purified with silica gel column chromatography (hexane:EtOAc:CH_2_Cl_2_ = 4:1:2) to give **11** (26 mg, 64%) as a colourless oil. [α]_D_^19^ +117 (*c* 0.480, CHCl_3_), IR (film) ν_max_ cm^-1^: 3568, 3060, 3030, 2928, 1741, 1599, 1509, 1456, 1424, 1372, 1329, 1264, 1219, 1124, 1028, 735, 697; ^1^H NMR (CDCl_3_, 3:1 mixture of the rotational isomers, major isomer) δ = 7.50-6.75 (138H, m), 6.69 (1H, d, *J* = 7.5 Hz), 6.63 (1H, d, *J* = 7.5 Hz), 6.59 (1H, d, *J* = 7.5 Hz), 6.49 (1H, d, *J* = 7.5 Hz), 6.42-6.30 (5H, m), 6.20 (1H, d, *J* = 8.5 Hz), 6.14 (1H, d, *J* = 8.5 Hz), 5.97 (1H, d, *J* = 2.0 Hz), 5.92 (1H, s), 5.88 (2H, s), 5.84 (1H, s), 5.80 (1H, s), 5.60 (1H, d, *J* = 2.0 Hz), 5.53 (1H, s), 5.48 (1H, s), 5.37 (2H, s), 5.30-3.90 (50H, m), 2.96-2.85 (2H, m), 1.74 (3H, s), 1.33 (1H, d, *J* = 7.0 Hz), 1.30-1.20 (2H, m), 1.24 (3H, s), 1.13 (1H, brs); ^13^C NMR (CDCl_3_, major isomer) δ = 170.1, 169.2, 157.9, 156.6, 156.4, 156.1, 155.6, 155.2, 154.5, 153.1, 152.7, 149.2, 148.9, 148.8, 148.6, 148.3, 147.8, 147.6, 137.5-136.9, 133.0, 132.5, 131.2, 128.5-126.0, 119.9, 118.8, 118.5, 114.9, 114.8, 113.4, 113.2, 113.0, 111.2, 110.1, 109.6, 106.6, 105.0, 104.6, 104.3, 101.2, 93.8, 93.4, 92.4, 91.1, 90.6, 77.9-75.5, 71.3-68.9, 64.9, 35.2, 35.1, 29.7, 20.9, 20.3; HRESIMS calcd for C_262_H_222_O_38_Na [M+Na]^+^ 3998.5337, found 3998.5291.

**Hexakis(5,7,3’,4’-tetra-*O*-benzyl)epicatechin (4β/8)_5_-hexamer (13).** To a solution of **11** (20 mg, 5.0 μmol) in THF (2 mL) was added *n*-Bu_4_NOH (40%, 0.5 mL, 0.78 mmol). After the resulting mixture had been stirred for 180 h at room temperature, the reaction was quenched with water. The mixture was extracted with EtOAc (10 mL x 2), and the combined organic layer was washed with brine, dried over MgSO_4_, filtered, and concentrated. The residue was purified with preparative thin layer chromatography to afford **13** (17 mg, 90%) as a colorless oil. [α]_D_^19^ +121 (*c* 0.550, EtOAc), {lit. [α]_D_^23^+123 (*c* 12.2, EtOAc)}.^2^ IR (film) ν_max_ cm^-1^: 3565, 3060, 3031, 2928, 1597, 1508, 1455, 1422, 1374, 1327, 1264, 1219, 1123, 1026, 735, 697; ^1^H NMR (CDCl_3_, 1:1 mixture of the rotational isomers) δ = 7.45-6.63 (132H, m), 6.61 (0.5H, s), 6.51 (0.5H, s), 6.50 (0.5H, s), 6.45-6.38 (6.5H, m), 6.36 (0.5H, s), 6.32 (0.5H, s), 6,31 (0.5H, s), 6.23 (0.5H, s), 6.07 (0.5H, s), 6.04 (0.5H, d, *J* = 6.0 Hz), 5.96 (0.5H, s), 5.93 (0.5H, s), 5.91 (0.5H, s), 5.90 (0.5H, s), 5.87 (0.5H, d, *J* = 1.5 Hz), 5.84 (0.5H, s), 5.76 (0.5H, s), 5.75 (0.5H, s), 5.63 (0.5H, d, *J* = 1.5 Hz), 5.49 (0.5H, s), 5.47 (0.5H, s), 5.43 (0.5H, s), 5.41 (0.5H, s), 5.40 (0.5H, s), 5.37 (0.5H, s), 5.27 (0.5H, s), 5.21-3.88 (63.5H, m), 3.66 (0.5H, dd, *J* = 4.5, 1.5 Hz), 2.96-2.82 (2H, m), 1.81 (0.5H, d, *J* = 6.5 Hz), 1.60 (0.5H, d, *J* = 6.5 Hz), 1.41 (0.5H, d, *J* = 6.5 Hz), 1.35 (0.5H, d, *J* = 7.5 Hz), 1.32-1.20 (3H, m), 1.14 (0.5H, d, *J* = 7.0 Hz), 1.11 (0.5H, d, *J* = 6.5 Hz); ^13^C NMR (CDCl_3_) δ = 157.9, 157.3, 156.5, 156.3, 156.1, 156.0, 155.4, 154.8, 153.4, 153.2, 152.7, 149.0-147.6, 138.8-136.9, 134.0, 133.1, 132.6, 132.5, 131.3, 131.0, 128.6-126.1, 119.7, 119.4, 118.7, 118.5, 115.1-113.0, 111.3, 110.6, 110.1, 108.2, 107.2, 105.0, 104.6, 93.7, 77.9, 76.2, 75.9, 75.7, 72.5, 71.3-69.2, 65.3, 37.3, 36.9, 35.4, 35.1, 34.9, 28.6; HRESIMS calcd for C_258_H_218_O_36_Na [M+Na]^+^ 3914.5126, found 3914.5084.

**Cinnamtannin A4 (epicatechin hexamer) (2).** A solution of **13** (34 mg, 9.0 μmol) in THF/MeOH/H_2_O (20:1:1, 4.4 mL) was hydrogenated over Perlman’s catalyst (24 mg) for 2 h. The mixture was filtered and filtration residue was washed with MeOH (5 mL). The combined filtrates were evaporated, and the residue was taken up in distilled water (10 mL). The solution was filtered and lyophilized to give amorphous powder. This material was purified by HPLC to give **2** (3.2 mg, 21%) as a fluffy amorphous solid. [α]_D_^19^ +113 (*c* 0.550, MeOH), {lit. [α]_D_+123 (*c* 8.6, MeOH)}.^2^; ^1^H NMR (CD_3_OD) δ = 7.25-6.71 (22H, m), 6.12-5.90 (8H, m), 5.35-4.74 (8H, m), 4.35 (1H, brs), 4.20-3.95 (3H, m), 2.96 (1H, d, *J* = 15.8 Hz), 2.77 (1H, d, *J* = 15.8 Hz); ^13^C NMR (CD_3_OD) δ = 155.9, 155.3, 153.6, 144.6, 144.5, 144.1, 144.0, 131.2, 117.5, 114.8, 114.6, 113.8, 106.3, 100.8, 96.4, 75.6, 72.1, 71.4, 65.4, 36.2, 36.1, 29.4; HRESIMS calcd for C_90_H_75_O_36_ [M+H]^+^, 1731.4038, found 1731.4059.

**3-*O*-Acetyl-tetrakis(5,7,3’,4’-tetra-*O*-benzyl)catechin (4α/8)_3_-tetramer (17).** To a solution of nucleophile **14** (19 mg, 0.027 mmol) and electrophile **16** (52 mg, 0.027 mmol) in CH_2_Cl_2_ (4 mL) under an argon atmosphere was added AgOTf (10 mg, 0.040 mmol). After the resulting mixture had been stirred for 24 h at room temperature, the reaction was quenched with water. The mixture was extracted with Et_2_O, and the combined organic layer was washed with brine, dried over MgSO_4_, filtered, and concentrated. The crude product was purified with silica gel column chromatography (hexane:AcOEt:CH_2_Cl_2_ = 4:1:2) to give **17** (37 mg, 53%) as a colorless oil. [α]_D_^16^ −100 (*c* 1.00, CHCl_3_); HRFABMS calcd for C_174_H_148_O_25_Na [M+Na]^+^ 2660.0207, found 2660.0220.

**Tetrakis(5,7,3’,4’-tetra-*O*-benzyl)catechin (4α/8)_3_-tetramer (19).** To a solution of **17** (39 mg, 0.015 mmol) and in dioxane (2 mL) was added aqueous 10% KOH solution (0.5 mL). After the resulting mixture had been stirred for 11h under reflux, the reaction was cooled to room temperature and the reaction was quenched with 10% aqueous HCl solution. The mixture was extracted with EtOAc (5 mL x 3), and the organic layer was washed with water, aqueous saturated NaHCO_3_ solution, brine, dried over MgSO_4_, filtered, and concentrated. The crude product was purified with silica gel column chromatography (hexane:AcOEt:CH_2_Cl_2_ = 8:2:3) to give **19** (22 mg, 59%) as a colorless oil. [α]_D_^19^ −139 (*c* 0.850, CHCl_3_), {lit. [α]_D_^24^ −120 (*c* 1.50, CHCl_3_)}.^3^ ^1^H NMR (CDCl_3_, 3:2 mixture of rotational isomer, major isomer) δ = 7.45-6.20 (90H, m), 6.21 (1H, s), 6.18 (1H, d, *J* = 2.2 Hz), 6.09 (1H, d, *J* = 2.2 Hz), 6.07 (1H, s), 6.01 (1H, s), 5.75-3.90 (40H, m), 3.80-3.65 (2H, m), 3.62 (1H, d, *J* = 9.5 Hz), 3.09 (1H, d, *J* = 9.5 Hz), 3.08 (1H, dd, *J* = 17.0, 5.5 Hz), 2.81 (1H, d, *J* = 9.5 Hz), 2.34 (1H, dd, *J* = 16.0, 9.5 Hz), 1.40 (1H, s), 1.33 (1H, d, *J* = 3.2 Hz), 1.20 (1H, d, *J* = 3.5 Hz), 1.07 (1H, d, *J* = 4.0 Hz); ^13^C NMR (CDCl_3_, major isomer) δ = 157.9, 157.8, 156.6, 155.6, 155.4, 155.1, 154.9, 154.6, 153.7, 149.2, 149.1, 149.0, 148.9, 148.8, 148.6, 148.3, 138.9, 138.0, 137.6-137.0, 136.4, 135.7, 133.0, 132.6, 132.5, 131.8, 128.8-126.7, 121.1, 120.7, 120.5, 115.1, 114.9, 114.6, 114.5, 114.1, 113.7, 112.4, 112.3, 109.7, 109.2, 108.8, 102.3, 94.7, 93.7, 92.9, 92.0, 91.5, 81.9, 81.1, 80.9, 73.7, 73.2, 72.9, 71.6-69.8, 37.8, 37.7, 37.5, 33.5, 31.9, 29.7-28.4, 24.7, 22.7; HRESIMS calcd for C_172_H_146_O_24_Na [M+Na]^+^ 2618.0102, found 2618.0112.

**Catechin tetramer (3).** A solution of **19** (74 mg, 0.038 mmol) in THF/MeOH/H_2_O (20:1:1, 4.4 mL) was hydrogenated over Perlman’s catalyst (32 mg) for 3.5 h. The mixture was filtered and filtration residue was washed with MeOH (3 mL). The combined filtrates were evaporated, and the residue was taken up in distilled water (10 mL). The solution was filtered and lyophilized to give amorphous powder. This material was purified by HPLC to give **3** (15 mg, 81%) as a fluffy amorphous solid. [α]_D_^19^ −302 (*c* 0.950, MeOH), ^1^H NMR (CD_3_OD) δ = 7.31-6.40 (11H, m), 6.35-5.70 (7H, m), 4.90-3.60 (8H, m), 2.90-2.75 (2H, m), 2.55-2.50 (2H, m); ^13^C NMR (CD_3_OD) δ = 158.3, 157.9, 157.7, 157.2, 157.0, 156.3, 156.0, 155.6, 154.6, 146.8, 146.7, 145.9, 145.3, 145.0, 132.6, 132.4, 132.3, 132.1, 132.0, 121.6, 121.5, 121.0, 120.9, 120.6, 120.3, 120.1, 120.0, 116.9, 116.7, 116.1, 115.9, 115.5, 115.2, 109.6, 108.8, 108.2, 107.7, 107.4, 106.1, 100.8, 98.9, 97.6, 97.4, 96.4, 96.2, 96.0, 95.5, 85.9, 85.5, 84.0, 83.7, 83.4, 83.1, 82.9, 73.6, 73.4, 68.8, 68.5, 39.1, 38.6, 30.8, 30.2, 28.6; HRESIMS calcd for C_60_H_50_O_24_Na [M+H]^+^ 1155.2770, found 1155.2787.

**3-*O*-Acetyl-pentakis(5,7,3’,4’-tetra-*O*-benzyl)catechin (4α/8)_4_-pentamer (18).** To a solution of nucleophile **15** (35 mg, 0.014 mmol) and electrophile **16** (10 mg, 0.014 mmol) in CH_2_Cl_2_ (3 mL) under an argon atmosphere was added AgOTf (8 mg, 0.04 mmol). After the resulting mixture had been stirred for 72 h at room temperature, the reaction was quenched with water. The mixture was extracted with Et_2_O, and the combined organic layer was washed with brine, dried over MgSO_4_, filtered, and concentrated. The crude product was purified with silica gel column chromatography (hexane:AcOEt:CH_2_Cl_2_ = 3:1:1) to give **18** (22 mg, 50%) as a colorless oil. [α]_D_^16^ −100 (*c* 1.00, CDCl_3_), HRESIMS calcd for C_217_H_184_O_31_Na [M+Na]^+^ 3308.2719, found 3308.2744.

**Pentakis(5,7,3’,4’-tetra-*O*-benzyl)catechin (4α/8)_4_-pentamer (20).** To a solution of **18** (52 mg, 0.016 mmol) and in CH_2_Cl_2_ (3 mL) was added DMAP (1 mg) and acetic anhydride (0.013 mL, 0.16 mmol) at 0 ^o^C. After the resulting mixture had been stirred for 19 h at room temperature, the reaction was quenched with water. The mixture was extracted with EtOAc (10 mL x 3), and the organic layer was washed with water, saturated aqueous NaHCO_3_ solution, brine, dried over MgSO_4_, filtered, and concentrated. The crude product was dissolved in CH_2_Cl_2_ (4 mL) and the whole was cooled to −78 ^o^C. DIBALH (1.04 mol in hexane, 0.44 mL, 0.46 mmol) was added to the mixture at this temperature. After the resulting mixture had been stirred for 17h, the reaction was quenched with MeOH (5 mL). The mixture was filtered through Celite pad and the filtrate was concentrated. The resulting crude products was purified with silica gel thin layer chromatography (hexane:AcOEt:CH_2_Cl_2_ = 3:1:2) to afford **20** (37 mg, 77%, 2 steps). [α]_D_^19^ −133 (*c* 1.85, CHCl_3_), ^1^H NMR (CDCl_3_, 9:1 rotational isomer, major isomer) δ = 7.43-7.08 (110H, m), 7.04-6.74 (12H, m), 6.71-6.60 (8H, m), 6.56-6.48 (2H, m), 6.36-6.32 (1H, m), 6.28-6.23 (1H, m), 6.21 (1H, d, *J* = 3.5 Hz), 6.17 (1H, d, *J* = 1.5 Hz), 6.09 (1H, d, *J* = 2.0 Hz), 6.06-5.98 (3H, m), 5.21-4.81 (15H, m), 4.76-4.33 (8H, m), 4.31-4.25 (2H, m), 4.20-4.07 (3H, m), 4.04-3.96 (1H, m), 3.86-3.81 (1H, m), 3.76-3.65 (2H, m), 3.62 (1H, d, *J* = 8.5 Hz), 3.12-3.02 (1H, m), 2.96-2.92 (1H, m), 2.84-2.75 (1H, m), 2.34 (1H, dd, *J* = 16.5, 10.0 Hz), 1.36 (2H, d, *J* = 2.0 Hz), 1.19 (1H, d, *J* = 3.0 Hz), 1.17 (1H, d, *J* = 3.5 Hz), 1.13 (1H, d, *J* = 4.5 Hz); ^13^C NMR (CDCl_3_, major isomer) δ = 157.9, 157.8, 156.5, 155.9, 155.5, 155.3, 155.2, 155.1, 155.0, 154.8, 154.8, 154.7, 154.5, 153.6, 149.1, 149.0, 148.9, 148.8, 148.7, 148.6, 148.5, 148.2, 147.7, 128.7-126.5, 121.1, 121.0, 120.9, 120.5, 120.4, 114.9, 114.8, 114.5, 114.0, 113.6, 112.9, 112.8, 112.2, 112.1, 109.5, 109.4, 109.3, 109.1, 108.7, 102.2, 94.6, 93.6, 92.6, 92.2, 91.9, 91.2, 81.7, 81.1, 80.9, 80.8, 80.1, 73.2, 73.0, 72.9, 71.4-69.7, 68.4, 60.3, 37.9, 37.7, 37.5, 29.6, 28.3; HRESIMS calcd for C_215_H_182_O_30_Na [M+Na]^+^ 3266.2614, found 3266.2633

**Catechin pentamer (4).** A solution of **20** (37 mg, 0.011 mmol) in THF/MeOH/H_2_O (20:1:1, 2.2 mL) was hydrogenated over Perlman’s catalyst (33 mg) for 4.5 h. The mixture was filtered and filtration residue was washed with MeOH (3 mL). The combined filtrates were evaporated, and the residue was taken up in distilled water (10 mL). The solution was filtered and lyophilized to give amorphous powder. This material was purified by HPLC to give **4** (14 mg, 78%) as a fluffy amorphous solid. [α]_D_^19^ −188 (*c* 0.185, MeOH), ^1^H NMR (CD_3_OD) δ = 7.10-6.40 (17H, m), 6.30-5.75 (8H, m), 4.70-3.50 (10H, m), 2.90-2.45 (6H, m); ^13^C NMR (CD_3_OD) δ = 158.6, 157.8, 157.6, 157.1, 156.9, 155.9, 155.6, 154.9, 146.2, 146.1, 145.8, 145.5, 145.3, 132.6, 132.2, 131.8, 121.0, 120.6, 120.3, 120.0, 119.8, 116.4, 116.2, 116.1, 116.0, 115.9, 115.5, 115.2, 108.15, 107.17, 100.8, 97.4, 97.3, 96.8, 96.2, 96.0, 95.4, 83.9, 82.9, 82.4, 73.7, 68.9, 68.8, 68.5, 38.6, 30.8, 28.8, 28.5; HRESIMS calcd for C_75_H_63_O_30_ [M+H]^+^ 1443.3404, found 1443.3450.

**3,3”-*O*-Diacetyl tetrakis(5,7,3’4’-tetra-*O*-benzyl)-(4β/8)_3_-epicatechin-epicatechin-epicatechin-catechin (23).** To a solution of nucleophile **21** (33 mg, 0.025 mmol) and electrophile **9** (37 mg, 0.025 mmol) in CH_2_Cl_2_ (5.0 mL) was added Zn(OTf)_2_ (46 mg, 0.13 mmol). After the resulting mixture had been stirred for 25 h, the reaction was quenched with water. The mixture was extracted with EtOAc (10 mL x 3), and the organic layer was washed with water, brine, dried over MgSO_4_, filtered, and concentrated. The crude product was purified with silica gel column chromatography (hexane:AcOEt:CH_2_Cl_2_ = 5:1:2) to give **23** (43 mg, 64%) as a colorless oil. [α]_D_^20^ +112 (*c* 0.650 CHCl_3_); IR (film) ν_max_ cm^-1^: 3567, 3063, 3031, 2929, 1739, 1599, 1511, 1454, 1428, 1373, 1265, 1218, 1122, 1027, 737, 697; ^1^H NMR (CDCl_3_, 4:1 mixture of the rotational isomers, major isomer) δ = 7.48-6.63 (97H, m), 6.36 (1H, s), 5.97 (1H, d, *J* = 8.0 Hz), 5.94 (1H, s), 5.87 (1H, s), 5.77 (1H, s), 5.59 (1H, d, *J* = 2.0 Hz), 5.52 (1H, s), 5.48 (1H, s), 5.20-4.21 (34H, m), 3.86 (1H, d, *J* = 6.5 Hz), 3.30 (1H, m), 2.90 (1H, dd, *J* = 16.0, 9.0 Hz), 1.73 (3H, s), 1.48 (1H, d, *J* = 3.5 Hz), 1.32 (3H, s), 1.25 (1H, brs); ^13^C NMR (CDCl_3_, major isomer) δ = 169.1, 168.8, 157.9, 156.5, 156.4, 156.0, 155.8, 155.6, 155.4, 155.3, 155.2, 154.5, 153.0, 152.8, 149.2, 148.9, 148.8, 148,7, 148.3, 147.8, 147.6, 138.6, 138.0, 137.6-136.9, 132.5, 131.4, 131.2, 128.6-125.9, 120.1, 119.9, 114.5, 113.5, 113.3, 111.5, 110.2, 109.9, 109.6, 106.4, 104.6, 104.5, 100.4, 93.6, 93.4, 91.2, 90.8, 90.5, 81.2, 76.0, 75.5, 74.7, 73.5, 72.4, 71.7, 71.3-71.0, 70.4, 69.8, 69.6, 69.5, 69.2, 68.8, 68.1, 34.6, 32.9, 27.4, 20.8, 20.4; HRESIMS calcd for C_176_H_150_O_26_Na [M+Na]^+^ 2702.0308, found 2702.0332.

**Tetrakis(5,7,3’4’-tetra-*O*-benzyl)-(4β/8)_3_-epicatechin-epicatechin-epicatechin-catechin (25).** To a solution of **23** (67 mg, 0.025 mmol) in THF (4.0 mL) was added *n*-Bu_4_NOH (40%, 1.3 mL, 2.0 mmol). After being stirred for 140 h, the mixture was concentrated *in vacuo* and the resulting mixture was extracted with EtOAC (10 mL x 2). The organic layer was washed with H_2_O, brine, dried over anhydrous MgSO_4_, filtered, and concentrated. The residue was purified with preparative thin layer chromatography (hexane:EtOAc:CH_2_Cl_2_ = 4:1:2) to give **25** (58 mg, 89%) as a pale yellow oil. [α]_D_^19^ +130 (*c* 0.500, CHCl_3_); IR (film) ν_max_ cm^-1^: 3567, 3031, 2906, 1598, 1508, 1454, 1420, 1376, 1264, 1216, 1119, 1026, 735, 696; ^1^H NMR (CDCl_3_, major isomer) δ = 7.53-6.62 (92H, m), 6.40 (1H, d, *J* = 8.5 Hz), 6.34 (1H, s), 6.26 (1H, d, *J* = 2.0 Hz), 6.19 (1H, s), 6.06 (1H, d, *J* = 2.0 Hz), 5.91 (1H, s), 5.72 (1H, d, *J* = 8.5 Hz), 5.51 (1H, s), 5.12 (1H, brs), 5.21-4.38 (36H, m), 4.39 (1H, d, *J* = 12.0 Hz), 3.99 (1H, d, *J* = 6.5 Hz), 3.56 (1H, brs), 3.43 (1H, m), 3.03 (1H, dd, *J* = 16.0, 5.0 Hz), 2.61 (1H, dd, *J* = 16.0, 9.0 Hz), 1.68 (1H, d, *J* = 6.0 Hz), 1.55 (1H, brs), 1.27 (1H, d, *J* = 6.0 Hz); ^13^C NMR (CDCl_3_, major isomer) δ = 158.3, 158.0, 157.3, 156.7, 156.6, 156.3, 156.1, 156.0, 155.6-155.1, 154.7, 153.2, 152.8, 149,2, 148.9-147.6, 138.8, 137.6-136.9, 132.9, 132.3, 131.3, 128.6-125.9, 120.1, 119.9, 119.7, 119.2, 119.0, 118.9, 118.5, 115.0, 114.9, 114.6, 114.5, 114.0, 113.6-112.8, 111.0, 110.8, 110.7, 110.1, 109.9, 106.2, 105.3, 104.7, 104.4, 104.3, 101.9, 94.3, 93.9, 93.8, 93.4, 92.5, 91.9, 91.8, 91.1, 90.4, 81.2, 77.2-76.1, 75.8-75.6, 72.7, 71.3-69.0, 68.1, 36.3, 35.6, 35.4, 35.1, 34.9, 27.4; HRESIMS calcd for C_172_H_146_O_24_Na [M+Na]^+^ 2618.0096, found 2618.0121.

**Arecatannin A2 (5).** A solution of **25** (38 mg, 15 μmol) in THF/MeOH/H_2_O (20:1:1, 4.4 mL) was hydrogenated over Perlman’s catalyst (27 mg) for 6 h. The mixture was filtered and filtration residue was washed with MeOH (5.0 mL). The combined filtrates were evaporated, and the residue was taken up in distilled water (10 mL). The solution was filtered and lyophilized to give amorphous powder. This material was purified by HPLC to give **5** (11 mg, 81%) as a fluffy amorphous solid. [α]_D_^19^ +76.2 (*c* 0.295, MeOH), {lit. [α]_D_^25^+73.3 (*c* 0.10, MeOH)}.^1^ IR (KBr) ν_max_cm^−1^: 3291, 1608, 1521, 1437, 1109, 682; ^1^H NMR (CD_3_OD) δ = 7.10-6.67 (12H, m), 6.05-5.91 (5H, m), 5.30 (1H, brs), 5.25 (1H, brs), 5.08 (1H, brs), 4.95-4.70 (4H, m), 4.17-4.03 (4H, brs), 2.70-2.60 (2H, brs); ^13^C NMR (CD_3_OD) δ = 158.5-155.8, 155.0-154.8, 153.9, 146.2-145.3, 132.7, 119.3-119.1, 116.2-116.0, 115.2, 115.1, 114.5, 108.2, 97.6-97.3, 96.6, 96.3, 82.0, 77.2, 77.1, 73.5, 72.9, 72.2, 68.4, 37.6-37.1, 26.8; HRESIMS calcd for C_60_H_49_O_24_ [M−H]^−^ 1153.2614, found 1153.2627.

**3,3”-*O*-Diacetyl pentakis(5,7,3’,4’-tetra-*O*-benzyl)-(4β/8)_4_-epicatechin-epicatechin-epicatechin-epicatechin-catechin (24).** To a solution of nucleophile **22** (23 mg, 0.012 mmol) and electrophile **9** (17 mg, 0.012 mmol) in CH_2_Cl_2_ (5.0 mL) was added Yb(OTf)_3_ (37 mg, 0.059 mmol). After the resulting mixture had been stirred for 44 h, the reaction was quenched with water. The mixture was extracted with EtOAc (10 mL x 3), and the organic layer was washed with water, brine, dried over MgSO_4_, filtered, and concentrated. The crude product was purified with silica gel column chromatography (hexane:AcOEt:CH_2_Cl_2_ = 5:1:2) to give **24** (23 mg, 59%) as a colorless oil. [α]_D_^20^ +134 (*c* 0.73 CHCl_3_); IR (film) ν_max_ cm^-1^: 3566, 3030, 2932, 1741, 1599, 1509, 1454, 1425, 1373, 1264, 1217, 1123, 1027, 736, 696; ^1^H NMR (CDCl_3_, 3:2 mixture of the rotational isomers, major isomer) δ = 7.49-6.63 (120H, m), 6.50 (1H, d, *J* = 8.0 Hz), 6.37 (1H, s), 6.35 (1H, s), 5.97 (1H, s), 5.88 (1H, s), 5.87 (1H, s), 5.76 (1H, s), 5.59 (1H, d, *J* = 1.5 Hz), 5.53 (1H, s), 5.46 (1H, s), 5.35 (1H, s), 5.19 (1H, s), 5.15-4.23 (40H, m), 3.91 (1H, d, *J* = 7.0 Hz), 3.84 (1H, d, *J* = 7.5 Hz), 3.43 (1H, m), 2.95 (1H, dd, *J* = 16.0, 5.0 Hz), 2.58 (1H, dd, *J* = 16.5, 8.5 Hz), 1.74 (3H, s), 1.54 (1H, d, *J* = 3.0 Hz), 1.33 (1H, d, *J* = 7.0 Hz), 1.25 (3H, s), 1.08 (1H, d, *J* = 6.0 Hz); ^13^C NMR (CDCl_3_, major isomer) δ = 169.1, 168.6, 157.9, 156.6, 156.1, 156.0, 155.9, 155.8, 155.6, 155.4, 155.2, 154.5, 153.5, 153.1, 152.7, 149.2, 148.9, 148.8, 148,6, 148.3, 147.9, 147.6, 138.6, 138.3, 137.5-136.9, 133.0, 132.7, 132.5, 131.3, 131.2, 128.6-125.9, 120.0, 119.9, 119.4, 119.2, 119.0, 114.9, 114.5, 114.5, 113.4, 113.3, 111.4, 110.9, 110.1, 109.8, 109.6, 106.5, 104.8, 104.6, 104.3, 102.0, 93.6, 93.3, 92.2, 91.2, 91.0, 90.4, 81.1, 76.0, 75.8, 75.5, 74.7, 73.5, 72.4, 72.0, 71.3-71.0, 70.8, 70.4, 69.9, 69.7, 69.3, 69.2, 68.9, 68.4, 68.1, 35.1, 34.9, 32.9, 27.3, 20.8, 20.3, 20.2 ; HRESIMS calcd for C_219_H_186_O_32_Na [M+Na]^+^ 3350.2820, found 3350.2800.

**Pentakis(5,7,3’,4’-tetra-*O*-benzyl)-(4β/8)_4_-epicatechin-epicatechin-epicatechin-epicatechin-catechin (26).** To a solution of **24** (72 mg, 0.022 mmol) in THF (4.0 mL) was added *n*-Bu_4_NOH (40% in H_2_O, 1.4 mL, 2.2 mmol). After being stirred for 179 h, the mixture was concentrated *in vacuo* and the resulting mixture was extracted with EtOAc (10 mL x 2). The organic layer was washed with H_2_O, brine, dried over anhydrous MgSO_4_, filtered and concentrated. The residue was purified with preparative TLC (hexane:AcOEt:CH_2_Cl_2_ = 4:1:2) to give **26** (63 mg, 90%) as a pale yellow oil. [α]_D_^19^ +114 (*c* 0. 630, CHCl_3_); IR(film)ν_max_ cm^-1^: 3568, 3030, 2929, 1598, 1509, 1454, 1422, 1377, 1264, 1216, 1120, 1026, 735, 696; ^1^H NMR (CDCl_3_, 8:1 rotational isomer, major isomer) δ = 7.52-6.61 (120H, m), 6.51 (1H, d, *J* = 7.5 Hz), 6.43-6.31 (2H, m), 6.28 (1H, d, *J* = 1.5 Hz), 6.23 (1H, s), 6.07 (1H, s), 6.06 (1H, s), 5.95 (1H, s), 5.93 (1H, dd, *J* = 5.5 Hz), 5.89 (1H, s), 5.76 (1H, s), 5.73 (1H, s), 5.63 (1H, d, *J* = 2.0 Hz), 5.46 (1H, s), 5.41 (1H, d, *J* = 4.5 Hz), 5.24-3.87 (39H, m), 3.66 (1H, d, *J* = 4.0 Hz), 3.47-3.37 (1H, m), 2.96 (1H, dd, *J* = 16.5, 5.5 Hz), 2.63-2.55 (1H, m), 1.81 (1H, d, *J* = 6.0 Hz), 1.53 (1H, d, *J* = 3.5 Hz), 1.40 (1H, d, *J* = 6.0 Hz), 1.07 (1H, d, *J* = 7.0 Hz), ^13^C NMR (CDCl_3_, major isomer) δ = 158.2, 158.0, 157.3, 156.6, 156.5, 156.4, 156.2, 156.1, 156.0, 155.9, 155.6, 155.5, 155.3, 155.1, 154.7, 153.2, 153.1, 152.9, 152.8, 149.2, 148.9-147.6, 138.8, 138.1, 138.0, 137.9, 137.6-136.9, 132.9, 132.5, 132.3, 131.3, 128.5-126.0, 120.0, 119.9, 119.7, 119.3, 118.9, 118.7, 118.6, 118.4, 114.9, 114.5, 113.9, 113.6-112.7, 110.9, 110.6, 110.5, 110.3, 110.0, 109.9, 106.3, 105.3, 105.2, 104.7, 104.4, 102.1, 102.0, 94.3, 93.8, 93.3, 92.5, 92.2, 91.9, 91.3, 90.3, 81.2, 76.1, 75.8, 75.6, 72.5, 71.8-69.3, 68.1, 37.0, 36.3, 35.6, 35.41, 35.3, 34.9, 29.6, 27.3; HRESIMS calcd for C_215_H_182_O_30_Na [M+Na]^+^ 3266.2614, found 3266.2580.

**Arecatannin A3 (6).** A solution of **26** (18 mg, 5.5 μmol) in THF/MeOH/H_2_O (20:1:1, 4.4 mL) was hydrogenated over Perlman’s catalyst (13 mg) for 6 h. The mixture was filtered and filtration residue was washed with MeOH (1.0 mL). The combined filtrates were evaporated, and the residue was taken up in distilled water (10 mL). The solution was filtered and lyophilized to give amorphous powder. This material was purified by HPLC to give **6** (7 mg, 88%) as a fluffy amorphous solid. [α]_D_^19^ +120 (*c* 0.650, acetone), ^1^H NMR (CD_3_OD) δ = 7.14 -6.72 (15H, m), 6.09-5.87 (6H, m), 5.36-5.26 (3H, m), 5.12 (1H, brs), 4.97-4.63 (4H, m), 4.22-3.93 (6H, m), 2.75-2.63 (2H, brs); ^13^C NMR (CD_3_OD) δ = 158.5-155.9, 155.0, 153.9, 146.2-145.4, 132.7, 119.4-119.0, 116.2-115.9, 115.3-115.0, 114.5, 107.8, 107.7, 107.2, 107.1, 102.3, 101.5, 100.8, 97.7-97.2, 96.6, 96.4, 96.3, 95.9, 82.1, 79.9, 77.2-77.0, 73.5, 72.9, 72.8, 68.4, 37.7-37.1, 30.8, 29.1, 26.8; HRESIMS calcd for C_75_H_61_O_30_ [M−H]^−^ 1441.3248, found 1441.3245.

**Condition of the HPLC analysis.**

Column A：YMC-Triart-Phenyl φ4.6 μm × 250 mm, Flow: 1.0 mL/min, column oven: 35^o^C, mobile phase: 0.2%AcOH/Acetonitrile: 0 min 100/0, 10 min 93/7, 25 min 80/20, 35min 0/100, monitored wave length 280 nm. Column B: InertSustain C18, GL Scienceφ 4.6 μm × 250 mm, Flow: 1.0 ml/min, column oven: 35^o^C, mobile phase: 0.2% AcOH/Acetonitrile: 0 min 100/0, 10 min 93/7, 25 min 80/20, 35 min 0/100, monitored wave length 280 nm.

**Biochemical methods**

**Cell lines, cell culture and reagents.**

Human prostate cancer cell, PC-3, was purchased from the Health Science Research Resources Bank. The cells were maintained in monolayer culture at 37 °C and 5% CO_2_ in RPMI-1640 (SIGMA, R8755) supplemented with 10% charcoal-stripped fetal bovine serum (Biological Industries, No. 04-201-1), 1% antibiotic-antimycotic mixed stock solution (Nacalai Tesque, No. 09366-44) ^6, 7^. The cells were treated with various concentrations of epigallocatechine-3-gallate (EGCG), arecatannin A1 (ATA1), arecatannin A2 (ATA2), arecatannin A3 (ATA3), catechin dimer (Cat-2), catechin trimer (cat-3), catechin tetramer (Cat-4), catechin pentamer (Cat-5), epicatechin dimer (Epi-2), epicatechin trimer (Epi-3), epicatechin tetramer (Epi-4), epicatechin pentamer (Epi-5) and epicatechin hexamer (Epi-6) for 48 h. EGCG was generously gifted from Prof. Dr. Toshiyuki Kan in University of Shizuoka. The experimental procedure for preparation of test compounds was described in the following section.

**Preparation of test compounds.**

The test compounds, various procyanidins, were dissolved in 60% (v/v) ethanol and prepared for the concentrated solution (10-fold concentration) with the midium (RPMI 1640, Hyclone, Thermo Scientific) containing 10% charcoal-stripped FBS (Hyclone, Thermo Scientific) and antibiotic/antimycotic solution (Nacalai Tesque). The concentrated solution was filtrated by using a 0.2-um filter (Sartorius Stedim Biotech, Goettingen, Germany). In the experiments, the concentrated solution was added to the samples to a final concentration as indicated. Final concentration of ethanol of each sample including the control is 6% v/v. The medium used during treatment periods is the same as the growth medium (RPMI 1640, Hyclone, Thermo Scientific) supplemented with charcoal-stripped 10% FBS (Hyclone, Thermo Scientific) and antibiotic/antimycotic solution (Nacalai Tesque).

**Cell count.**

Cells were plated in 12-well plates and grew to reach 50% confluent. The cells were treated with the indicated concentrations of EGCG, ATA1, ATA2, ATA3, Cat-4, Cat-5, Epi-4, Epi-5 or Epi-6 for 48 h. Each treatment and time point had three plates. The cells treated with the above test compounds were washed with PBS and fixed with 4% formaldehyde for 10 min. Subsequently, the cells were washed with PBS and incubated with 0.5 µg/ml Hoechst33342 (Nacalai Tesque) diluted in DMSO (Nacalai Tesque) for 5 min^4^. Hoechst33342 is required for automated single cell detection and cell counting by High-Content Analysis. After the reaction, the cells were washed with PBS, and viewed and counted these nuclei using a Cellinsight NXT, a cellular imaging and analysis system (Thermo Scientific). And the results were analyzed by HCS Studio Cell Analysis Software (Thermo Scientific) ^7^.

**qPCR (quantitative real-time PCR).**

Cells were plated in 6-well plates and grew to reach 50% confluent. The cells were treated with the indicated concentrations of 50 μmol/L test compounds (ATA1, ATA2, ATA3, Cat-2, Cat-3, Cat-4, Cat-5, Epi-4, Epi-5 or Epi-6) for 48 h. Total RNA of these cells was extracted using the Plant RNA Purification Reagent (Invitrogen No. 12322-012), and 1 μg of total RNA was reverse-transcribed into cDNA using the ReverTra Ace qPCR RT Master Mix (Toyobo No. FSQ-301). qPCR analyses were performed with the StepOne Real-Time PCR system (Applied Biosystems) using THUNDERBIRD® SYBR® qPCR Mix (Toyobo No. QPS-201) ^6, 7^. The sequences of the FABP5, Cdc2, Cdc25C, Cyclin B1 and GAPDH primers for qPCR are as follows.

FABP5 (Forward): 5’-GCTGATGGCAGAAAAACTCAGA-3’, (Reverse): 5’-CCTGATGCTGAACCAATGCA-3’

Cdc2 (Forward): 5’-CCAAATATAGTCAGTCTTCAGGATGT-3’, (Reverse): 5’-CCATGGAAAGAAACTCAAAGATGA-3’

Cdc25C (Forward): 5’-TCCTTAAAGGCGGCTACAGA-3’, (Reverse): 5’-GCCAGTGGCTGGAATGTTAT-3’

Cyclin B1 (Forward): 5’-AGCCAGAACCTGAGCCAGAA-3’, (Reverse): 5’-TTTCCATTGGGCTTGGAGAG-3’

GAPDH (Forward): 5’-CAGCCTCAAGATCATCAGCA-3’, (Reverse): 5’-GGTGCTAAGCAGTTGGTGGT-3’

**Western blot analysis.**

Cells were plated in 60 mm cell culture dishes and grew to reach 50% confluent. The cells were treated with the indicated concentrations of 50 μmol/L Epi-2, Epi-3, Epi-4, Epi-5 or Epi-6 for 48 h. These cells were lysed for protein extraction using Lysis buffer with protease inhibitor cocktail (Nacalai Tesque No. 25955-11). After protein concentrations were determined by Bradford assay, 50 μg of protein per sample was fractionated by SDS/PAGE. Western blot analysis was carried out using the appropriate antibodies and chemiluminescent substrate (Thermo Scientific # NCI3106)^6, 7^. Signals were detected with the Image Quant LAS4000 Mini (GE Healthcare Biosciences). The antibodies were used as follows. FABP5 (D1A7T) Rabbit mAb #39926 (Cell Signaling Technology). Cdc25C (5H9) Rabbit mAb #4688S (Cell Signaling Technology). Cdc2 (POH1) Mouse mAb #9116S (Cell Signaling Technology). Cyclin B1 Antibody rabbit #4138S (Cell Signaling Technology). GAPDH antibody (FL-335): sc-25778 (Santa Cruz Biotechnology).

**Cell cycle analysis.**

Cells were plated in 90 mm cell culture dishes and grew to reach 50% confluent. The cells were treated with 200 μmol/L Epi-5. After 48 h cells were harvested with Trypsin-EDTA (Nacalai Tesque, No. 35556-44) and resuspended in 100 μL PBS. Cell cycle distribution was assessed by flow cytometry after staining cells with propidium iodide as described in the previous study^5, 7^. Briefly, floating and adherent cells were collected, washed with ice-cold PBS and fixed with 70% ethanol. The cells were then treated with stain solution (100 μg/ml RNase A (Wako), 1% Triton X-100 (Nacalai Tesque), 40 mM sodium citrate (Wako) and 50 μg/ml propidium iodide (Sigma) for 1 hour at room temperature in the dark. The stained cells were analyzed using a FACSCalibur flow cytometer (BD Biosciences). The cells treated with test compounds for 48 hours were collected and stained with propidium iodide using a BD CycletestTM Plus DNA Reagent Kit (Becton Dikinson and Company BD Biosciences) obtained from Phenix Flow Systems. Following FACS analysis, cell cycle distributions were further analyzed by Cell Quest software (BD Biosciences). The Phase fraction (%) is shown in the graph. For each sample, 1×10^4^ cells were recorded.

**Invasion assay.**

The in vitro invasion assay was performed using BioCoat Matrigel invasion chambers (24-well plate, 8 μm pore size; BD Biosciences No. 354480). PC-3 cells were seeded on the upper parts of the Transwell chamber at 1×10^5^ cells in 500 μL serum-free medium with 30 μmol/L test compounds (ATA1, ATA2, ATA3, Epi-2, Epi-3, Epi-4, Epi-5 or Epi-6). The chamber was placed into the 24-well plate, which contained 750 μL of RPMI1640 containing 10% charcoal-stripped FBS. These cultures were incubated for 36 h at 37 °C in a CO_2_ incubator. The cells that invaded to the lower surface of the filter were fixed and stained using Diff-Quick Stain Kit (Symex, Kobe, Japan). The invasive cells were counted in three random fields per chamber.

**Supplementary Figure 1 | Effects of test compounds on cell cycle distribution.** Effects of test compounds on cell cycle distribution were evaluated by FACS analysis. The experimental procedure for preparation of test compounds was described in Biochemical methods of Supplementary Information. The cells treated with epicatechin pentamer (Epi-5) for 48 h were collected and stained with propidium iodide using a BD Cycletest Plus DNA Reagent Kit (BD Biosciences) obtained from Phoenix Flow Systems. Following FACS analysis, cell cycle distributions were further analyzed by Cell Quest software. The data are the means ± S.D. of three independent experiments. Two-way ANOVA followed by Dunnett's multiple comparison test was used to compare means (a). **P* < 0.05 Images of cell cycle distribution after cells were treated with Epi-5 (b). Effect of Epi-5 on cell cycle distribution was evaluated using FACSCalibur flow cytometer (BD Biosciences) and further analyzed by Cell Quest software (BD Biosciences). The cells treated with ethanol (6 % v/v) alone (control) or 200 μmol/L epicatechin pentamer (Epi-5) for 48 h were collected and stained with propidium iodide stain solution.


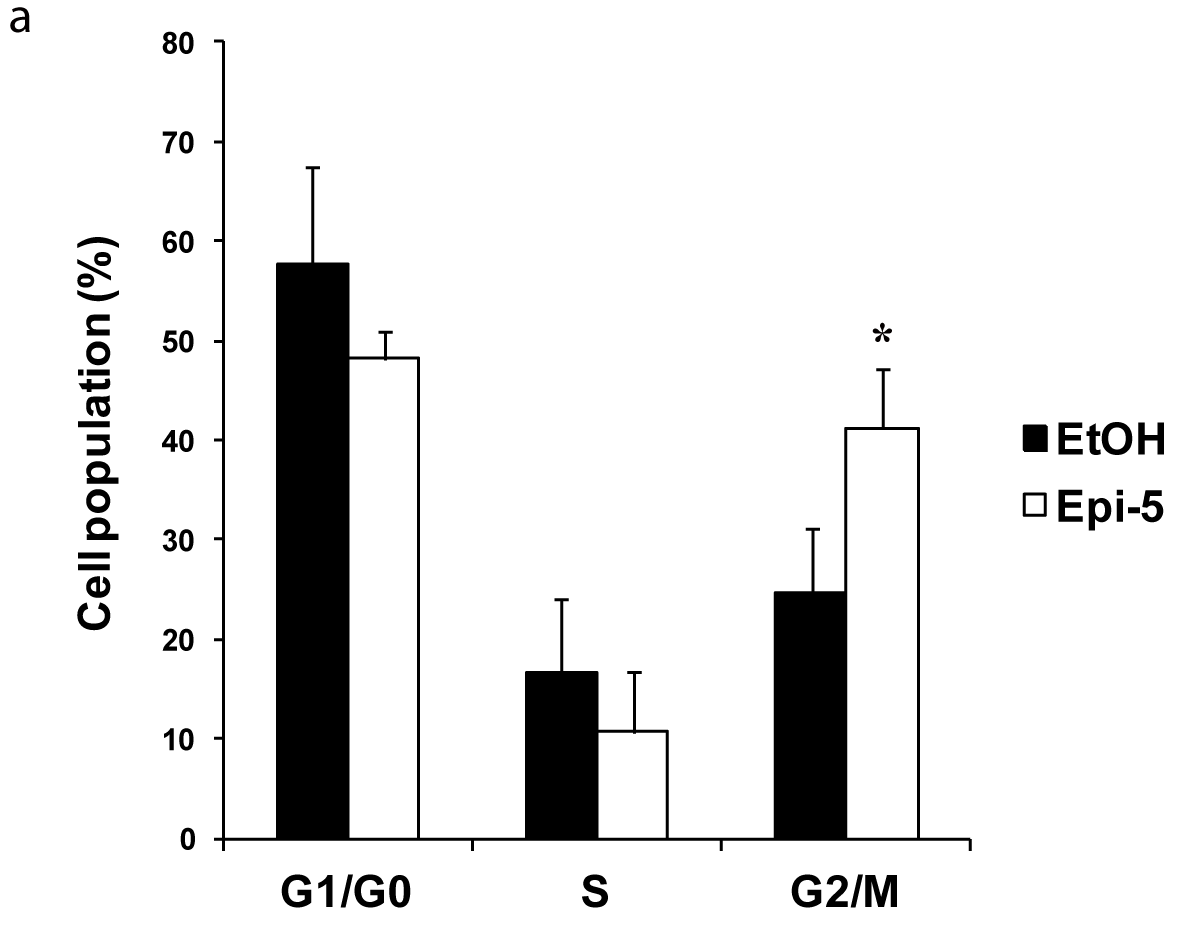

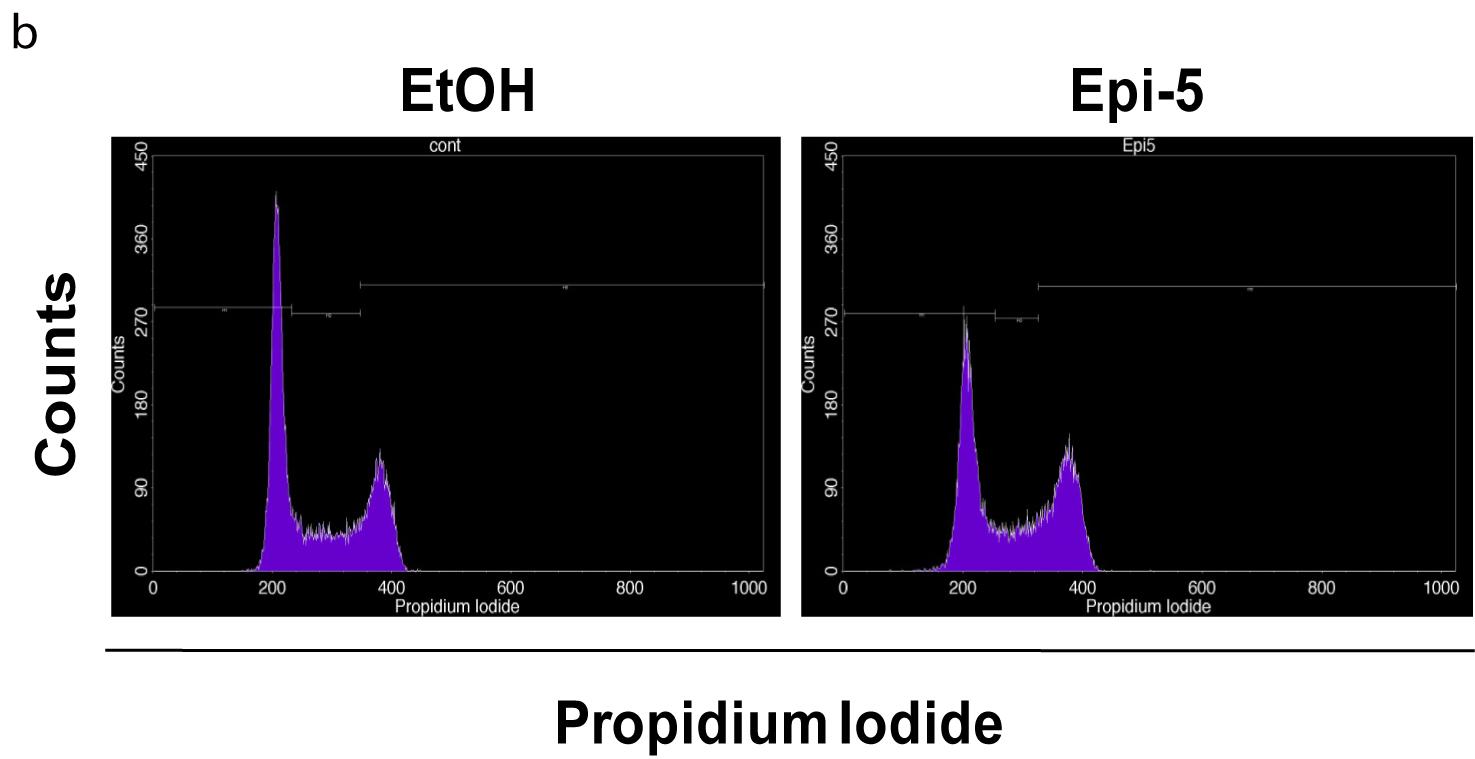


**Supplementary Figure 2 | Full images of Western blot analyses of the expression of the cancer-promoting gene *FABP5* by treatment of test compounds.**

The experimental procedures are described in Biochemical methods and Fig.7.


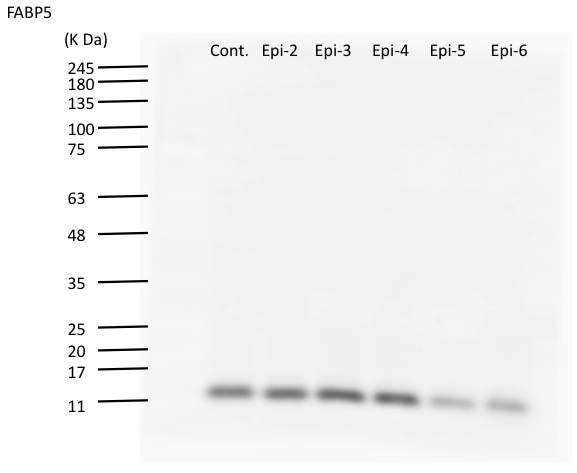

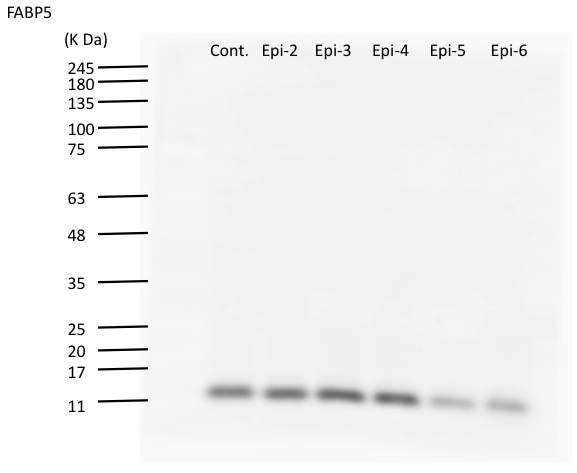

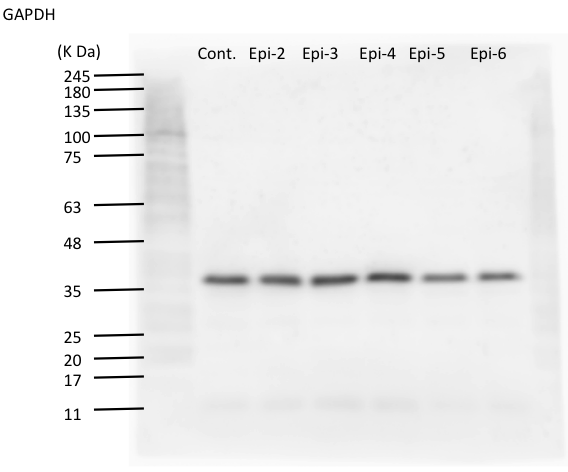


**Supplementary references**

1. Saito, A., Mizushina, Y., Tanaka, A. & Nakajima, N. [Versatile synthesis of epicatechin series procyanidin oligomers, and their antioxidant and DNA polymerase inhibitory activity](http://www.sciencedirect.com/science/article/pii/S0040402009010497). *Tetrahedron* **65**, 7422-7428 (2009).
2. Kozikowski, A. P., Tückmantel, W., Böttcher, G. & Romanczyk, Jr. L. J. Studies in polyphenol chemistry and bioactivity. 4. Synthesis of trimeric, tetrameric, pentameric, and higher oligomeric epicatechin-derived procyanidins having all-4β,8-interflavan connectivity and their inhibition of cancer cell growth through cell cycle arrest. *J. Org. Chem.* **68**, 1641-1658 (2003).
3. Ohmori, K., Shono, T., Hatakoshi, Y., Yano, T. & Suzuki, K. Integrated synthetic strategy for higher catechin oligomers. *Angew. Chem. Int. Ed.* **50**, 4862-4867 (2011).
4. DeBiaso R., Bgight G. R., Ernst L. A., Waggoner A. S. & Taylor D. L. Five-parameter fluorescence imaging: Wound-healing of living Swiss 3T3 cells. *J. Cell Biol.* **105**, 1613-1622 (1987).
5. Fujii, W. *et al.* Syntheses of prodelphinidin B3 and C2, and their antitumor activities through cell cycle arrest and caspase-3 activation. *Tetrahedron* **69**, 3543-3550 (2013).
6. Kawaguchi, K. *et al.* The cancer-​promoting gene fatty acid-​binding protein 5 (FABP5) is epigenetically regulated during human prostate carcinogenesis. *Biochem. J.* **48**, 449-462, (2016).
7. Kawaguchi, K. *et al.* High expression of fatty acid-​binding protein 5 promotes cell growth and metastatic potential of colorectal cancer cells. *FEBS Open Bio* **6**, 190-199 (2016).

**^1^H-NMR of 10.**

**
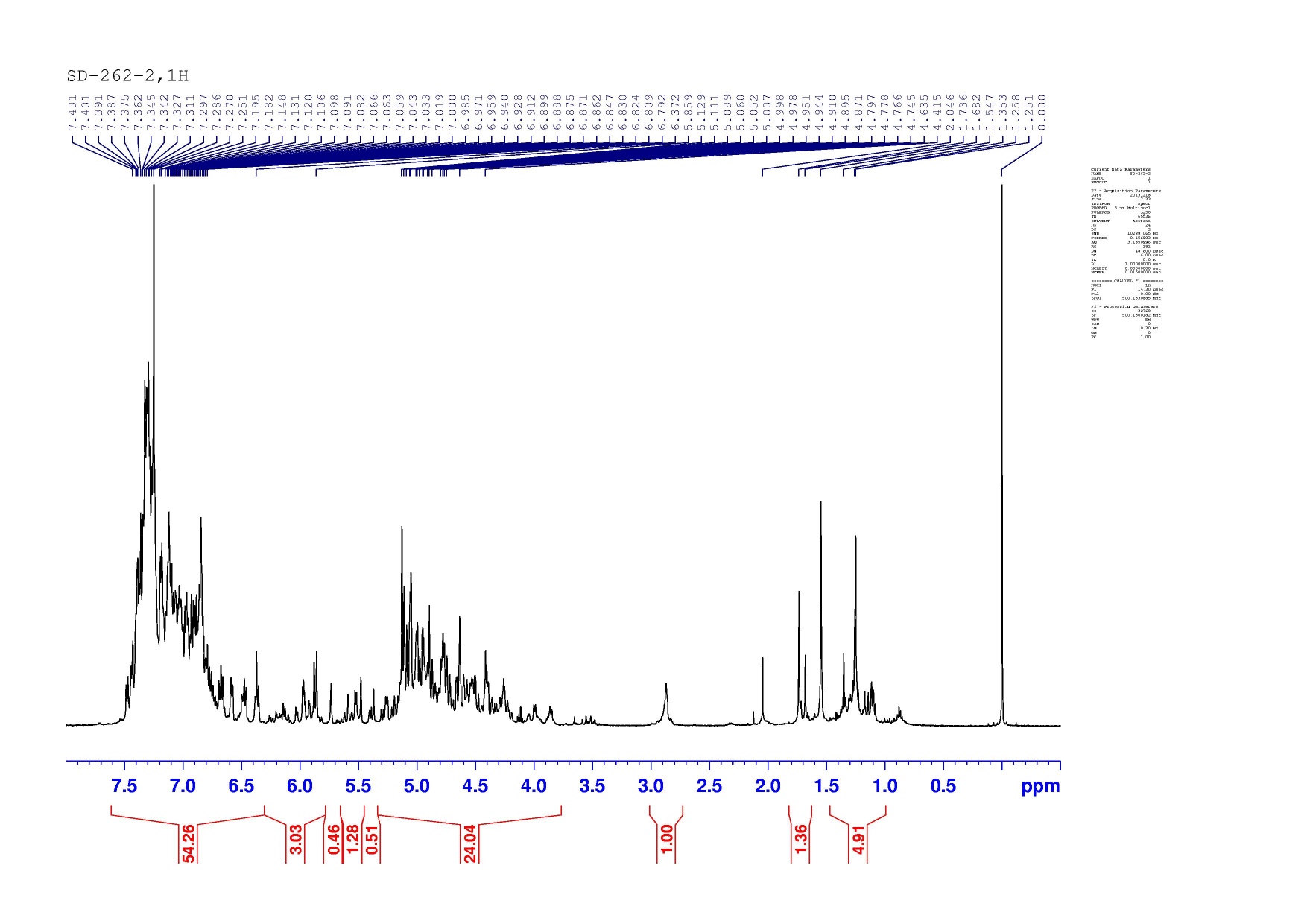
**

**^13^C-NMR of 10.**

**^1^H-NMR of 12.**

**
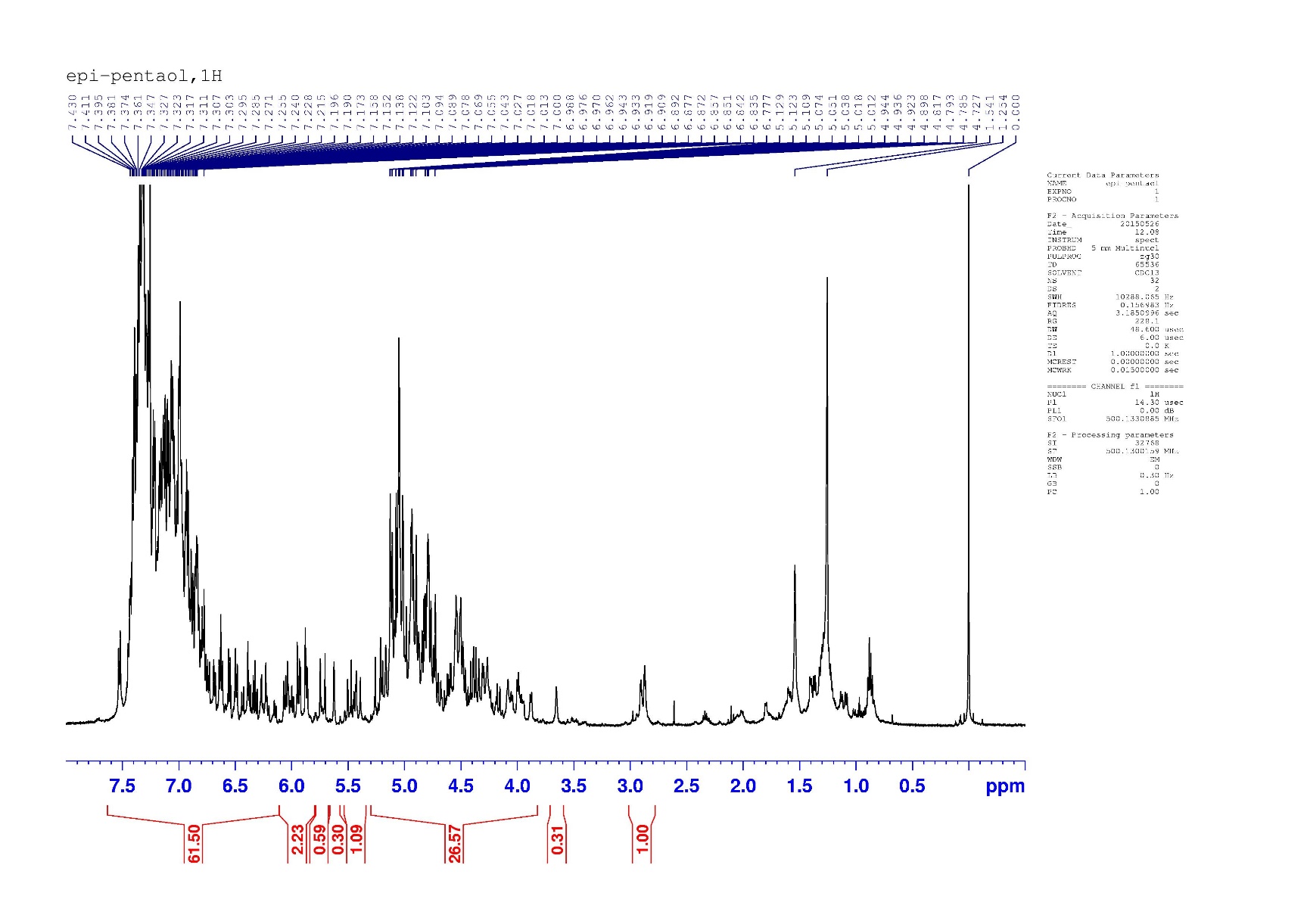
**

**^13^C-NMR of 12.**

**
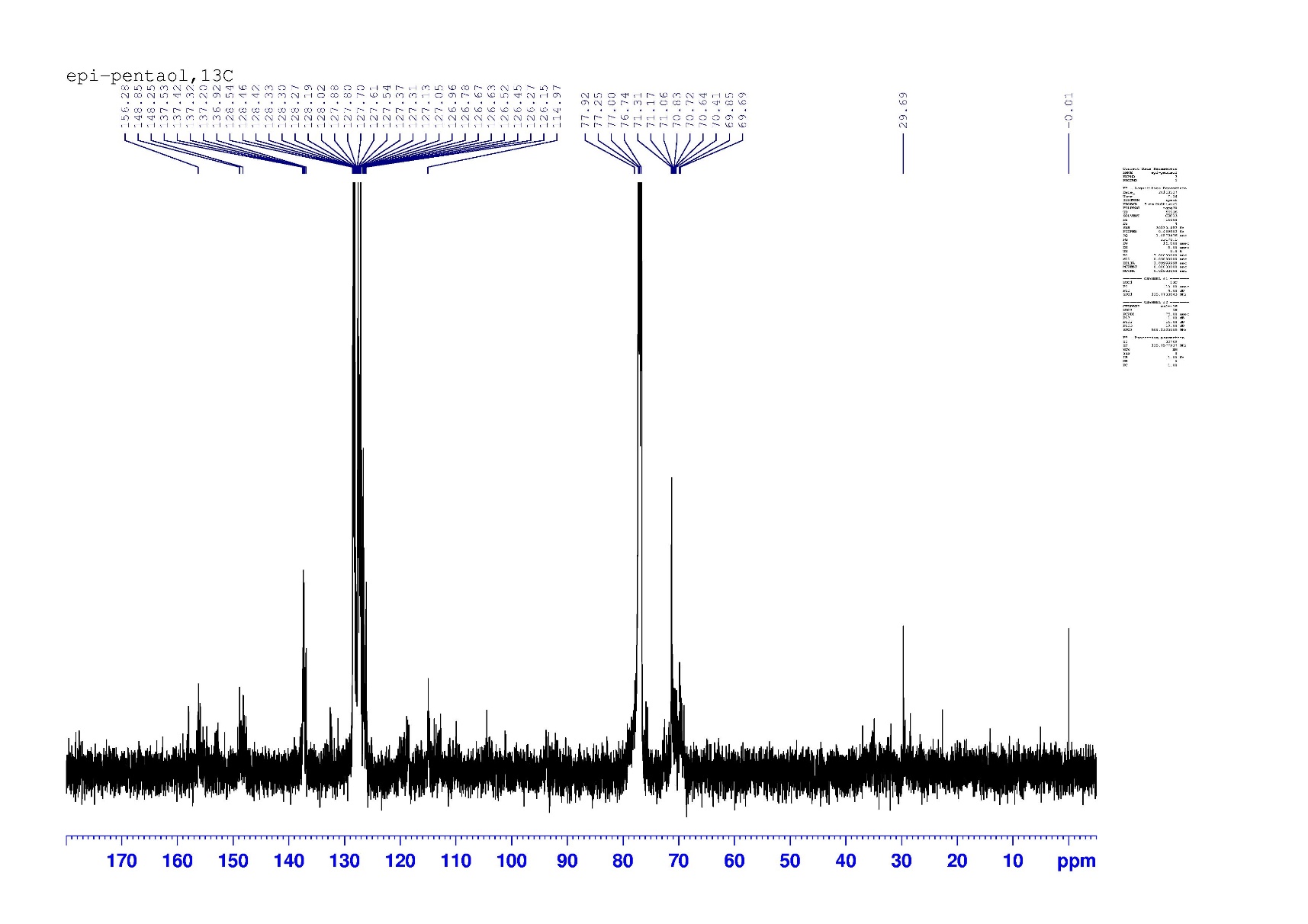
**

**^1^H-NMR of 1.**

**^13^C-NMR of 1.**

**ESITOFMS of 1 (MS-A).**

**HPLC data of 1 (HPLC-A).**

**^1^H-NMR of 11.**

**^13^C-NMR of 11.**

**^1^H-NMR of 13.**

**^13^C-NMR of 13.**

**^1^H-NMR of 2.**

**^13^C-NMR of 2.**

**ESI-TOFMS of 2 (MS-A).**

**HPLC data of 2 (HPLC-A).**

**^1^H-NMR of 19**

**
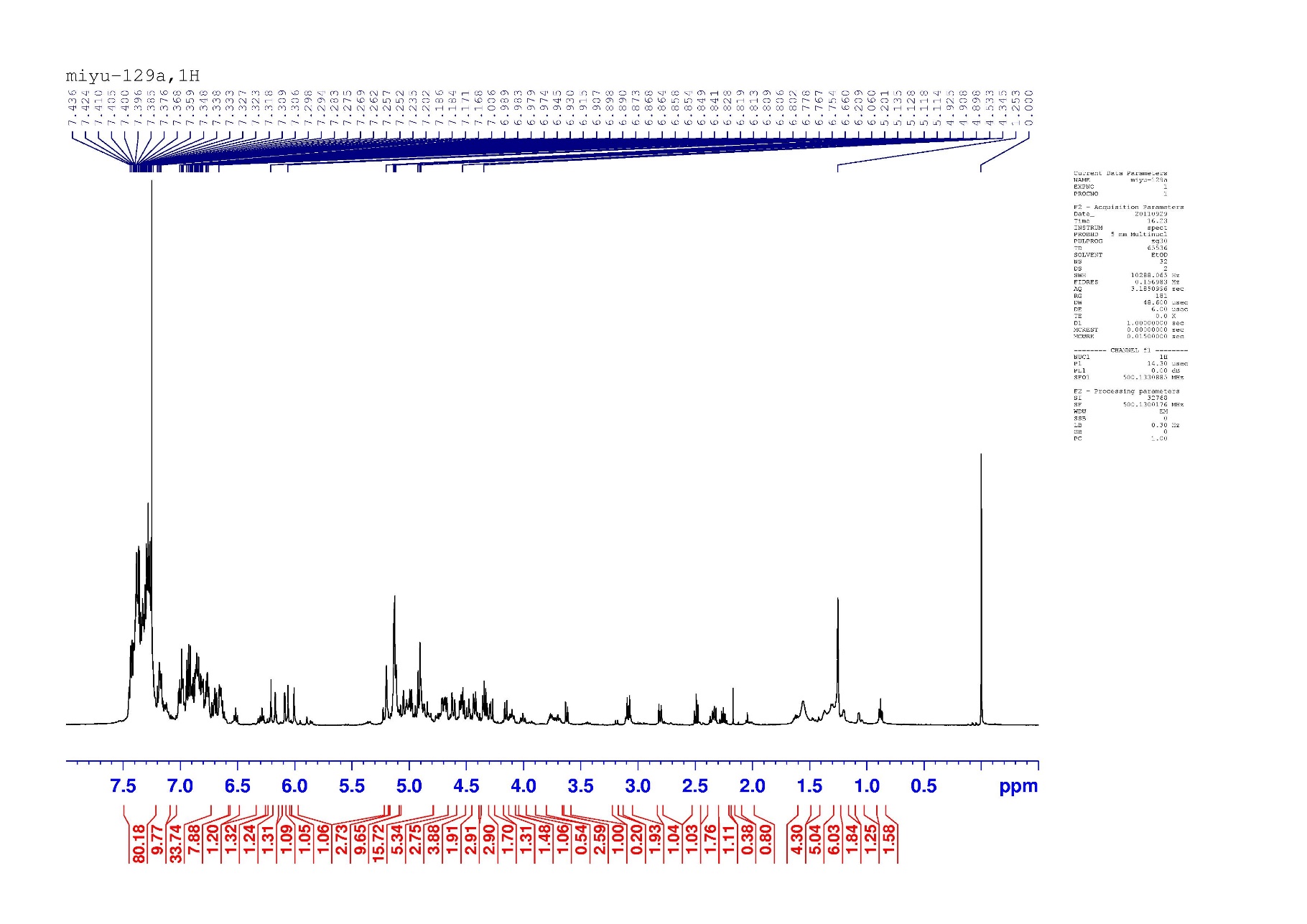
**

**^13^C-NMR of 19**

**
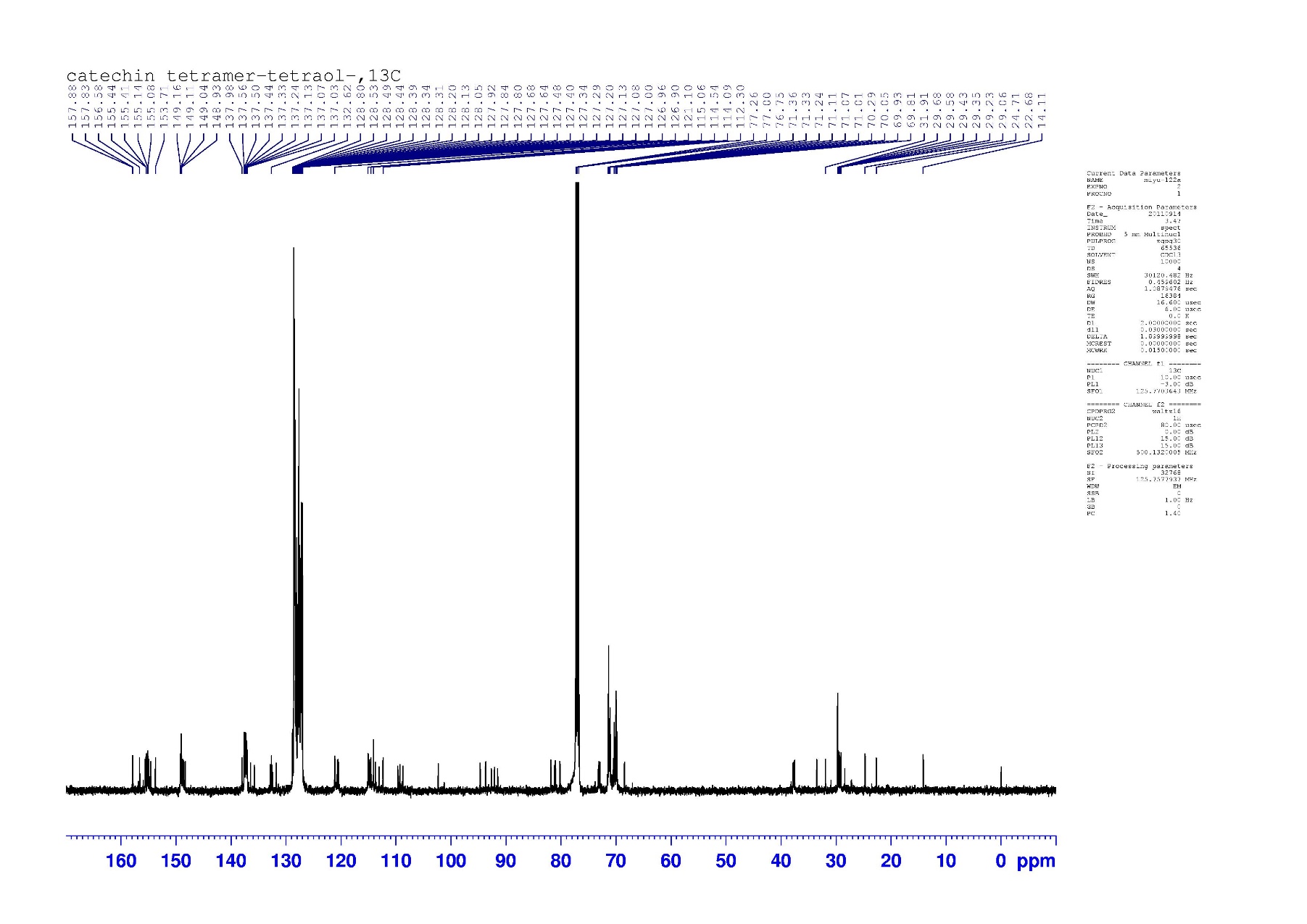
**

**^1^H-NMR of 3**

**
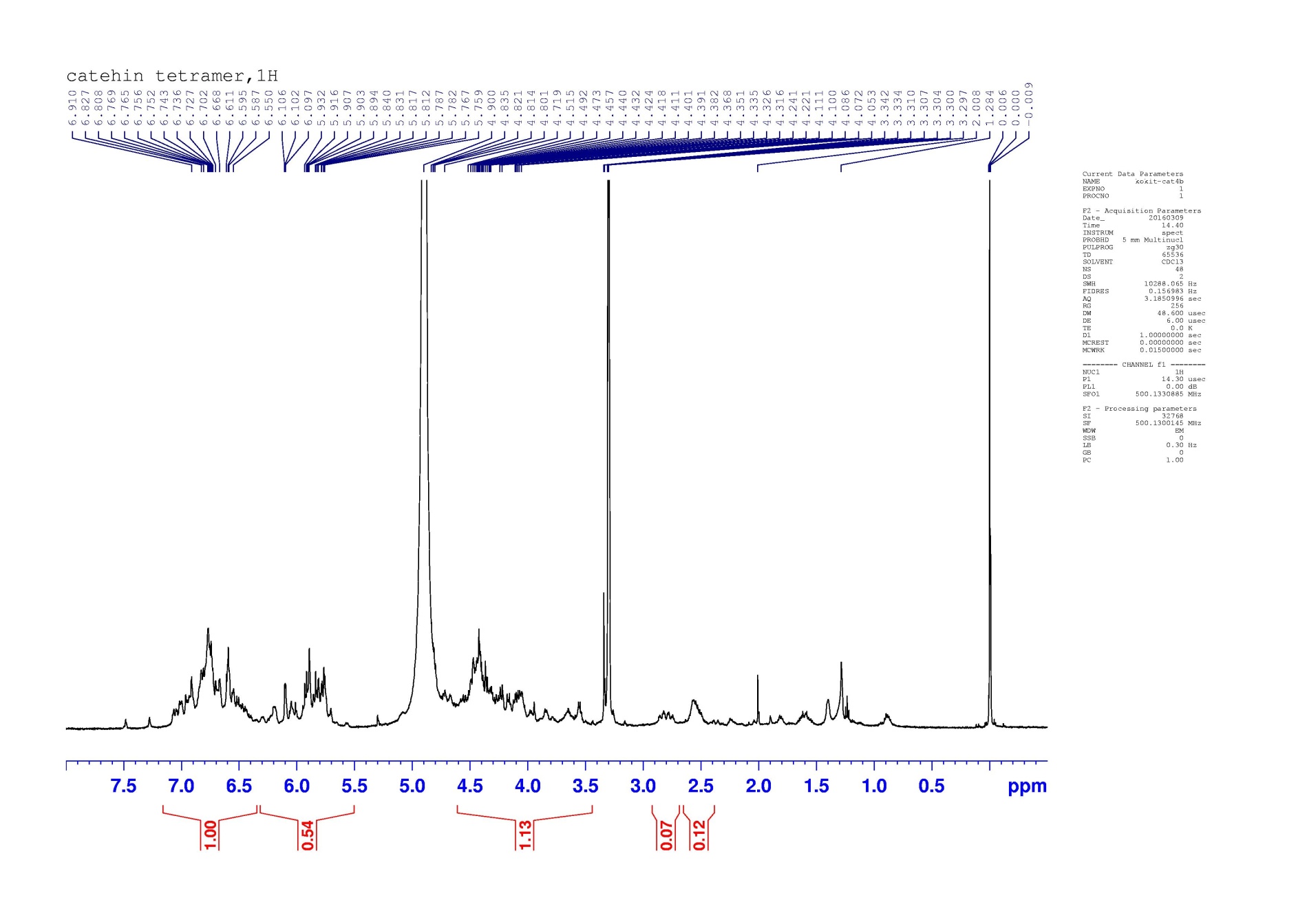
**

**^13^C-NMR of 3.**

**ESIMS of 3 (MS-A).**

**HPLC data of 3 (HPLC-A).**

**^1^H-NMR of 20.**

**
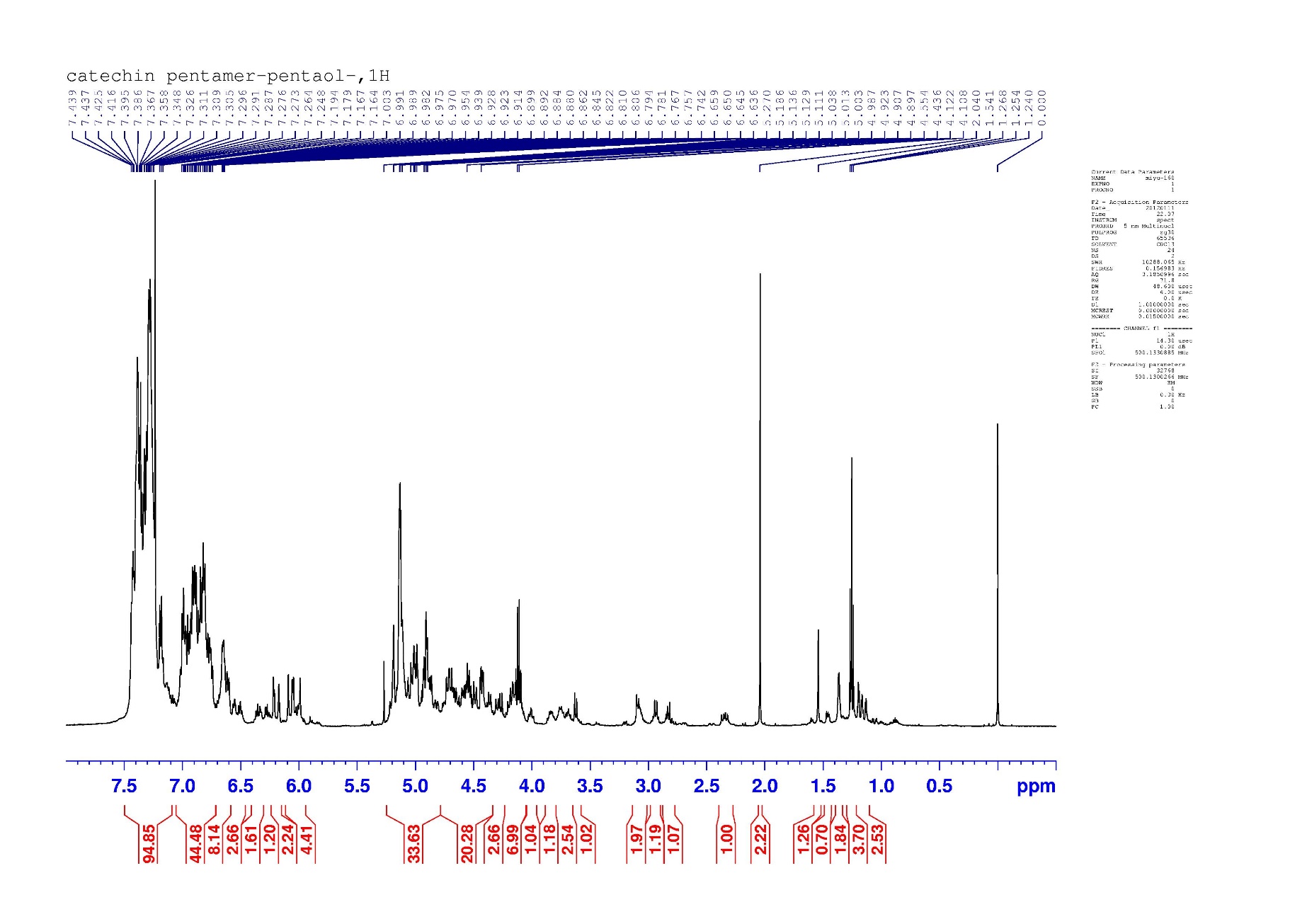
**

**^13^C-NMR of 20.**

**
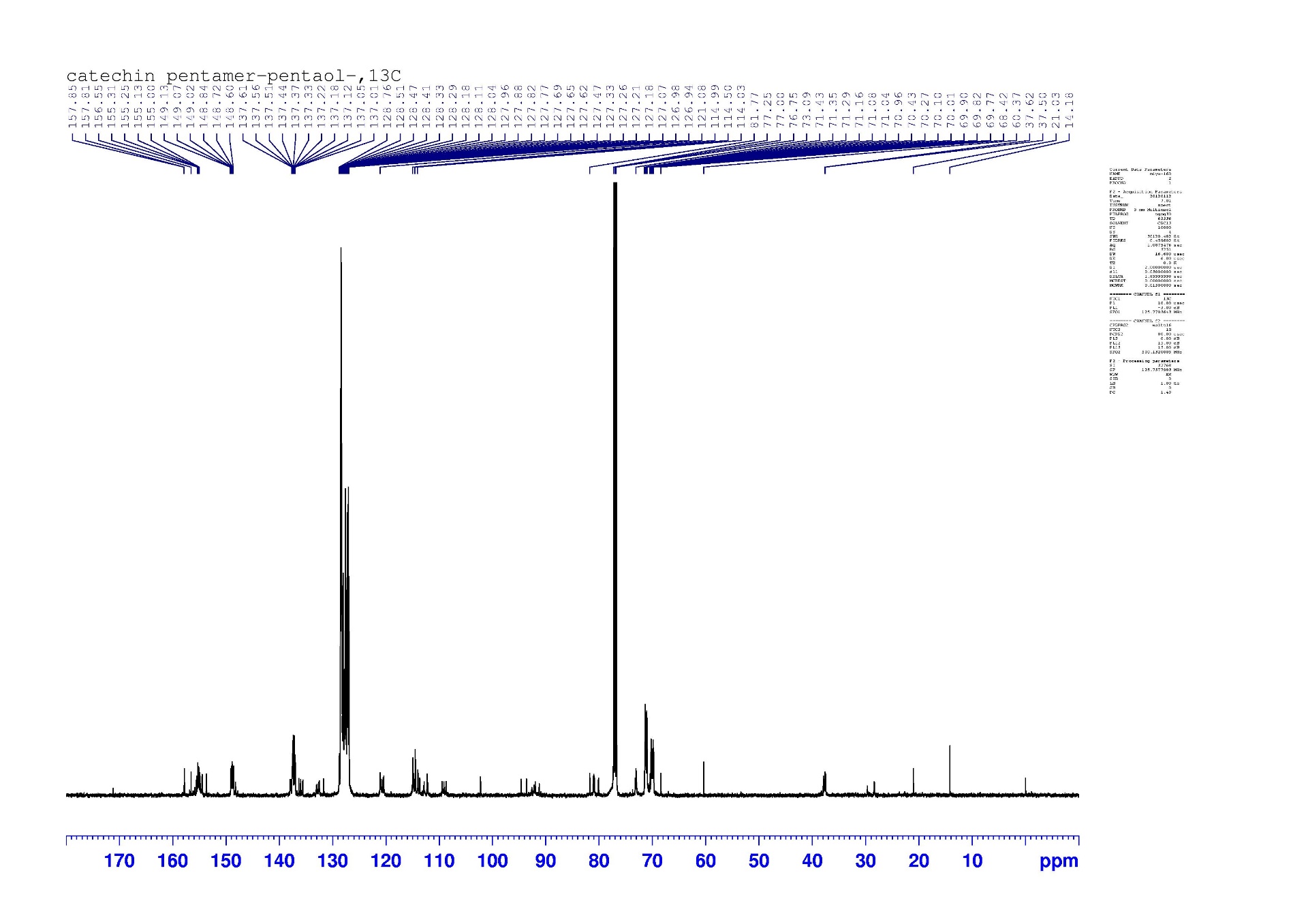
**

**^1^H-NMR of 4.**


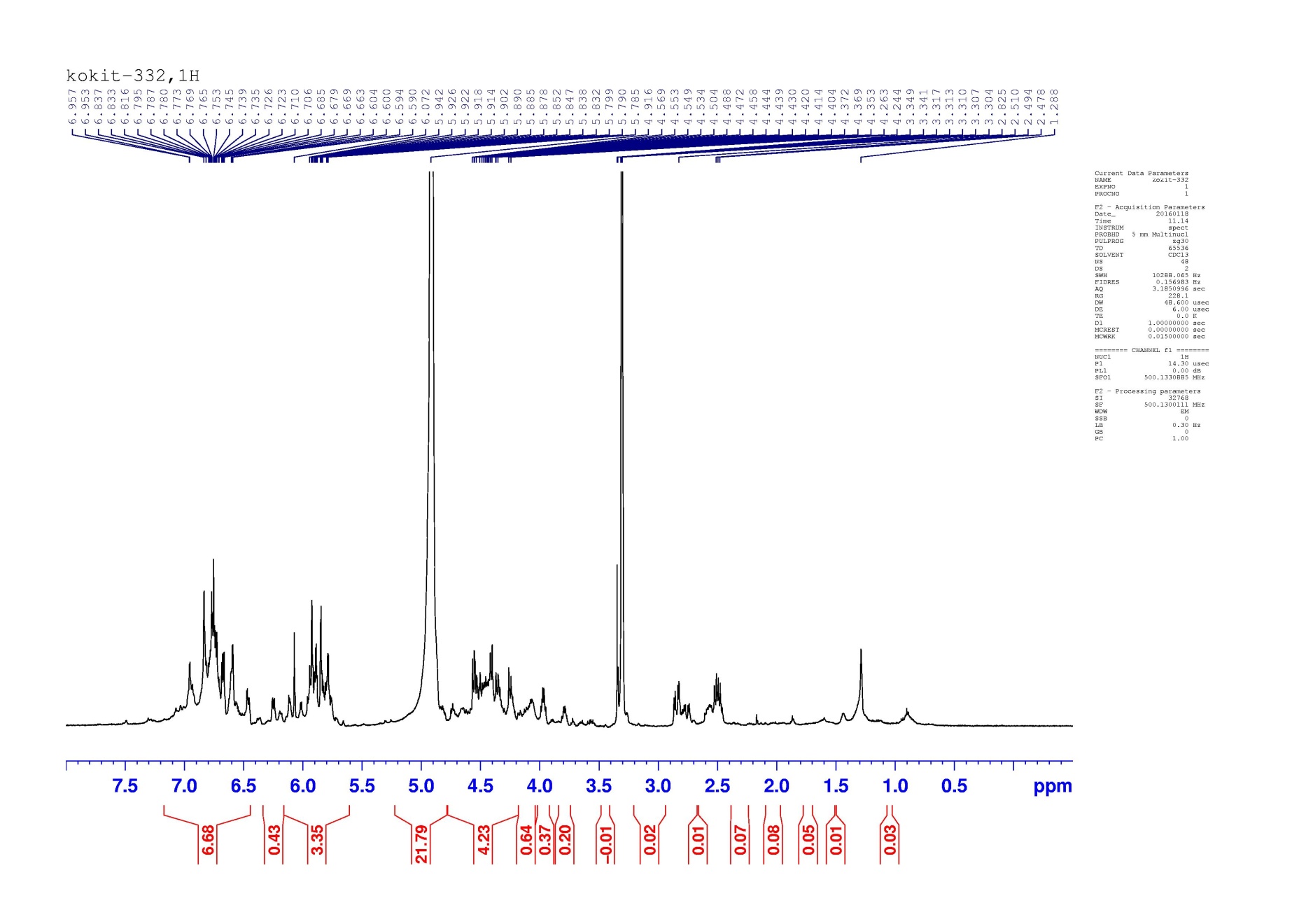


**^13^C-NMR of 4.**


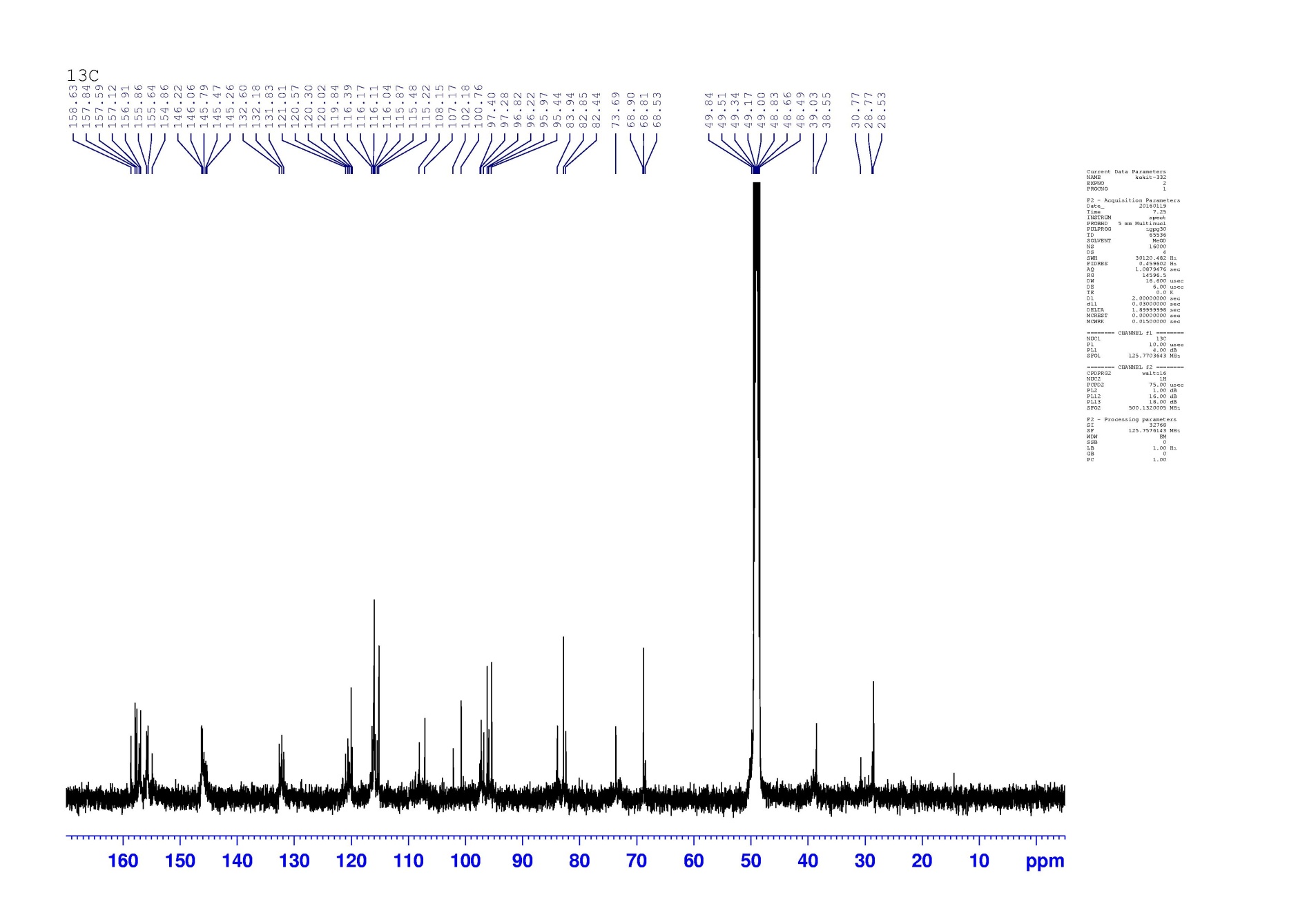


**ESI-TOFMS of 4 (MS-A)**

**HPLC data of 4 (HPLC-A).**

**^1^H-NMR of 23.**

**
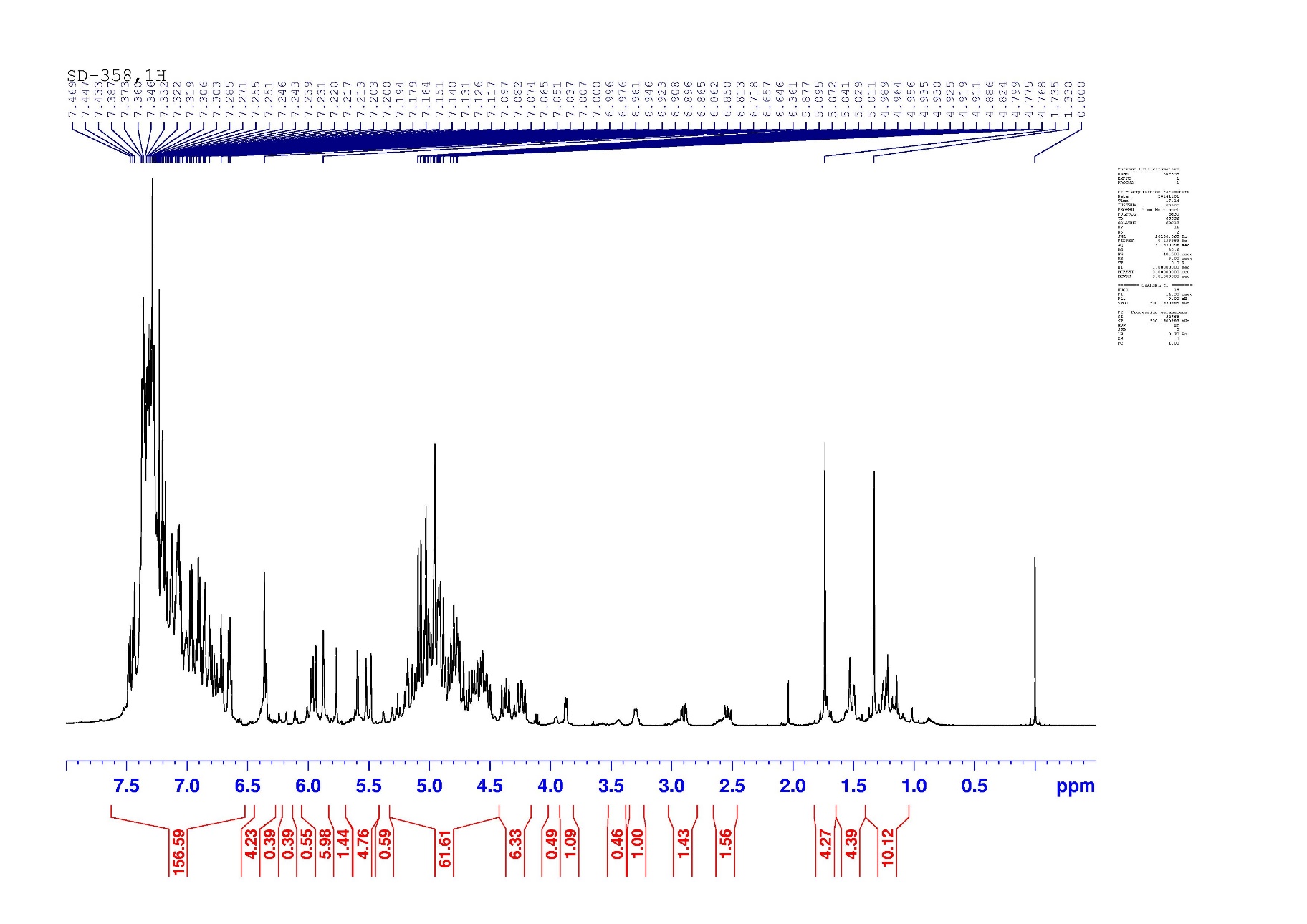
**

**^13^C-NMR of 23.**

**
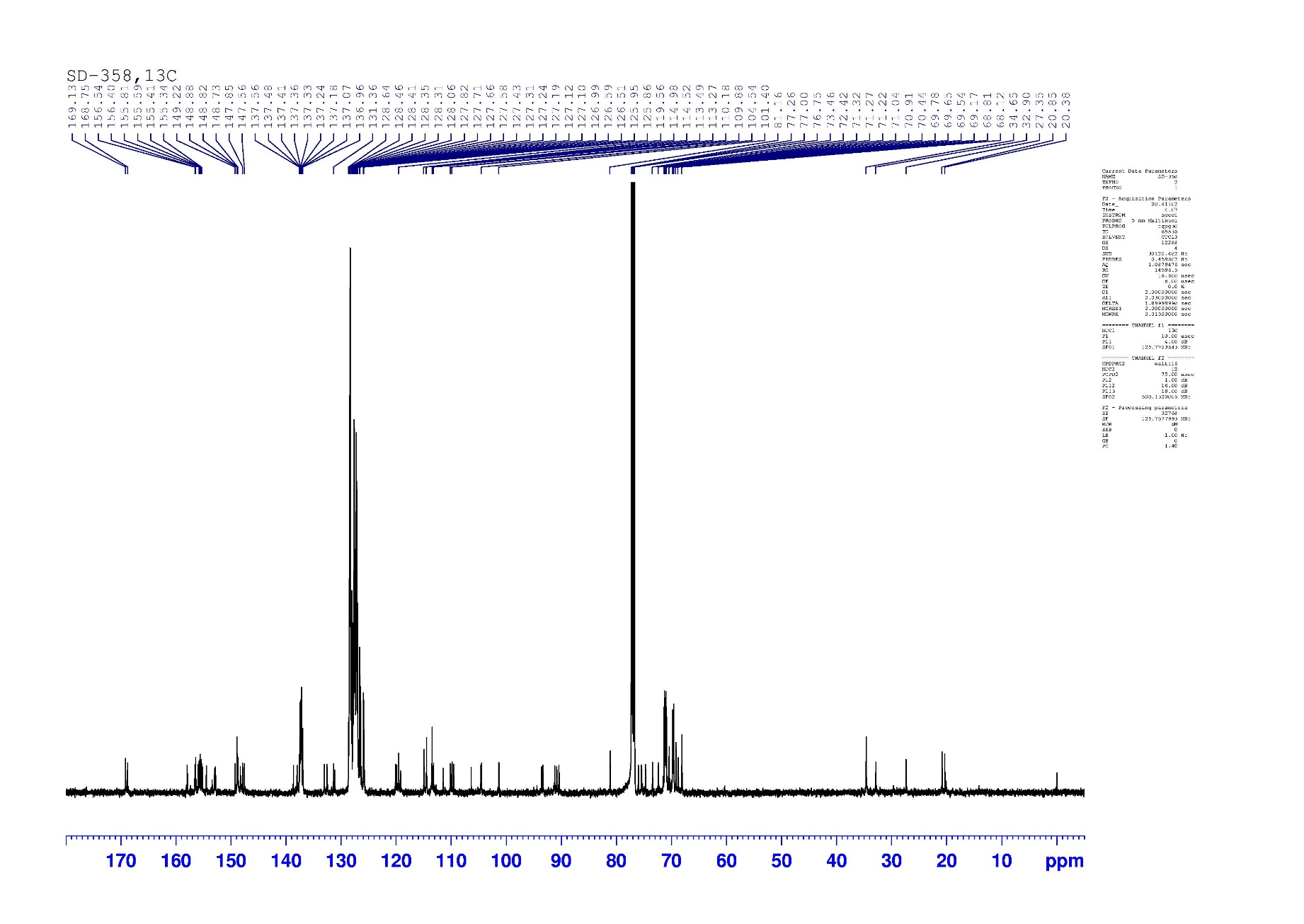
**

**^1^H-NMR of 25.**

**
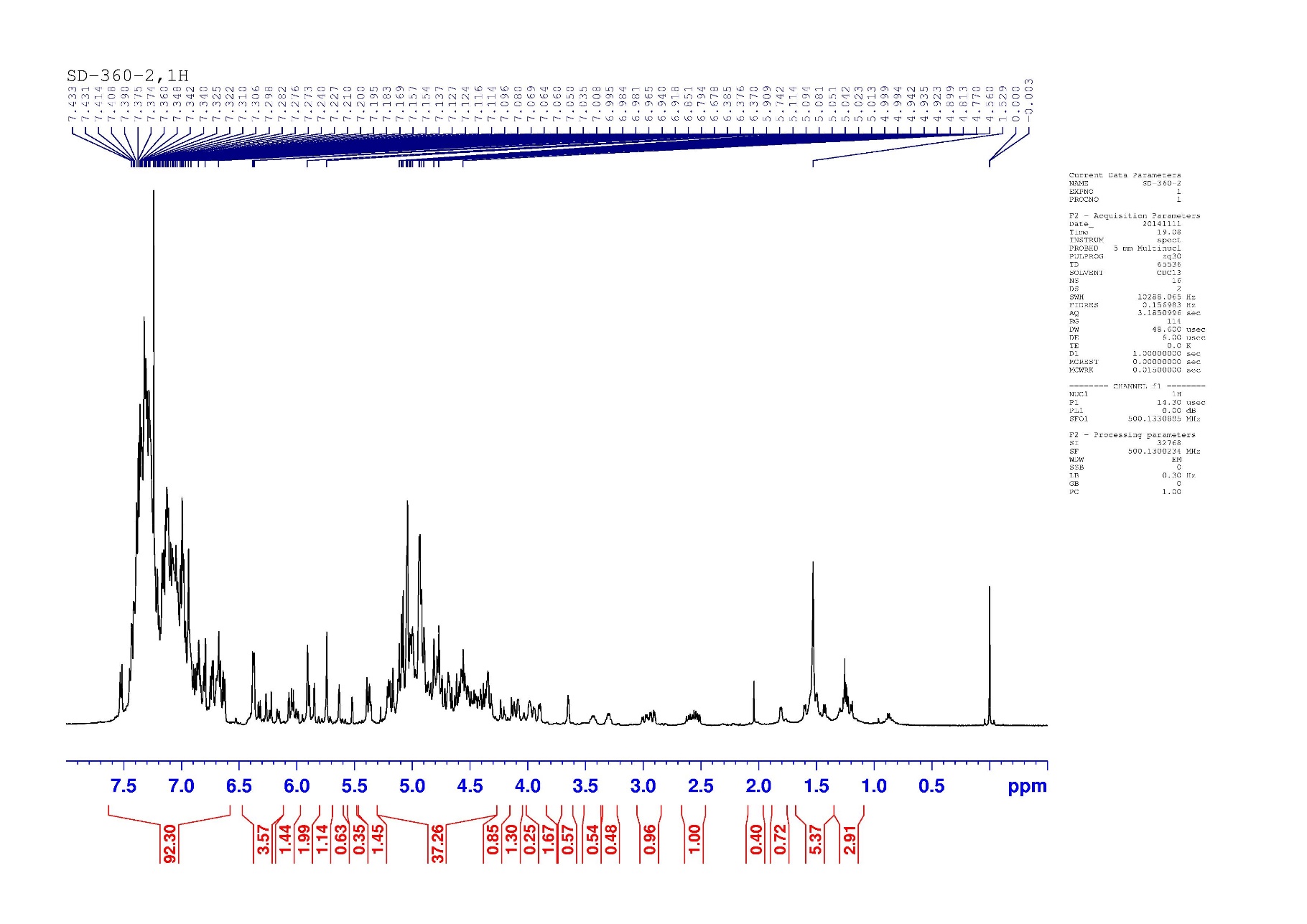
**

**^13^C-NMR of 25.**

**
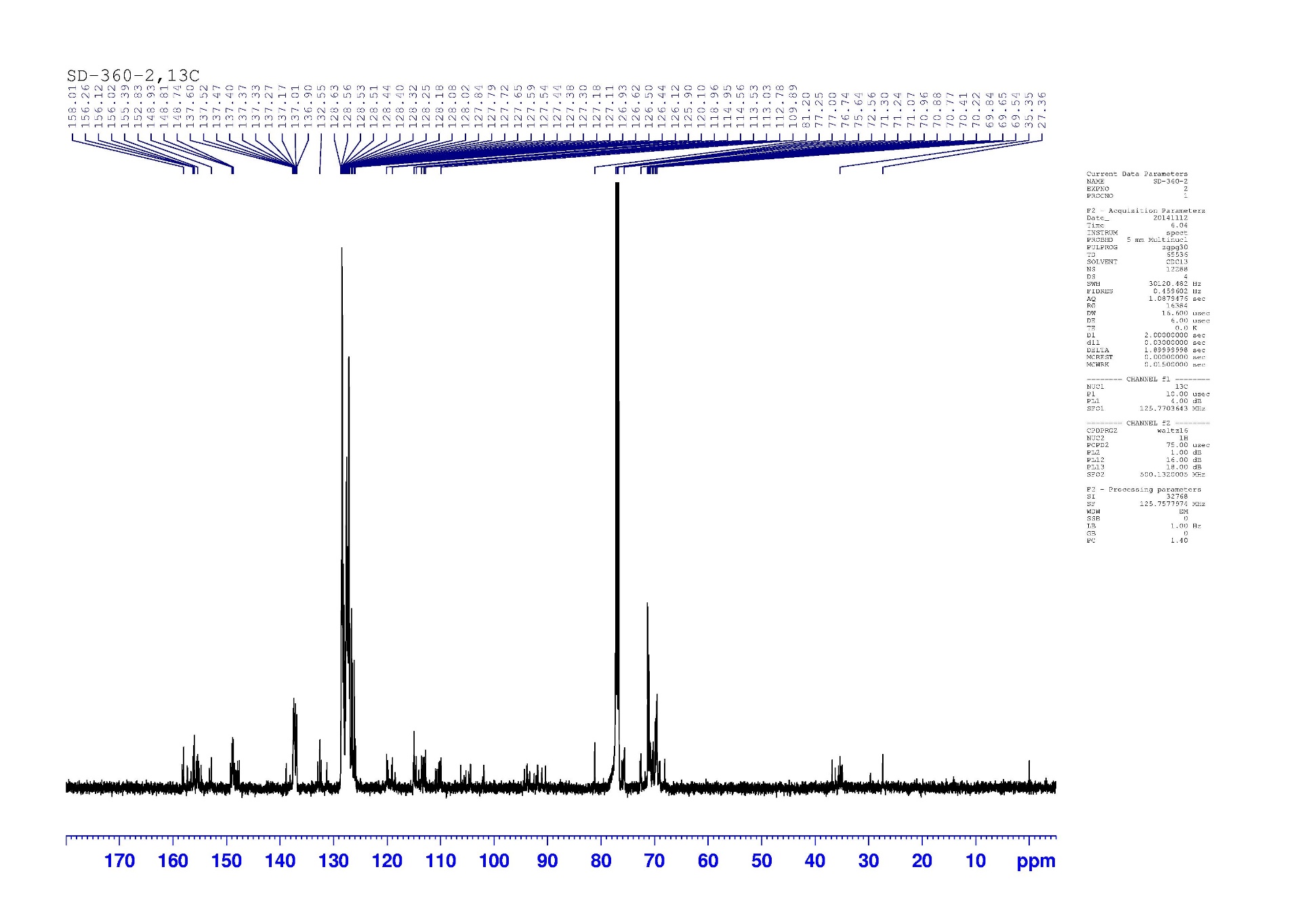
**

**^1^H-NMR of 5.**


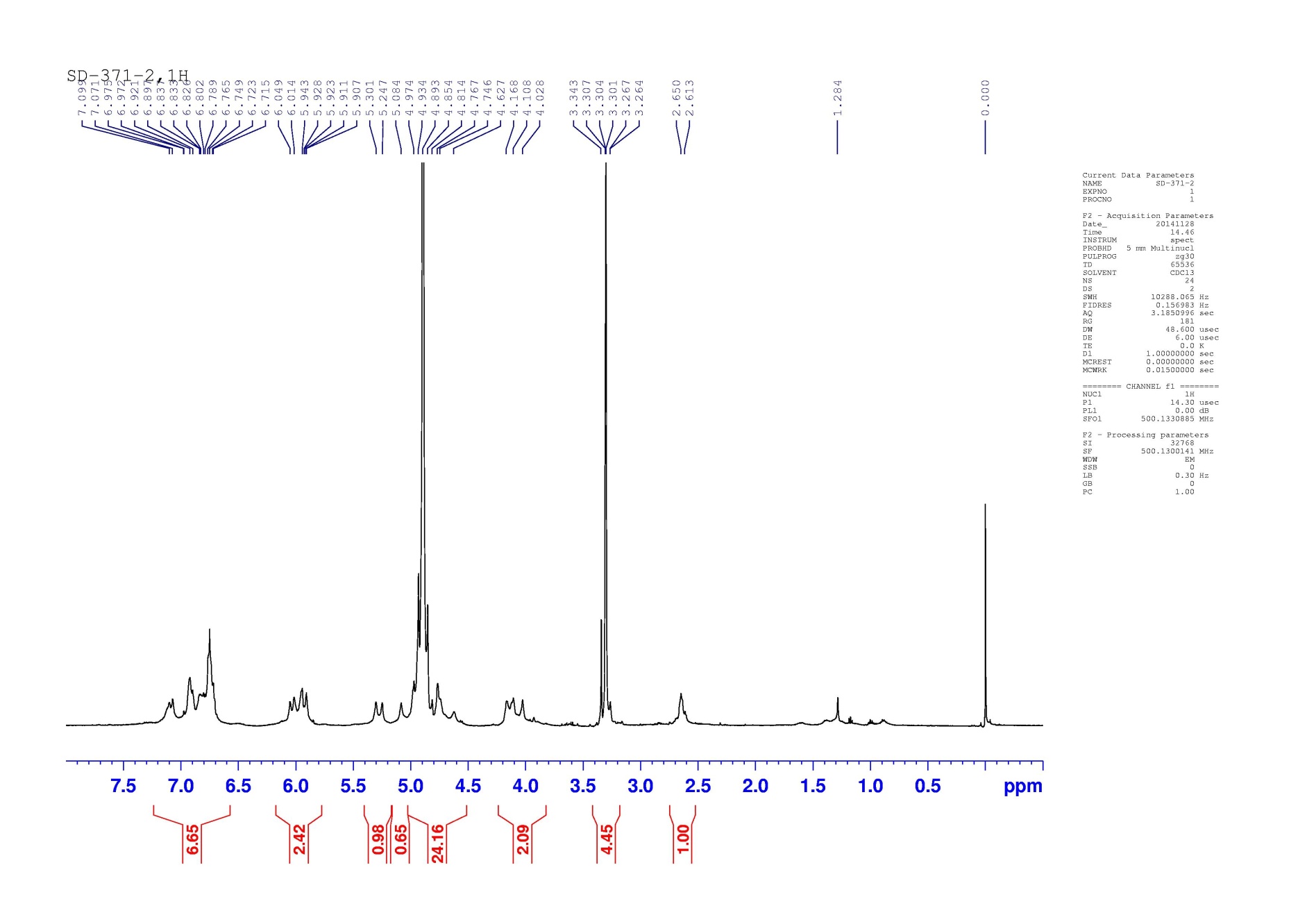


**^13^C-NMR of 5.**


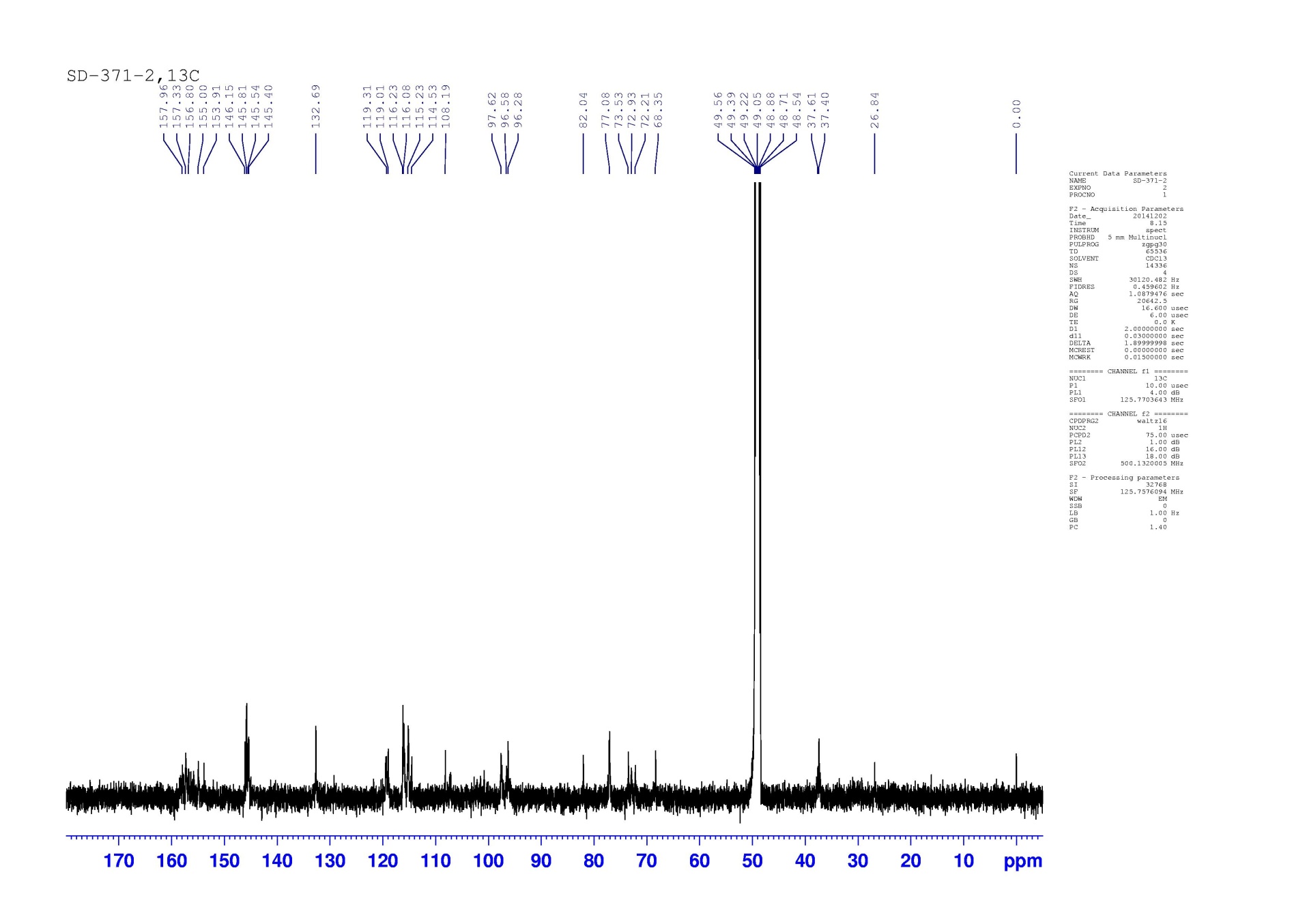


**ESI-TOFMS of 5 (MS-B).**

**HPLC data of 5 (HPLC-B).**


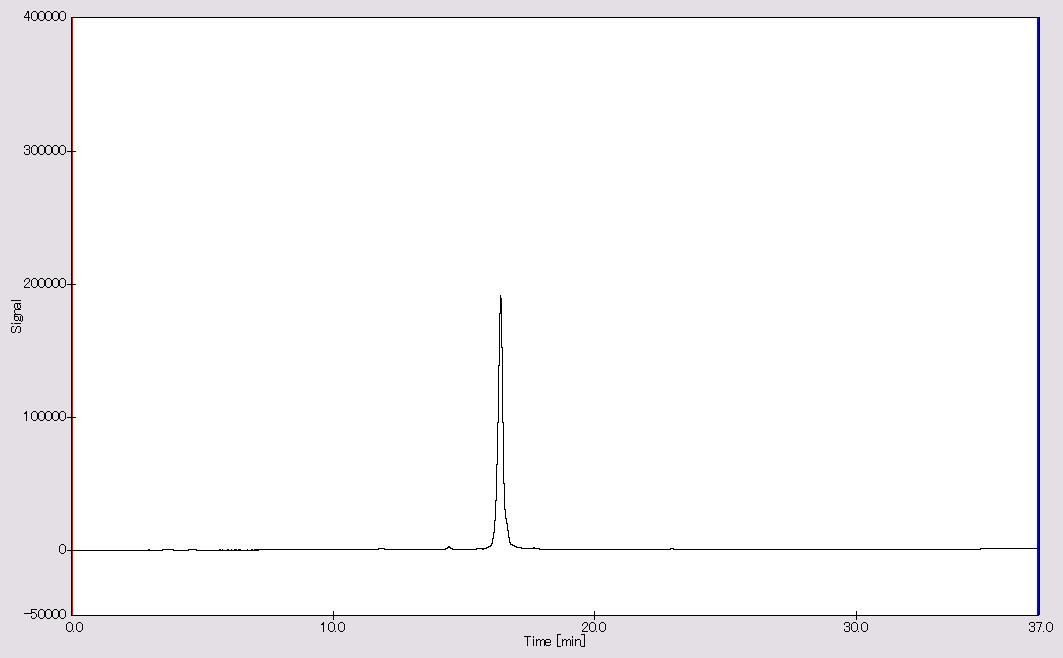


**^1^H-NMR of 24.**

**
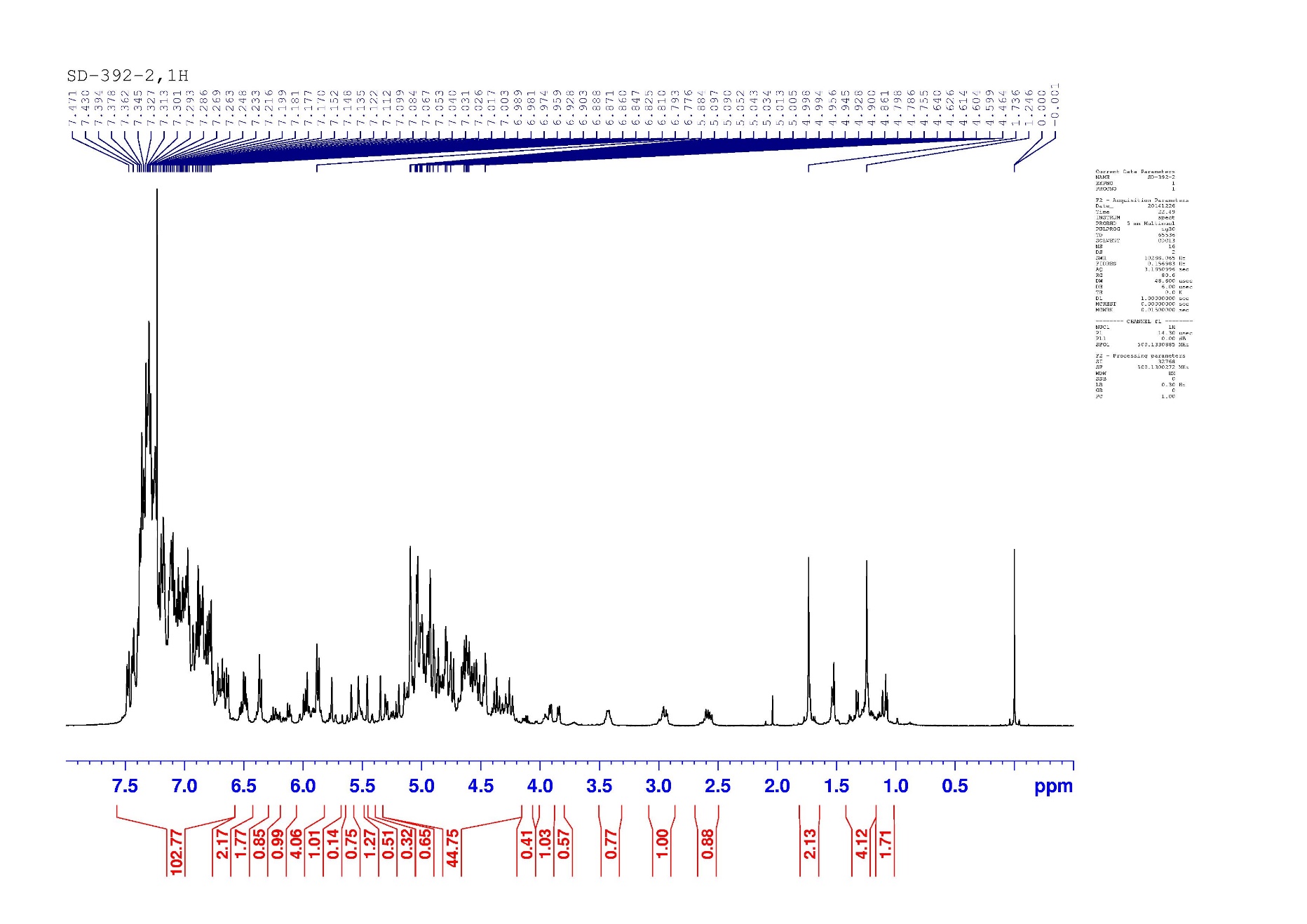
**

**^13^C-NMR of 24.**

**
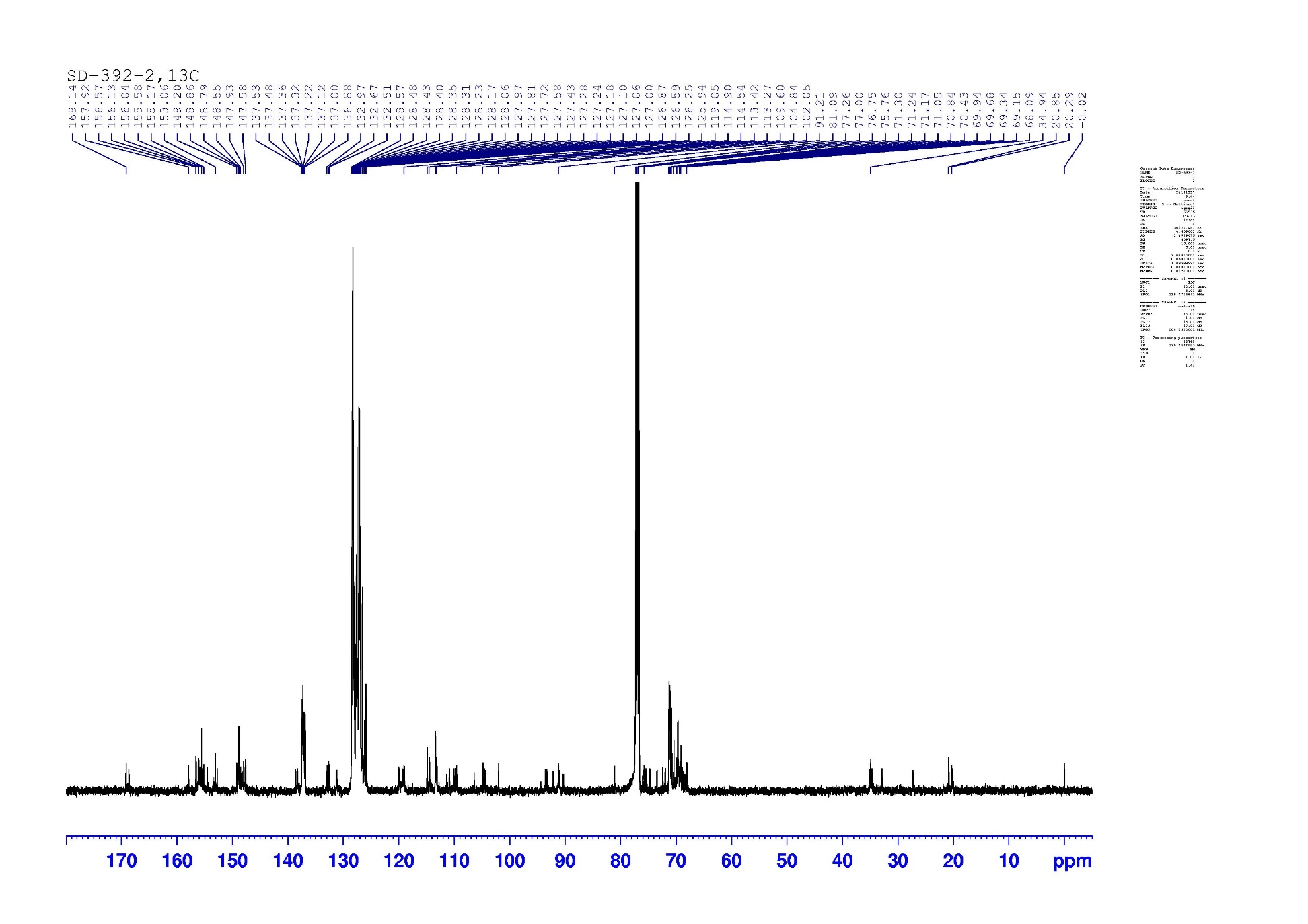
**

**^1^H-NMR of 26.**

**
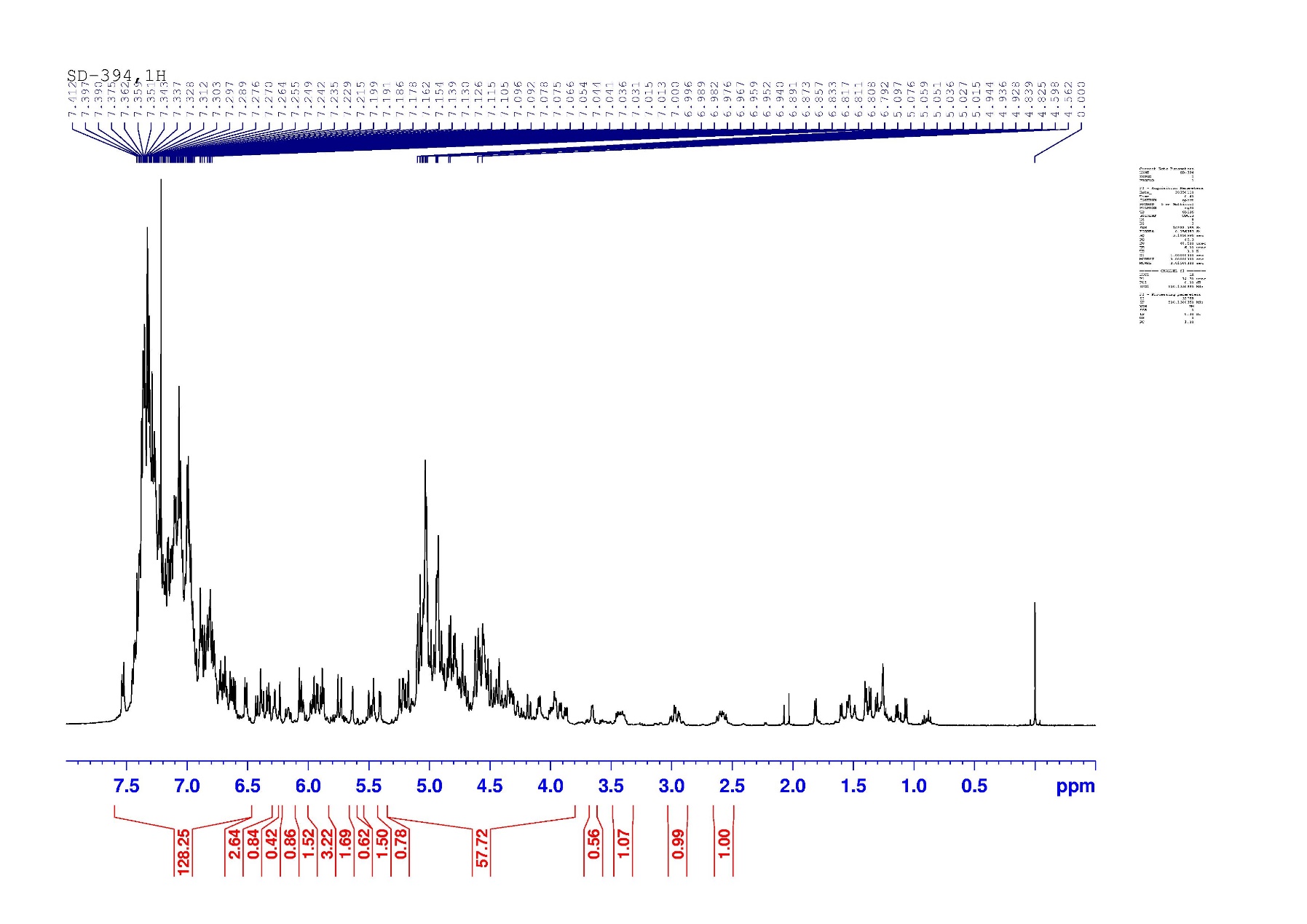
**

**^13^C-NMR of 26.**

**
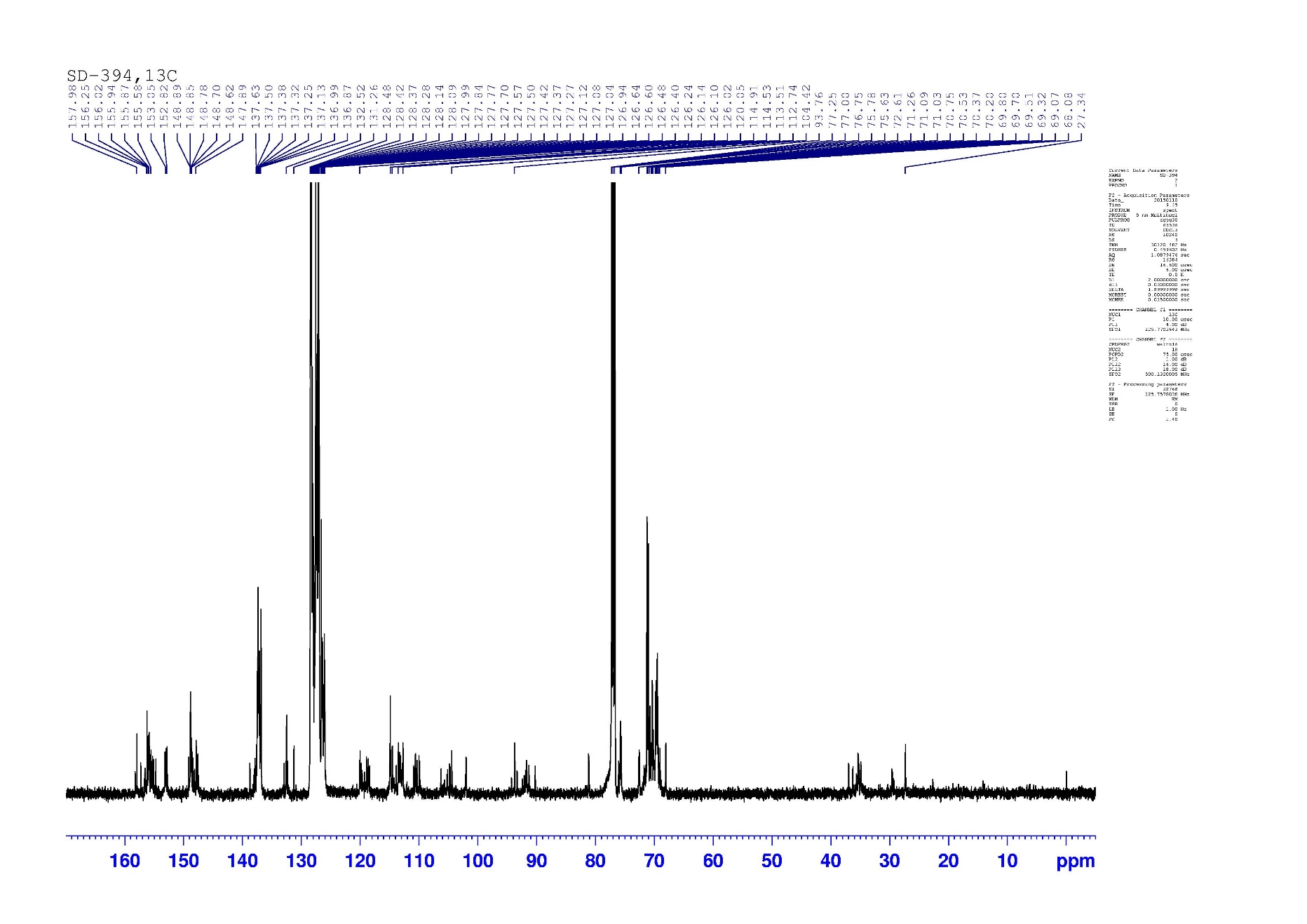
**

**^1^H-NMR of 6.**


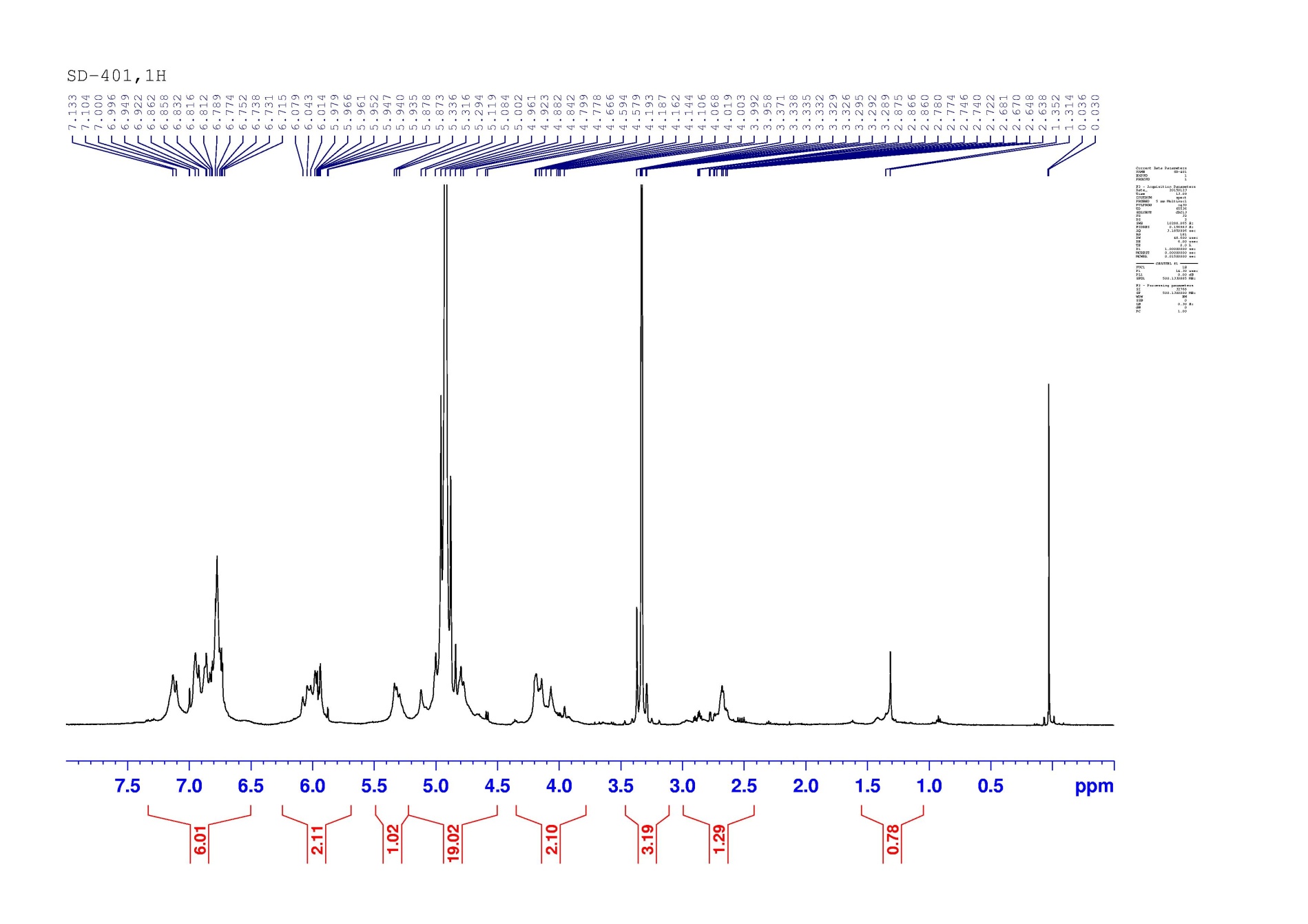


**^13^C-NMR of 6.**


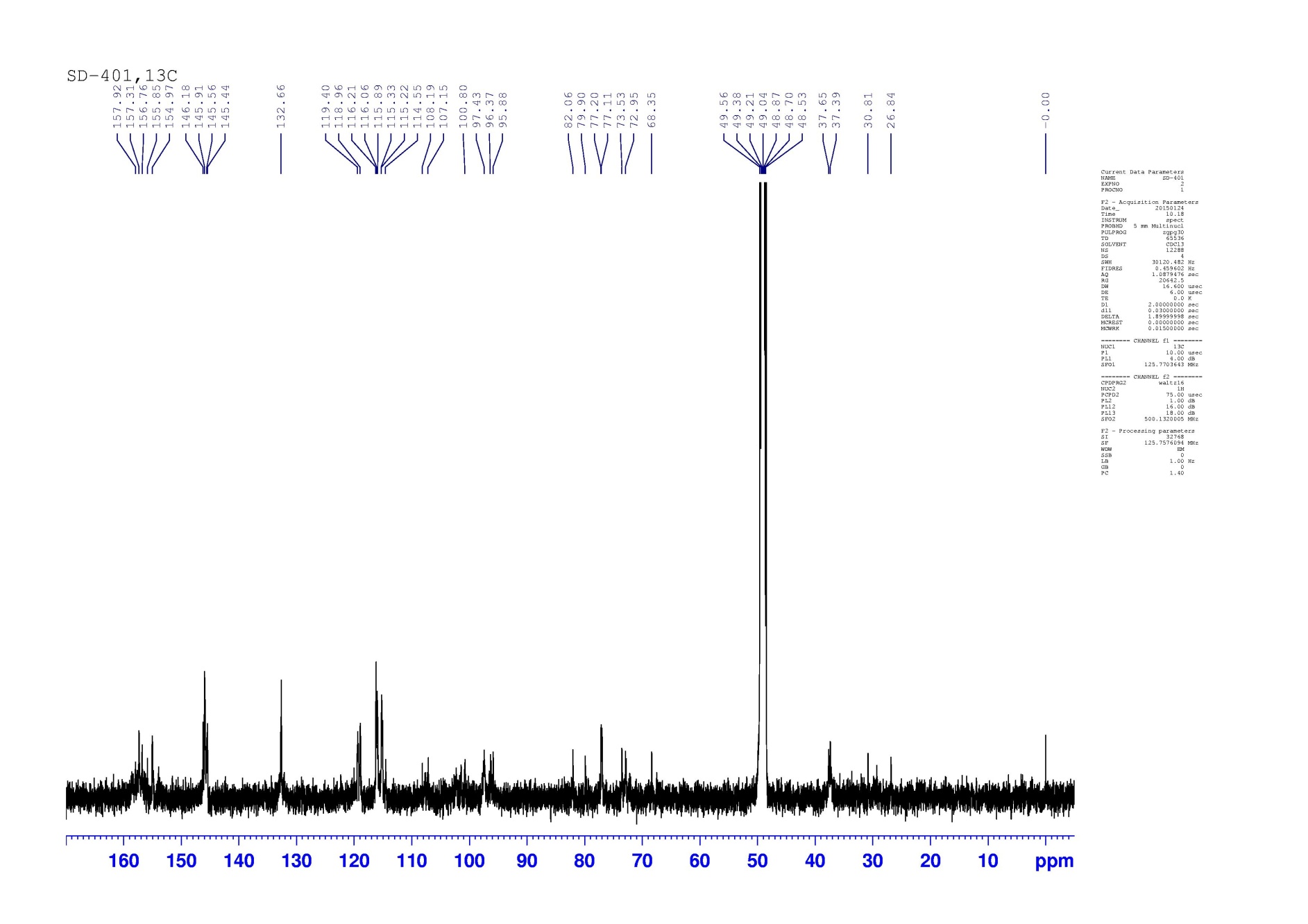


**ESI-TOMS of 6 (MS-B).**


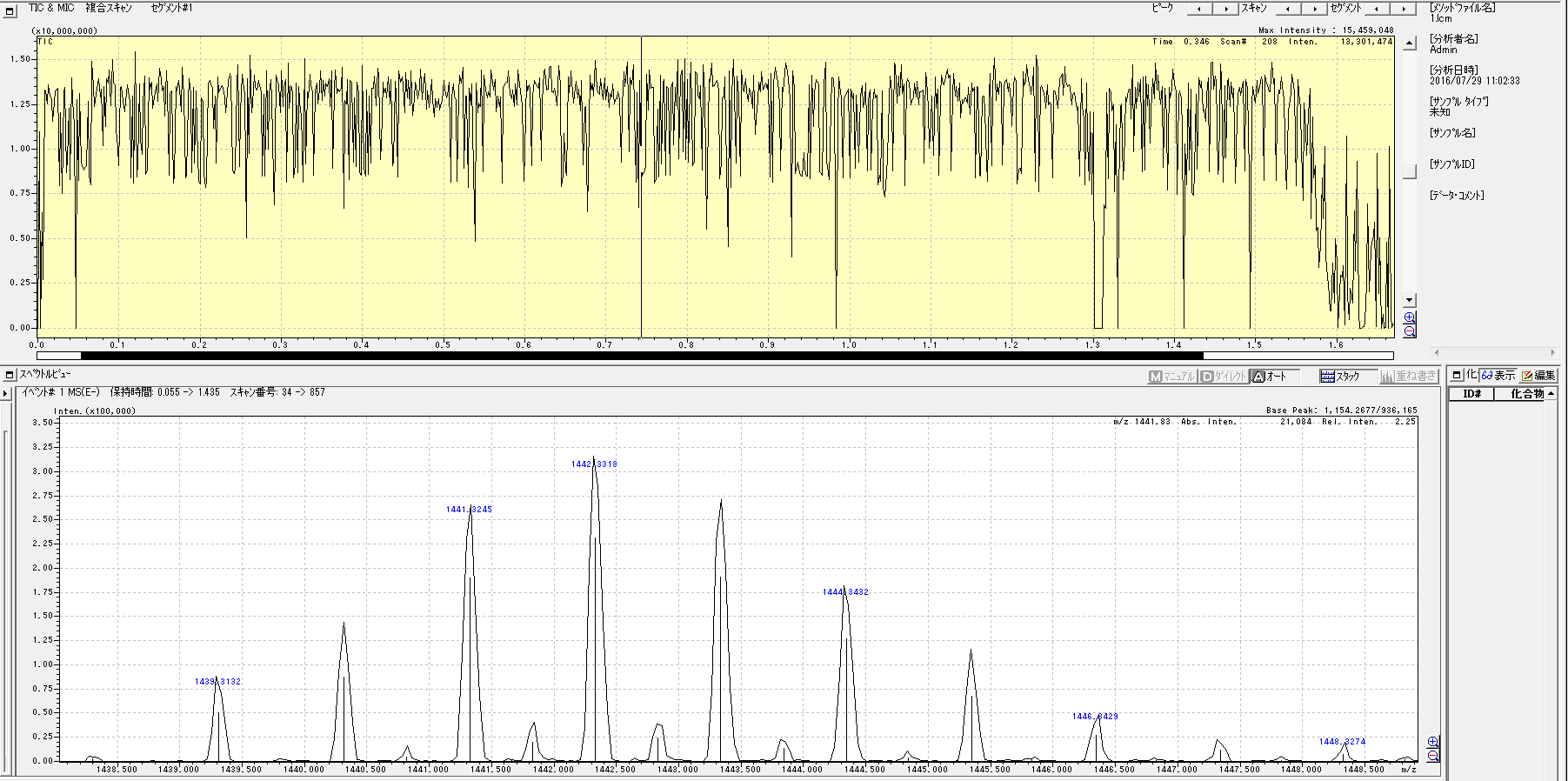


**HPLC data of 6 (HPLC-C).**

**
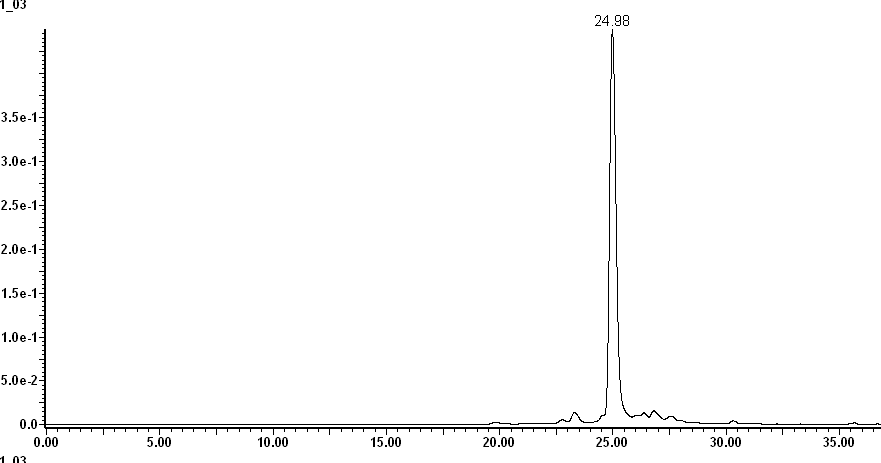
**
